# Combined inhibition of Bcl-2 family members and YAP induces synthetic lethality in metastatic gastric cancer with RASA1 and NF2 deficiency

**DOI:** 10.1186/s12943-023-01857-0

**Published:** 2023-09-20

**Authors:** Jong-Wan Kwon, Jeong-Seop Oh, Sang Hyeok Seok, Hyeok-Won An, Yu Jin Lee, Na Yun Lee, Taehun Ha, Hyeon Ah Kim, Gyeong Min Yoon, Sung Eun Kim, Pu-Reum Oh, Su-Hyung Lee, Dominic C. Voon, Dae-Yong Kim, Jun Won Park

**Affiliations:** 1 Division of Biomedical Convergence, College of Biomedical Science, Kangwon National University, 1, Kangwondaehak-Gil, Chuncheon-Si, Gangwon-Do 24341 Republic of Korea; 2 Department of Veterinary Pathology, College of Veterinary Medicine, Seoul National University, 1, Gwanak-Ro, Gwanak-Gu, Seoul, 08826 Republic of Korea; 3 Research Institute for Veterinary Science, College of Veterinary Medicine, Seoul National University, Seoul, 08826 Republic of Korea; 4 Section of Surgical Sciences, Epithelial Biology Center, Vanderbilt University Medical Center, Nashville, TN USA; 5 Cancer Research Institute, Kanazawa University, Kanazawa, Ishikawa 920-1192 Japan; 6 Innovative Cancer Model Research Unit, Institute for Frontier Science Initiative, Kanazawa University, Kanazawa, Ishikawa 920-1192 Japan

**Keywords:** Cancer stem cells, CRISPR/Cas9, Wnt pathway, YAP signaling

## Abstract

**Background:**

Targetable molecular drivers of gastric cancer (GC) metastasis remain largely unidentified, leading to limited targeted therapy options for advanced GC. We aimed to identify molecular drivers for metastasis and devise corresponding therapeutic strategies.

**Methods:**

We performed an unbiased in vivo genome-wide CRISPR/Cas9 knockout (KO) screening in peritoneal dissemination using genetically engineered GC mouse models. Candidate genes were validated through in vivo transplantation assays using KO cells. We analyzed target expression patterns in GC clinical samples using immunohistochemistry. The functional contributions of target genes were studied through knockdown, KO, and overexpression approaches in tumorsphere and organoid assays. Small chemical inhibitors against Bcl-2 members and YAP were tested in vitro and in vivo*.*

**Results:**

We identified *Nf2* and *Rasa1* as metastasis-suppressing genes through the screening. Clinically, *RASA1* mutations along with low *NF2* expression define a distinct molecular subtype of metastatic GC exhibiting aggressive traits. NF2 and RASA1 deficiency increased in vivo metastasis and in vitro tumorsphere formation by synergistically amplifying Wnt and YAP signaling in cancer stem cells (CSCs). NF2 deficiency enhanced Bcl-2-mediated Wnt signaling, conferring resistance to YAP inhibition in CSCs. This resistance was counteracted via synthetic lethality achieved by simultaneous inhibition of YAP and Bcl-2. RASA1 deficiency amplified the Wnt pathway via Bcl-xL, contributing to cancer stemness. *RASA1* mutation created vulnerability to Bcl-xL inhibition, but the additional NF2 deletion conferred resistance to Bcl-xL inhibition due to YAP activation. The combined inhibition of Bcl-xL and YAP synergistically suppressed cancer stemness and in vivo metastasis in RASA1 and NF2 co-deficiency.

**Conclusion:**

Our research unveils the intricate interplay between YAP and Bcl-2 family members, which can lead to synthetic lethality, offering a potential strategy to overcome drug resistance. Importantly, our findings support a personalized medicine approach where combined therapy targeting YAP and Bcl-2, tailored to NF2 and RASA1 status, could effectively manage metastatic GC.

**Supplementary Information:**

The online version contains supplementary material available at 10.1186/s12943-023-01857-0.

## Background

Gastric cancer (GC) is a highly aggressive malignancy and it is the fourth leading cause of cancer-related death worldwide [1, 2]. Despite advances in chemotherapy, patients with metastatic GC still have poor outcomes, with a 3-year overall survival of 5% [3, 4]. Targeted approaches based on genetic and molecular features of metastatic GC are urgently required to pave the way for effective therapy. However, options for targeted therapy in metastatic GC are limited. Identification of targetable molecular drivers of metastasis and the development of targeted therapies for metastatic GC are challenging on multiple fronts. Foremost, the molecular and genetic profiles of GC metastasis are not well characterized owing to mutational heterogeneity [5, 6]. Additionally, most somatic mutations in advanced GC arise in tumor suppressor genes [5], complicating efficient targeting and treatment. Furthermore, preclinical models that are genetically well defined and closely mimic human GC malignancy are scarce. Only few genetically well-defined immunocompetent mouse models demonstrating metastasis, which are essential for validating candidate genes associated with GC metastasis, have been reported [7–10]. Notably, advanced, metastatic mouse GC models have been developed through conditional mutations targeting the Wnt, Ras, and p53 pathways in gastric epithelia, expanding our understanding of GC metastasis [7]. Additionally, the *Cdh1*^F/+^;*Trp53*^F/F^;*Smad4*^F/F^ (ChetPS) GC mouse model represents a unique preclinical model that recapitulates the malignant progression of human GC [8, 11, 12]. The ChetPS GC mouse model has provided insights into GC metastasis and therapeutic vulnerability based on genetic features [9, 13]. Further comprehensive gene screening approaches to identify functional drivers of metastasis using such preclinical models may offer valuable clues for rationalized and personalized treatment options for metastatic GC.

Previous studies have identified the B-cell lymphoma 2 (Bcl-2) family of proteins, particularly Bcl-2 and Bcl-xL, as promising targets for suppressing gastrointestinal cancer stemness and metastasis [9, 14]. These are anti-apoptotic proteins that are frequently overexpressed in cancer cells and contribute to tumorigenesis, drug resistance, and metastasis [15]. Beyond a classical role in inhibiting apoptosis, these proteins also play non-canonical roles in promoting carcinogenesis by regulating other cellular processes, including proliferation and differentiation [15, 16]. Although inhibitors of Bcl-2 and Bcl-xL are effective in treating hematopoietic malignancies [17], efficacy is limited in primary solid cancers including GC partly because of a complex microenvironment that promotes cancer cell survival and resistance to treatment. Clinical trials of inhibitors of the Bcl-2 family in advanced GC have shown mixed results, and application in GC is currently limited [18, 19]. However, a potential window for targeting Bcl-2 family members may arise during metastasis of solid tumors [9], where circulating tumor cells or colonizing micrometastatic foci maintain a high level of cancer stemness and are less strongly influenced by the microenvironment, with similarities to hematopoietic malignancies. In addition, combining Bcl-2 family inhibition with other strategies can suppress metastasis in solid cancers [18]. Combination strategies guided by biomarkers to predict the response of inhibitors of the Bcl-2 family in the treatment of metastatic advanced GC have been suggested as a potential solution to the limited effectiveness of these inhibitors in GC.

CRISPR/Cas9 KO pooled libraries have proven to be powerful tools for genetic screening aimed at identifying key regulators involved in malignant progression [20, 21]. In this study, we utilized an in vivo ChetPS GC peritoneal dissemination model and successfully performed unbiased genome-wide CRISPR/Cas9 KO screening. We identified Ras p21 protein activator 1 (Rasa1) and neurofibromin 2 (Nf2*)* as metastasis-suppressing genes. Nf2 deficiency leads to the aberrant activation of cancer pathways, including the Hippo-YAP pathway, and promotes malignant progression in various types of tumor [22, 23]. Rasa1 is a regulator of Ras GDP/GTP, and mutations or aberrant expression of *RASA1* contribute to pathological processes in tumor formation, chiefly via aberrant activation of Ras/RAF/MEK/ERK or Ras/PI3K/AKT signaling [24]. *RASA1* was found to be frequently mutated in GC patients, and most of these mutations represent putative drivers and have attracted interest for further investigation [5, 25]. In non-small cell lung carcinomas, *RASA1* loss-of-function mutations activate the RAS/MAPK pathway, which may sensitize cells to trametinib, a MEK1/MEK2 kinase inhibitor [26]. However, the significance of *RASA1*-inactivating mutations in metastasis has not been characterized in solid cancers, including GC, compared with effects in primary tumors.

In the present study, we discovered that the presence of *RASA1* mutation along with low *NF2* expression can define a distinct molecular subtype of metastatic GC exhibiting aggressive traits. We demonstrated novel consequences of RASA1 and NF2 deficiency in amplifying GC stemness. This amplification is facilitated through the modulation of YAP and Wnt signaling interactions by Bcl-2 family members in CSC-specific nodes. Our findings suggest that the interaction between YAP and Bcl-2 family members can lead to synthetic lethality that would aid in overcoming drug resistance, and combined inhibition of these pathways might provide a selective and effective therapeutic strategy for highly metastatic GC with RASA1 and NF2 deficiency.

## Methods

Detailed methods and associated materials are provided in Supplementary Information (Additional file 1).

### Cells and organoid culture

The genetically engineered mouse GC cells, S1 and S1M, were established in earlier studies [8, 11]. Human GC cell lines including SNU-484 (00484), SNU-668 (00668), SNU-719 (00719), NCI-N87 (60113), and MKN-74 (80104), were obtained from the Korean Cell Line Bank (KCLB). Cells were maintained in RPMI-1640 medium supplemented with 10% fetal bovine serum (FBS), 2 mM L-glutamine and 1% penicillin–streptomycin (PS) at 37 °C in a 5% CO_2_ humidified incubator. The HEK-293 (21573) cell line was obtained from KCLB and maintained in DMEM supplemented with 10% FBS and 1% PS.

Mouse stomach organoids were primarily cultured directly from mouse stomach tissues, as described previously [13, 20]. To generate *Trp53* KO mouse stomach organoids (P organoid), the gastric epithelium of Cre-dependent Cas9^+^-*Trp53*^*fl*/*fl*^ mice was used. Once the organoids were established, a Cre-recombinase expression cassette was introduced into the organoids using transfection. Organoids were then grown with nutlin-3 for 1 week to select *Trp53*-KO clones.

### Establishment of KO cell lines

GC cell lines with stable Cas9 expression were established using the PiggyBac transposon system. sgRNA lentiviral vectors were generated following a previously published protocol [27]. The sgRNA sequences for both mouse (67988 and 1000000053, Addgene) and human (67989 and 1000000049, Addgene) were obtained from the gRNA library. For controls, a non-targeting sgRNA sequence (5'-GTGTAGTTCGACCATTCGTG-3') and an intact plasmid backbone with a guide sequence of 5'-GGGTCTTCGAGAAGACAC-3' were used. Generation of KO cells is further detailed in Additional file 1.

### *Preparation of pooled gRNA library and *in vivo* screening*

Mouse Improved Genome-wide Knockout CRISPR Library v2 was a gift from Kosuke Yusa (67988, Addgene) [27]. Briefly, 80% confluent HEK-293 T cells in 150 mm cell culture dishes (11151, SPL) were transfected in Opti-MEM (31985070, Gibco) using 15 µg of Genome-wide Knockout CRISPR Library plasmids, 11.25 µg pLP1, 11.25 µg pLP2, 7.5 µg pLP/VSVG, and Transporter 5 transfection reagent (26008–5, Polysciences). After 16 h, the medium was changed to DMEM (LM001-05, Welgene). After 72 h, viral supernatants were harvested and filtered through a 0.45 µm PES filter (166–0045, Nalgene). For large-scale screening, 24 h after transduction with polybrene (8 µg/ml, H9268, Sigma-Aldrich), cells were selected with puromycin (2 µg/ml, ant-pr-1, InvivoGen) and blasticidin (5 µg/ml, ant-bl-1, InvivoGen) for one passage. After the first passage, 10 million cells expressing sgRNAs were intraperitoneally injected into immunocompetent syngeneic mice. Then, 105 days post injection metastatic nodules in the peritoneal cavity were isolated and genomic DNA was extracted using the AccuPrep® Genomic DNA Extraction Kit (K-3032, Bioneer). We independently repeated the in vivo screening three times (*n* = 10, 15, and 10 for each set). The multiplicity of infection was maintained at < 0.3 during all procedures to prevent multiple transductions of the lentivirus.

#### Tumorsphere formation assay

GC cells dissociated with the TrypLE (Gibco) were passed through a 40-µm cell strainer to obtain single-cell suspensions and minimize the formation of doublets. Then, 10,000 cells were seeded in advanced DMEM/F-12 medium supplemented with 10 mM HEPES, 1 mM N-acetylcysteine, 50 ng/ml epidermal growth factor, 2 mM GlutaMAX, 2% B-27 supplement, and 1% N-2 supplement on low-attachment, 24-well plates. To prevent cell aggregation and promote the formation of intact tumorspheres derived from single cells, constant shaking at 80 rpm was applied (Supplemental Figure 1A). The growth pattern of the tumorspheres was monitored under light microscope (Supplemental Figure 1B). To determine the minimum size of tumorspheres, GFP^+^ and RFP^+^ cells were mixed, seeded, and cultured for 5 days. Tumorspheres larger than 50 µm exhibited a single color, and these tumorspheres were counted (Supplemental Figure 1, C). Cells were treated with drugs at the indicated concentrations and incubated for 5 days.

To evaluate the self-renewal capacity of the cultured tumorspheres of control, *Nf2*-, *Rasa1*-, double-KO S1 cells, subculturing was performed. The primary tumorspheres were collected and dissociated using TrypLE. Single cells were obtained using 40-µm cell strainer. An equal number of single cells were reseeded and cultured for an additional 5 days. The resulting secondary tumorspheres were counted and diameter was measured using ImageJ (https://imagej.nih.gov/ij/).

#### Statistics

Statistical analyses were conducted using GraphPad Prism 8 (GraphPad Software, CA, USA). Values exceeding two times the standard deviation were considered outliers and excluded from the dataset. To compare two groups, a two-tailed Student's t-test was used with significance considered at *P* < 0.05. For comparing three or more groups, one-way ANOVA was performed, followed by a post-hoc Tukey's HSD test for pairwise comparisons.

The probability of survival over time was estimated using Kaplan–Meier survival analysis, with GraphPad Prism 8 software used to generate survival curves and perform log-rank comparisons of survival between groups. Censored observations were taken into account in the analysis, with *P* < 0.05 considered significant.

IC50 values were calculated using GraphPad Prism 8 with a nonlinear regression analysis for dose–response curve fitting. Specifically, the PBS control was set at 100%, and the maximum dose for inhibiting cell viability was set at 0%. The data were then fitted to a dose–response curve to determine the IC50 value. All data are shown as mean ± SD of three technical replicates of one representative biological experiment.

To determine the significance of CRISPR screening results, individual KO of each candidate gene were compared with the results for control S1M cells using Fisher's exact test. For the statistical analysis of GC TMA data, we used IBM SPSS Statistics version 26.0 (IBM Japan, Tokyo, Japan). Fisher's exact test was used to identify correlations between the immunoreactivities of NF2, RASA1, and β-catenin with histological grades, TNM stages, and clinical stages.

## Results

### In vivo* genome-wide CRISPR/Cas9 screening identified Nf2 and Rasa1 as genes suppressing metastasis in GC*

**Fig. 1:**
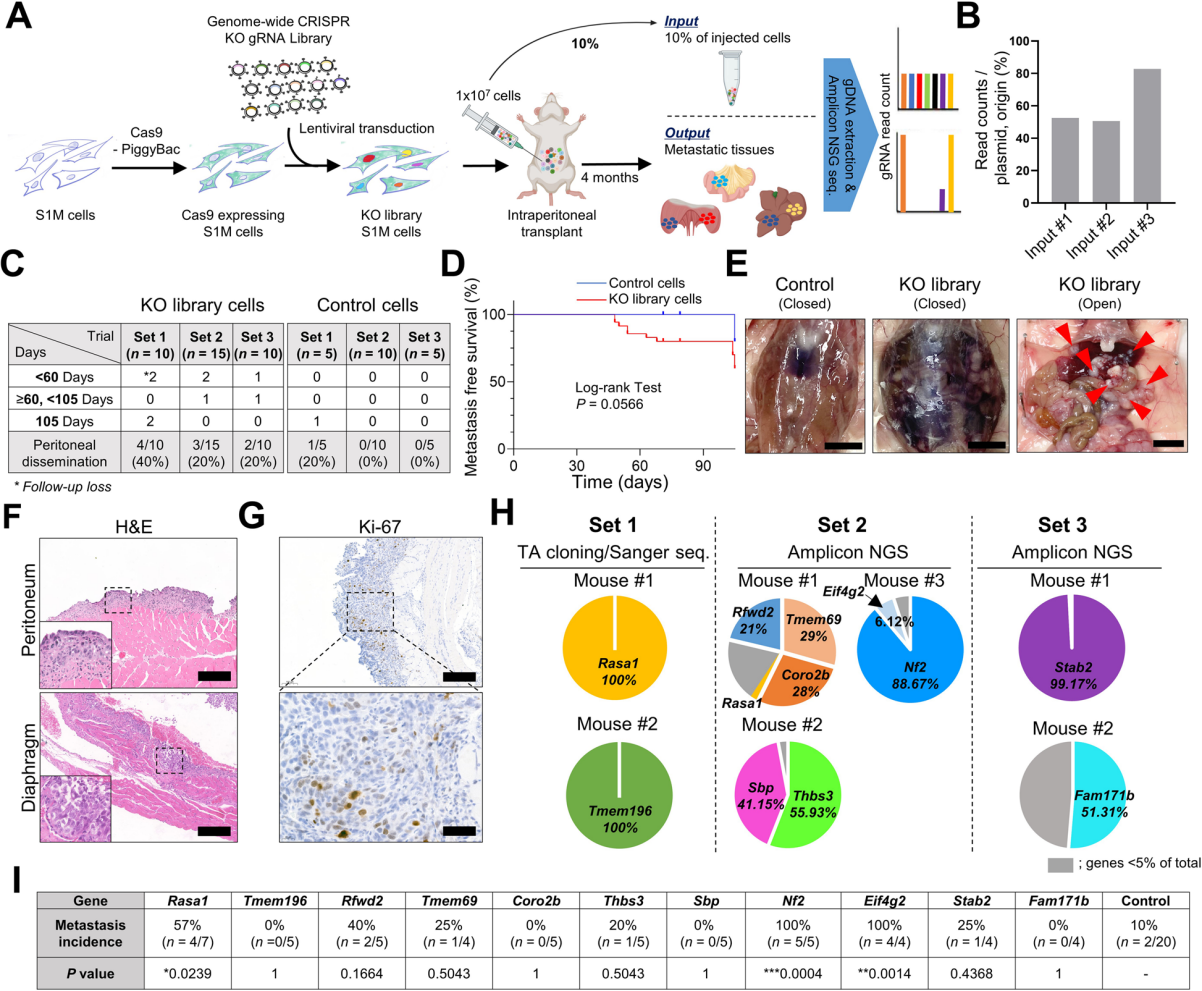
Identification of GC metastasis-suppressing genes using CRISPR KO screening. **A** Schematic of the in vivo genome-wide CRISPR KO screen using a peritoneal dissemination model. **B** KO library coverage rates based on amplicon next-generation sequencing (NGS) analysis of input samples from three independent experiments. **C** Peritoneal dissemination rates in syngeneic mice intraperitoneally injected with KO library and control S1M cells. Necropsy was performed either upon showing clinical signs of malignancy or after 105 days post-injection. **D** Kaplan–Meier survival analysis of metastasis-free survival percentage in syngeneic mice injected with KO library and control S1M cells. The data represent the combined results of three independent screenings of sets. *P* value, Log-rank test was performed to compare the metastasis-free survival. **E** Representative gross images at the point of necropsy of syngeneic mice after intraperitoneal injection with control and KO library S1M cells. Bar = 1 cm. **F** Representative H&E-stained images of metastatic foci developed at peritoneum and diaphragm of syngeneic mice intraperitoneally transplanted with KO library S1M cells. Bar = 200 μm. **G** Representative Ki-67 immunohistochemical images of metastatic foci in the peritoneum from syngeneic mice intraperitoneally transplanted with KO library S1M cells. Bar = (top) 200 μm, (bottom) 50 µm. **H** Enriched gRNA-targeted genes were analyzed using TA cloning with Sanger sequencing (set 1) and amplicon NGS analyses (sets 2 and 3). Each color in pie charts represents a specific gene targeted by the gRNA and shows the relative read counts obtained from each mouse with metastasis. Genes with read counts less than 5% of the total are represented in the gray area. **I** Incidence rates of metastasis after intraperitoneal injection with individual candidate genes-KO cells into syngeneic mice. *P* value, Fisher's exact test; compared with the results of control cells. In all experiments, Student's t-test was used to obtain the p-value for statistical analysis, unless otherwise specified

For systematic discovery of novel genes that suppress peritoneal dissemination of GC cells, we performed pooled genome-wide in vivo CRISPR loss-of-function screening using a lentiviral KO library [27]. This library comprises 90,230 single-guide RNAs (sgRNAs) targeting 18,424 mouse genes. We previously established GC cell lines from a ChetPS mouse GC, named S1 and its metastatic variant S1M, which could develop tumors when transplanted into syngeneic mice [11]. S1 cells, while highly malignant, exhibited metastatic capabilities in only a small subset of cells [11, 28]. S1M cells, considered a cancer stem cell (CSC)-like subpopulation, demonstrated enhanced tumorigenicity, metastatic potential, and chemotherapy resistance [9, 11, 28]. Genome-wide screening was performed using S1M cells. In our preliminary experiments, S1M cells rarely developed peritoneal dissemination when intraperitoneally injected into immunocompetent syngeneic hosts. Thus, we designed this screening as a negative selection approach to identify a small subset of winner cells with the KO of a metastasis-suppressing gene. As illustrated in Fig. 1A, three independent biological infections of the pooled lentiviral KO library at an MOI of 0.3 were performed in Cas9-expressing S1M cells. Cas9-expressing S1M cells were established by transfection with a PiggyBac plasmid carrying cDNA encoding Cas9, followed by an antibiotic selection process without single-cell cloning. Ten million pooled KO library cells were intraperitoneally injected into immunocompetent syngeneic mice (Fig. 1A). A portion of the injected cells (10%) was used as an initial representation of the pooled sgRNA library for subsequent amplicon sequencing (hereafter referred to as the input). The input samples comprised 52.5%, 50.6%, and 82.8% of the total sgRNAs in the first, second, and third trials, respectively (Fig. 1B). During the 105-day monitoring period after cell injection, 25.7% (9/35) of the mice injected with KO library cells formed detectable metastases in the abdominal cavity, whereas only 5% (1/20) of the mice injected with non-target control cells exhibited peritoneal dissemination (Fig. 1C). Mice injected with the KO library cells had a shorter survival time than did those injected with control cells as a result of peritoneal dissemination (Fig. 1D). Of the nine mice showing peritoneal dissemination in the KO-library-cell-injected group, five became moribund and were necropsied before the 105-day endpoint, exhibiting severe peritoneal dissemination and an abundance of bloody ascites. In the remaining four mice, intraperitoneal metastases were observed during necropsy at the endpoint (Fig. 1C). All mice injected with control cells survived and were scheduled for sacrifice at 105 days, with only one case of abdominal metastasis observed at necropsy (Fig. 1C). In mice injected with KO library cells, those showing peritoneal dissemination typically developed massive ascites, and metastases were mainly observed on the surface of the diaphragm, peritoneum, mesentery, and liver (Fig. 1E). Histopathologically, poorly differentiated cancer cells were seen to invade the muscle layer (Fig. 1F) and had a high proliferative rate, as assessed using Ki-67 IHC (Fig. 1G).

To identify genes involved in suppressing metastasis, we evaluated CRISPR hits and searched for genes targeted by gRNAs enriched in metastases. Metastatic foci from the liver, peritoneum, diaphragm, and mesentery were collected from the seven mice that exhibited peritoneal dissemination during three independent screenings. sgRNA cassettes integrated into the genome of metastatic cells were enriched and amplified using PCR on the gDNAs of the tissues collected (Fig. 1A). We examined gRNA sequences using TA cloning/Sanger sequencing or amplicon next-generation sequencing (NGS) (Fig. 1A). KO clones occupying more than 5% of the total winner cells in peritoneal metastases of each mouse were included in the candidate list (Fig. 1H). The gene list included *Nf2*, a well-known tumor suppressor, an outcome that validated the effectiveness of the screen in revealing genes altered by in vivo selection pressure. The gene list also contained novel candidates not previously associated with negative regulation of GC metastasis, emphasizing the novelty of our screening approach.

**Fig. 2:**
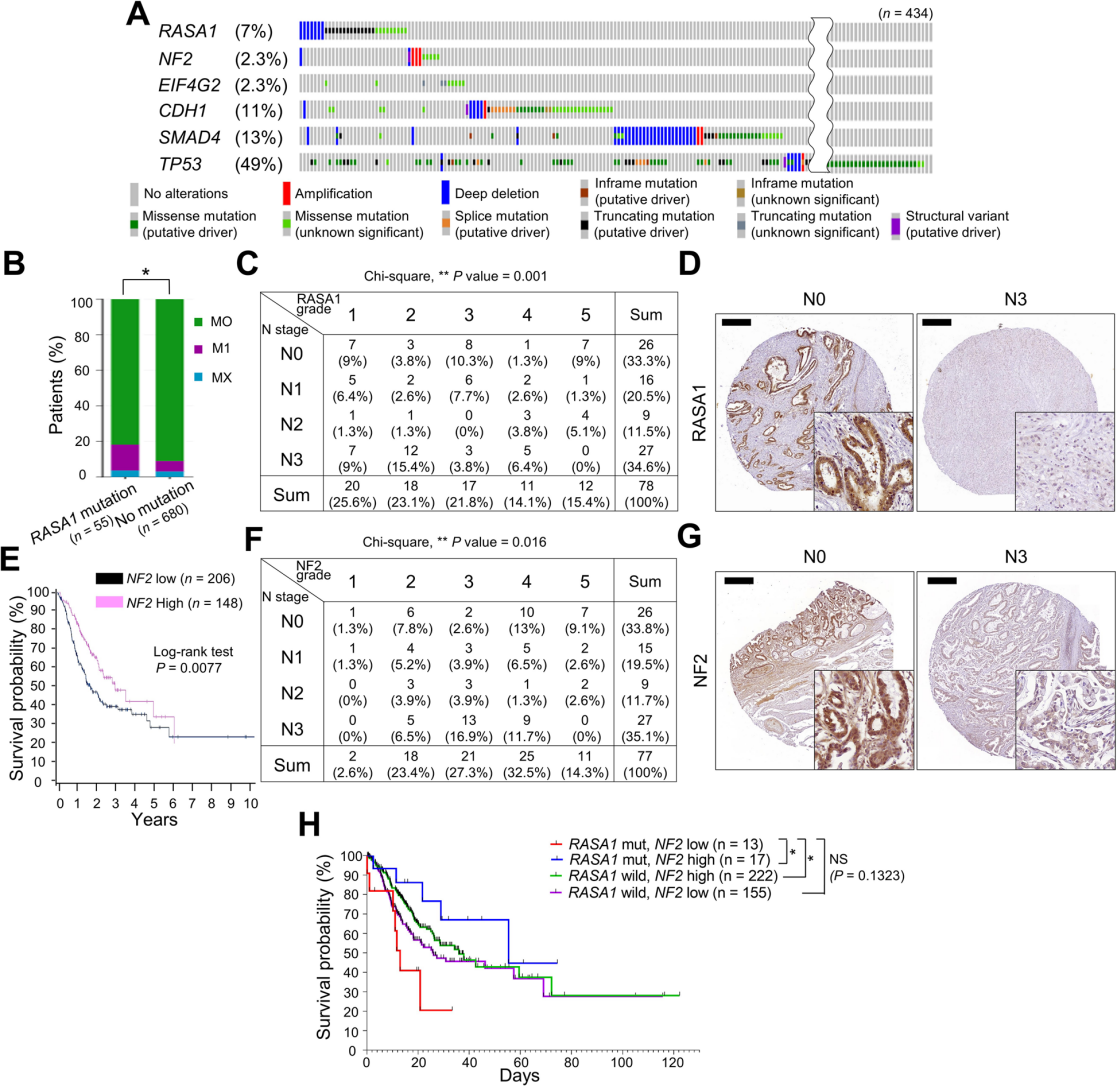
Clinical significance of NF2 and RASA1 deficiency in human GC. **A** Oncoprint of *RASA1*, *NF2*, *EIF4G2*, *CDH1*, *SMAD4*, and *TP53* mutations in GC. Each column represents one patient as identified in The Cancer Genome Atlas (TCGA) dataset. **B** Metastasis stage in GC patients depending on *RASA1* mutation status in TCGA dataset, classified as M0 (no metastasis), M1 (metastasis present), and MX (metastasis cannot be assessed). *P* value, chi-square test. **C** Decreased RASA1 expression was associated with the advanced N stage in human GC tissue microarray (TMA). *P* value, chi-square test. **D** Representative RASA1 immunohistochemical images of human GC TMA tissues according to N stage. **E** Kaplan–Meier plots for the overall survival of patients with GC according to *NF2* mRNA expression in the TCGA dataset. *P* value, log-rank test. **F** Decreased NF2 expression was associated with the advanced N stage in human GC TMA tissues. *P* value, chi-square test. **G** Representative NF2 immunohistochemical images of human GC TMA tissues according to N stage. **H** Kaplan–Meier plots for the overall survival of patients with GC according to *RASA1* mutation and *NF2* mRNA expression in TCGA dataset. *P* value, log-rank test

We validated the CRISPR screening results by performing individual KO of each candidate gene using the same lentiviral KO system. We confirmed the targeting efficiency of candidates including *Nf2* and *Rasa1* using western blotting (Supplemental Figure 2, A and C). Peritoneal transplantation of *Rasa1*-, *Nf2*-, and *Eif4g2*-KO S1M cells resulted in peritoneal dissemination in more than 50% of the mice, with statistical significance compared with results for control S1M cells (Fig. 1I and Supplemental Figure 2, B, D, and E). We prioritized *Nf2* and *Rasa1* for further study, given that *EIF4G2* has low clinical relevance given its infrequent driver gene mutations (Fig. 2A) and no discernable link to human GC survival or metastasis (*data not shown*).

To investigate the metastatic potential of *Rasa1* and *NF2* deficiency, we used another metastasis model: a splenic injection model that mimics the natural route of GC metastasis, which frequently proceeds to the liver via the portal vein. In this model, *Nf2*-and *Rasa1-*KO S1M cells led to metastases in the liver more efficiently than did control S1M cells after splenic injection (Supplemental Figure 3). Taken together, the gene KO experiments provided strong evidence that *Nf2* and *Rasa1* are important regulators of GC metastasis.

### RASA1 mutations along with low NF2 expression define a distinct molecular subtype of metastatic GC exhibiting aggressive traits

We investigated the clinical relevance of *NF2* and *RASA1* deficiencies in human GC using The Cancer Genome Atlas (TCGA) data [5, 29]. *RASA1* was mutated in 7% of the patients with GC (30/434), and most cases (21/30) involved putative driver mutations, such as deep deletions and truncating mutations (Fig. 2A). *RASA1* mutations did not exhibit a statistically significant correlation, including mutual exclusivity or co-occurrence, with *SMAD4*, *TP53*, and *CDH1* mutations, which are genetically deleted in S1M cells (Fig. 2A). The presence of *RASA1* mutations was correlated with distant metastasis in patients with GC (Fig. 2B). We further investigated the clinical significance of Rasa1 protein levels based on IHC analyses of human GC tissue microarray (TMA) slides (Fig. 2, C and D, and Supplemental Table 1). We identified a negative correlation between Rasa1 expression and N stage (*P* = 0.001) (Fig. 2, C and D), implying that downregulation of *RASA1* is associated with GC metastasis. In addition, significant correlations between Rasa1 levels and other clinicopathological variables, including histological grade, size, and stage, were observed (Supplemental Table 1), indicating the prognostic value of Rasa1 loss.

*NF2* was mutated in 2.3% of patients with GC (10/434)*,* and two cases had a deep deletion, a putative driver resulting in loss of function (Fig. 2A)*.* Although *NF2* driver mutations are infrequent, patients with GC with *NF2*-low characteristics as assessed based on RNA expression levels showed shorter survival times than did those with *NF2*-high characteristics (Fig. 2E). Nf2 protein expression was also significantly associated with N stage (*P* = 0.016), M stage (*P* = 0.001), and histological grade (Fig. 2, F and G, and Supplemental Table 2). Intriguingly, *RASA1*-mutated GC cases with low *NF2* expression showed notably reduced survival compared to other groups, such as *RASA1*-wild and *RASA1*-mutated/*NF2* high cases (Fig. 2H). Underscoring that *NF2* downregulation is a critical determinant of worse clinical outcomes in *RASA1*-mutated patients. This observation suggests a potential cooperative role for *RASA1* and *NF2* deficiency in GC progression. Collectively, these results indicate that *RASA1* and *NF2* deficiency can characterize molecular subtypes of metastatic GC that exhibit aggressive phenotypes.

Using an in vivo peritoneal seeding model with human GC cells, we evaluated the functional contribution of *RASA1* or *NF2* loss to human GC metastasis. To investigate whether *RASA1*- or *NF2*-KO clones had an advantage over wild-type clones in peritoneal propagation, an in vivo competitive assay was conducted using human GC cells. In our preliminary experiments, we evaluated multiple human GC cell lines to identify those capable of peritoneal dissemination in immunodeficient mice. We observed that SNU-484 cells successfully developed peritoneal dissemination when intraperitoneally injected into immunodeficient SCID mice. We generated *RASA1*- or *NF2*-KO SNU-484 cells (Supplemental Figure 4A) and injected them intraperitoneally into NSG or NOD-SCID mice, mixed in equal proportions with non-target control cells (Supplemental Figure 4B). The non-targeting gRNA sequences used for the control cells were designed not to recognize any sequences in the human genome [30]. Five weeks post-injection, we harvested ascites and organs that were sites of metastasis and extracted gRNAs to measure the relative ratio of targeting gRNAs and non-target gRNA, using previously established amplicon NGS or real-time PCR analysis methods [20]. Higher levels of *RASA1*- or *NF2*-targeting gRNAs than of non-target gRNA were detected (Supplemental Figure 4, C and D). Furthermore, *RASA1-*KO cells exhibited more peritoneal dissemination on injection into NSG mice than did control cells (Supplemental Figure 4, E–G). The results indicate that *RASA1* and *NF2* deficiency promotes cellular mechanisms that provide a competitive advantage during metastasis in human GC cells. The growth of SNU-484 cells in monolayers in the presence of 10% serum was not affected by *NF2*- or *RASA1*-KO (Supplemental 5A), suggesting that factors beyond mere growth in differentiation conditions contribute to enhancing cell viability during metastasis.

### NF2 and RASA1 deficiency cooperatively enhance Wnt and YAP signaling in cancer stem cells

**Fig. 3:**
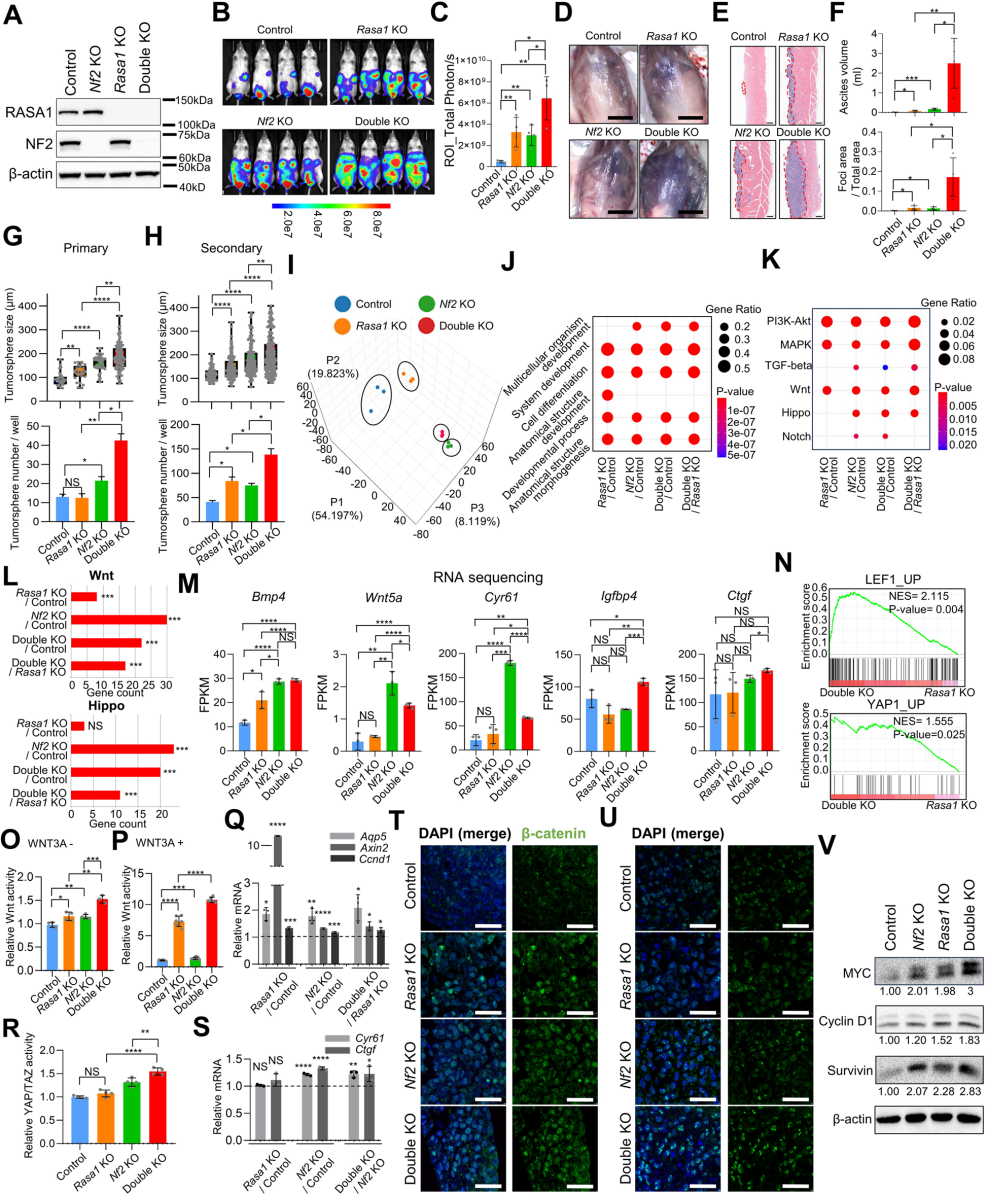
Cooperative enhancement of Wnt and YAP signaling in cancer stem cells by NF2 and RASA1 deficiency. **A** Western blot analysis of RASA1 and NF2 in control and KO S1M cells. **B-F** (**B**) Bioluminescence in vivo imaging of NOD-SCID mice 10 days after intraperitoneal transplantation of control, *Nf2*-, *Rasa1-* and *Nf2*/*Rasa1-double-*KO S1M cells (each of *n* = 4). (**C**) Total bioluminescence signals measured in each mouse. **D** Representative gross and (**E**) H&E images of each group. Red dashed lines indicate tumor area. Bar = (**D**) 1 cm, (**E**) 200 μm. **F** Effect of *Rasa1*- and *Nf2*-deficiency on peritoneal dissemination was evaluated using ascites volume (top) and number of macro-metastatic foci (bottom). **G** and **H** Sphere-forming assay using control, *Rasa*-, *Nf2*-, and *Rasa1*/*Nf2-*double-KO S1 cells. **G** Primary and (**H**) secondary tumorspheres (top) size and (bottom) number was measured. **I** Principal component analysis of RNA profiles obtained from control and KO S1 tumorspheres. Each dot represents a technical replication. **J** and **K** (**J**) GO and (**K**) KEGG pathway enrichment analysis of genes with significant expression changes (> threefold change) in KO cells compared to control cells. **L** Bar chart showing the differential activation of the Wnt and Hippo signaling pathways in KO cells compared to control cells, as determined by KEGG pathway analysis. **M** mRNA expression levels of YAP/TAZ-TEAD target genes in control, *Rasa1*-KO, *Nf2*-KO, and *Rasa1*/*Nf2*-double-KO S1 tumorspheres, as determined by RNA sequencing analysis. **N** Gene set enrichment analysis showing upregulation of Wnt and Hippo/YAP pathway-related genes in double-KO S1 tumorspheres compared to *Rasa1*-KO tumorspheres. **O** and **P** TOP-Flash luciferase reporter assay in control and KO S1M cells, depending on WNT3A stimulation; (**O**) conventional media, (**P**) 25 ng/ml of WNT3A. **Q** Relative mRNA expression of Wnt-dependent transcription (*Aqp5, Axin2, and Ccnd1*) in control and KO S1M cells (WNT3A, 25 ng/ml). **R** and **S** (**R**) HOP-Flash luciferase reporter assay and (**S**) relative mRNA expression of YAP-dependent transcription (*Ctgf* and *Cyr61*) in control, *Rasa1*-, *Nf2*-, and *Rasa1*/*Nf2-*double-KO S1 cells. **T** and **U** Immunofluorescence staining images of control and KO S1 tumorspheres using (**T**) active-β-catenin (green) and (**U**) YAP (green) with DAPI. Bar = 25 μm. **V** Western blot analysis of MYC, Cyclin D1 and Survivin in control and KO S1M cells (WNT3A, 25 ng/ml). Student's t-test was used for statistical analysis, unless otherwise specified

To investigate the individual and combined effects of *Nf2* and *Rasa1* loss on metastasis, we used a peritoneal dissemination model in immunodeficient NOD-SCID mice. For this, we generated control, *Nf2*-KO, *Rasa1-*KO, and double-KO S1M cells, each labeled with luciferase (Fig. 3A). After 10 days of peritoneal injection of S1M cells, we examined the bioluminescence signal and subsequently performed necropsy for histopathology. Based on luciferase bioluminescence assessment, we observed that both *Nf2-*KO and *Rasa1-*KO S1M cells demonstrated a significant increase in peritoneal dissemination, compared to control cells, with the double-KO S1M cells demonstrating the most pronounced level of metastasis (Fig. 3B and C). The volume of bloody ascites was remarkably increased in double-KO S1M cells compared to the control and single KO groups (Fig. 3D). Upon evaluating the number of metastatic sites and overall area affected by dissemination through H&E staining (Fig. 3E), we found that *Nf2-*KO and *Rasa1-*KO S1M cells demonstrated a significant increase in peritoneal dissemination compared to control cells. The double-KO S1M cells exhibited the most profound level of metastasis (Fig. 3F).

CSCs play a critical role in every step of the metastatic cascade, from cancer cell invasion to survival in the bloodstream, attachment and extravasation, and colonization of the host organ and the subsequent establishment of distant macrometastasis [31]. Consequently, we evaluated the capability of control, *Nf2-*KO, *Rasa1-*KO, and double-KO cells to form tumorspheres. For the tumorsphere formation assay, we selected S1 rather than S1M cells because, unlike S1M cells, which maintain the CSC phenotype even in a monolayer cell culture, S1 cells contain a small subpopulation that remains as CSCs in monolayer cultures [9, 28]. S1 cells were also preferable given their ability to consistently form rounder tumorspheres, as against S1M cells. During primary tumorsphere formation, *Nf2-*KO and *Rasa1-*KO S1 cells showed increased tumorsphere size compared to the control (Fig. 3G). During secondary tumorsphere formation, both *Nf2-*KO and *Rasa1-*KO cells exhibited an increase in size and number, compared to the control (Fig. 3H). The double-KO cells showed an increase in both size and number during primary and secondary tumorsphere formation, compared to control, *Nf2-*KO, and *Rasa1-*KO cells (Fig. 3G and H). These results suggest that the loss of *Rasa1* and *Nf2* cooperatively enhanced cancer stemness. However, the control, *Nf2-*KO, *Rasa1-*KO, and double-KO cells did not affect S1 cell growth in monolayers in the presence of 10% serum (Supplemental Figure 5), thereby highlighting the specificity of *Rasa1* and *Nf2* loss in promoting GC stemness.

Using RNA-sequencing analysis, we investigated the transcriptomic differences among control, *Nf2-*KO, *Rasa1-*KO, and double-KO S1 tumorspheres (GEO239457). A principal component analysis highlighted distinct clustering of the control, *Nf2-*KO, *Rasa1-*KO, and double-KO S1 tumorspheres (Fig. 3I). Distinct changes in the transcriptome induced by *Rasa1-* and *Nf2-*KO were observed (Supplemental Figure 6A). Gene Ontology (GO) enrichment analysis of genes displaying >3-fold change in expression across comparisons demonstrated prominent shifts within biological processes (Supplemental Figure 6B). Specifically, the processes of development and morphogenesis underwent conspicuous changes due to *Nf2*-and *Rasa1*-KO (Fig. 3J). This further strengthens our hypothesis that NF2 and RASA1 deficiency induces changes in cancer stemness, as CSCs are capable of mimicking and modifying normal developmental processes to support cancer cell survival and proliferation [32].

Kyoto Encyclopedia of Genes and Genomes (KEGG) pathway enrichment analysis of differentially expressed genes with > threefold changes in *Rasa1-* and *Nf2-* KO tumorspheres compared to that in controls revealed the most pronounced changes in Pathways in cancer (KEGG 05200) (Supplemental Figure 6C). Consequently, a concentrated examination of oncogenic signaling pathways [29] indicated that *Rasa1-, Nf2-,* double*-* KO tumorspheres activated various oncogenic pathways when compared to controls (Fig. 3K). The loss of RASA1 provided cancer cells with a survival advantage via the Ras/RAF/MEK/ERK pathway [24]. As anticipated, the MAPK signaling pathway emerged as one of the most significantly affected in *Rasa1-*KO tumorspheres (Fig. 3K). NF2 deficiency activates Hippo/YAP signaling, which facilitates tumorigenesis and metastasis [23]. In line with this, we observed distinct enrichment of the Hippo/YAP signaling pathway in the context of *Nf2*-KO (Fig. 3, K and L). Upon examining the YAP/TAZ-TEAD target gene [33], we indeed found significant increases or upward trends of these target genes in *Nf2*- KO and *Nf2*/*Rasa1* double- KO tumorspheres compared to controls (Fig. 3M). In GC patients with *RASA1* mutations, survival is significantly reduced when *Nf2* expression is low (Fig. 2H). To investigate the signaling pathways involved in this process, the KEGG results clearly highlighted the significance of Wnt and Hippo signaling pathways in double-KO versus *Rasa1*-KO tumorspheres (Fig. 3, K and L). The Wnt signaling pathway is involved in the regulation and support of CSCs in GC and plays an essential role in gastric carcinogenesis [34–36]. Wnt and YAP signaling activities in CSCs and the intimate crosstalk between these two signals play an important role in CSC biology [37]. Further, we performed gene set enrichment analysis (GSEA) on double-KO vs. *Rasa1-*KO tumorspheres. Gene sets related to Hippo/YAP signaling were significantly enriched in double-KO tumorspheres compared to those in *Rasa1*-KO tumorspheres (Fig. 3N). Gene sets linked to Wnt signaling were also notably elevated in double-KO tumorspheres relative to those in *Rasa1*-KO tumorspheres (Fig. 3N). Based on these findings, we hypothesized that the simultaneous KO of both genes may have a synergistic or additive effect on the upregulation of Wnt and YAP signaling, compared to the individual KO of *Nf2* or *Rasa1*.

To validate this hypothesis, we used a lentivirus-based firefly luciferase reporter to analyze Wnt activity in control, *Nf2-*KO, *Rasa1-*KO, and double-KO S1M cells. We found that both *Nf2*-and *Rasa1*-KO led to a greater enhancement of Wnt signaling compared to control S1M cells (Fig. 3O). Notably, the *Rasa1*-KO cells demonstrated a significant amplification of Wnt signaling in response to WNT3A treatment (Fig. 3P). Furthermore, the double-KO cells displayed a synergistic increase in Wnt activity (Fig. 3O and 3P). Enhanced Wnt-dependent transcription (*Aqp5*, *Axin2*, and *Ccnd1*) was also observed in the *Nf2-*KO, *Rasa1-*KO, and double-KO S1M cells, corroborating the results of the Wnt reporter assay (Fig. 3Q). These results provide compelling evidence that *Nf2* and *Rasa1* deficiency amplifies the Wnt pathway.

Using a lentivirus-based firefly luciferase reporter, we examined YAP activity in control, *Nf2-*KO, *Rasa1-*KO, and double-KO S1M cells. YAP signal activation was observed in *Nf2* deficient *Nf2-*KO and double-KO S1M cells, with the double-KO demonstrating a synergistic increase (Fig. 3R). When we examined Hippo/YAP target gene expression using reverse transcription-quantitative polymerase chain reaction (RT-qPCR), we confirmed the upregulated expression of target genes, such as *Ctgf* and *Cyr61,* in *Nf2* deficient *Nf2-*KO and double-KO S1M cells (Fig. 3S). Western blotting revealed that *Nf2* ablation in both mouse and human GC cells led to decreased phosphorylation of Ser127 of YAP (Supplemental Figure 7, A and B), which inhibited nuclear YAP translocation and transcription. The total amount of YAP/TAZ increased, suggesting increased YAP signaling. YAP activation is known to increase anoikis resistance [38]. When we induced anoikis by seeding S1M cells onto low-attachment plates after single-cell dissociation, *Nf2*-KO reduced anoikis compared with results for control cells (Supplemental Figure 8A). *Nf2*-KO dramatically increased colonization on soft agar in S1M cells (Supplemental Figure 8B). *Yap1* KO reduced the increased colonization by *NF2*-KO cells (Supplemental Figure 8C). These findings suggest that increased anoikis resistance because of *Nf2* deficiency occurs via YAP activation and can potentially contribute to the increased numbers of *Nf2-*KO tumorspheres.

Through immunofluorescence (IF) analyses for active β-catenin and YAP in tumorspheres, we consistently observed more frequent nuclear accumulation of β-catenin in *Nf2-*KO and *Rasa1-*KO tumorspheres, with the highest frequency seen in the double-KO tumorspheres (Fig. 3T). Nuclear YAP accumulation was predominantly observed in *Nf2* deficient *Nf2-*KO and double-KO S1 tumorspheres (Fig. 3U). Western blotting revealed upregulated expression of MYC, Cyclin D1 (CCND1) and Survivin, which are target genes of Wnt and YAP signaling, as a consequence of *Nf2*-and *Rasa1*-KO (Fig. 3V).

However, IF analyses for β-catenin in metastatic foci showed markedly less nuclear accumulation of these proteins, almost to the point of being undetectable, compared to that in tumorspheres, irrespective of *Nf2* and *Rasa1* status (Supplemental Figure 9A). Although there was a tendency toward increased nuclear accumulation of YAP in Nf2 deficient *Nf2*-KO and double-KO metastatic foci, and a significant difference was observed between the control and double-KO metastatic foci, it was considerably less than the accumulation in tumorspheres (Supplemental Figure 9, B and C). Moreover, Wnt and YAP signaling in cancer cells derived from single-cell dissociation of peritoneal metastatic foci was substantially less than that in cancer cells obtained from ascites, as evidenced by a lentivirus-based firefly luciferase reporter (Supplemental Figure 9D). IHC of the Wnt target, Cyclin D1, and the proliferation marker, Ki-67, in metastatic foci of the peritoneal dissemination model of control, *Nf2-*KO, *Rasa1-*KO, and double-KO S1M cells revealed no significant disparities among the groups (Supplemental Figure 9, E and F). It's a well-established fact that CSCs constitute a minor subpopulation in tumor tissues, including primary and secondary tumors, while differentiated cancer cells make up the majority. Therefore, we speculate that these differentiated cancer cells in metastatic tumors exhibit low YAP and Wnt activity and may be less reliant on YAP and Wnt signaling regulated by the loss of NF2 and RASA1, compared to CSCs.

To validate our hypothesis further, we used a subcutaneous transplantation model, which naturally metastasizes to the lungs. Although *Rasa1-*KO S1M cells did not show significant differences from controls in terms of subcutaneous tumor growth, they did result in substantially increased lung metastases (Supplemental Figure 10, A and B). These findings indicate that *Rasa1*-KO contributes more to the metastasis-prone CSC-like cells, rather than to the differentiated cancer cells, which constitute the bulk of the primary tumor. Taken together, NF*2* and RASA1 loss cooperatively enhanced Wnt and YAP signaling in CSCs, thereby increasing metastasis.

### NF2 deficiency enhances Bcl-2-mediated Wnt signaling, contributing to cancer stemness

**Fig. 4:**
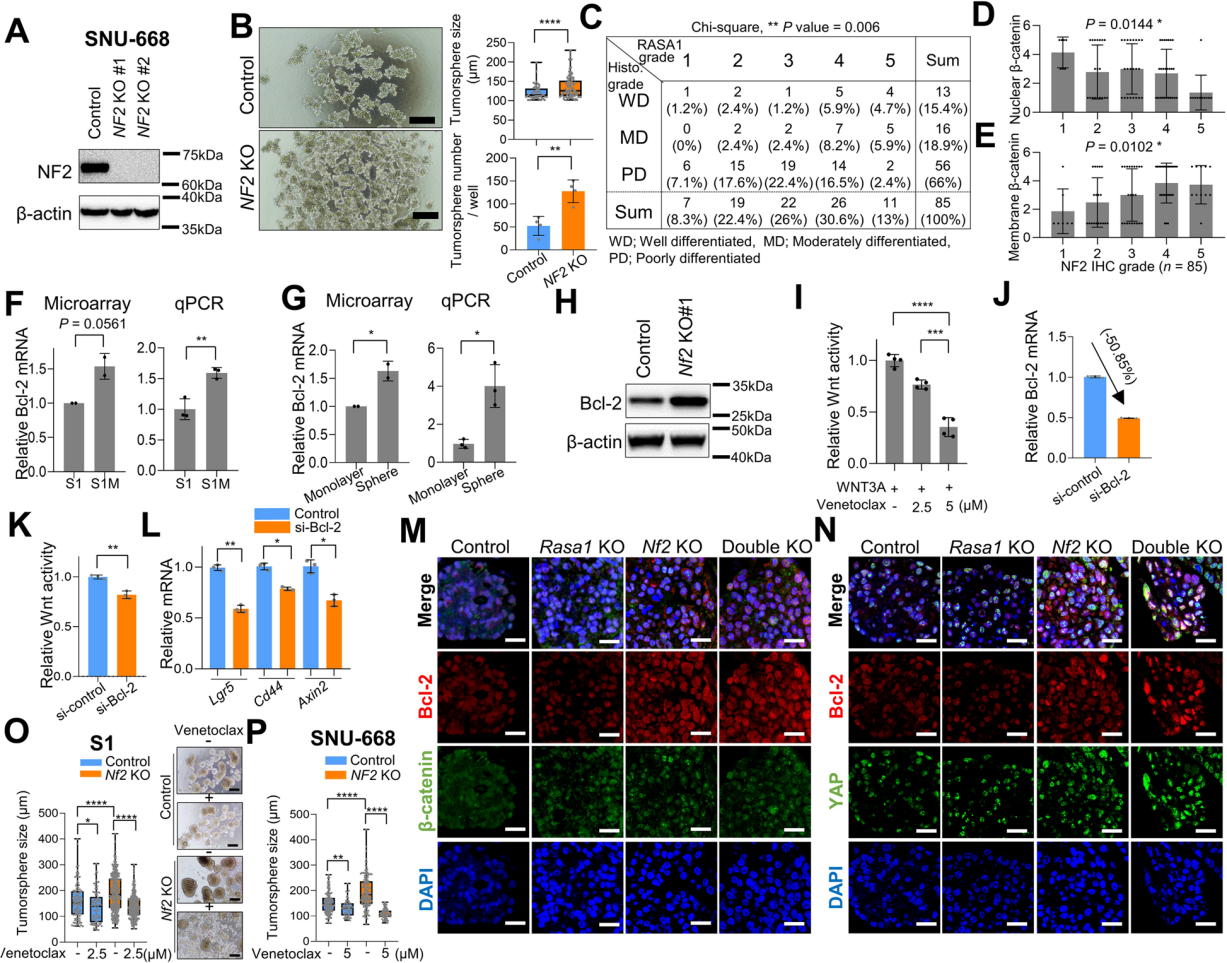
NF2 deficiency induces GC stemness and Wnt signaling via Bcl-2. **A** Western blot analysis of NF2 in control and *NF2*-KO SNU-668 cells. **B** Sphere-forming assay using control and *NF2*-KO SNU-668 cells. (left) Representative images of tumorsphere and (right) tumorsphere size (top) and number per well (bottom). Bar = 200 µm. **C** Low NF2 expression is associated with high histological grade in human GC tissue microarray (TMA) data. *P* value, chi-square test. **D** and **E** Immunohistochemical analysis of β-catenin expression in human GC TMA tissues according to NF2 expression. **D** Nuclear β-catenin, one-way ANOVA, *P* value = 0.0144, F = 3.320, (**E**) Membrane β-catenin, one-way ANOVA, *P* value = 0.0102, F = 3.552. **F** and **G** Microarray and reverse transcriptase-quantitative PCR (RT-qPCR) analysis of relative Bcl-2 mRNA expression in (**F**) monolayer culture of S1 and S1M cells and (**G**) monolayer and tumorsphere of S1 cells. **H** Western blot analysis of Bcl-2 in control and *Nf2*-KO S1M cells. **I** TOP-Flash luciferase reporter assay measuring Wnt pathway activity in venetoclax-treated S1M cells. Venetoclax was treated at the indicated concentrations for 24 h. **J** RT-qPCR analysis confirming Bcl-2 knockdown (KD) efficiency in S1M cells. **K** TOP-Flash luciferase reporter assay measuring Wnt pathway activity in control and Bcl-2 KD S1M cells. **L** Expression levels of Wnt-dependent transcription (*Lgr5*, *Cd44*, and *Axin2*) in control and Bcl-2 KD S1M cells. **M** and **N** Representative immunofluorescence images displaying Bcl-2 (red) paired with active-β-catenin (green) (**M**) and Bcl-2 (red) paired with YAP (green) (**N**) across control, *Rasa1*-KO, *Nf2*-KO, and *Rasa1*/*Nf2*-double-KO S1 tumorspheres. Bar = 25 µm. **O** and **P** Sphere-forming assay using control and *Nf2*-KO S1 (**O**) and control and *NF2*-KO SNU-668 (**P**) cells. Venetoclax treated at indicated concentrations for 5 days. Student's t-test was used for statistical analysis, unless otherwise specified

We proceeded to investigate the significance of NF2 loss in human GC tissues and cells. As observed in mouse GC cells, *NF2*-KO increased the size and number of tumorspheres in human GC SNU-668 cells (Fig. 4A and B). As assessed using TMA analyses of human GC tissues, histologically high-grade tumor cells showed significantly lower NF2 expression than did low-grade tumor cells (Fig. 4C), implying that *NF2* loss might be associated with tumor cell differentiation. Tumor progression including metastasis is driven by a gradual loss of a differentiated phenotype in parallel with the acquisition of CSC-like features [39, 40]. Activation of the Wnt signaling pathway causes β-catenin, which is normally located at the cell membrane, to accumulate in the nucleus. TMA analyses of human GC tissues also showed negative and positive correlations of NF2 expression with β-catenin nuclear expression and membrane β-catenin levels, respectively (Fig. 4, D and E, Supplemental Figure 11 and Supplemental Table 2).

Physiologically, YAP/TAZ counteract Wnt/β-catenin signaling by inducing the production of secreted Wnt inhibitors [33]. Similarly, our RNA-sequencing data revealed relatively higher expression levels of several secreted Wnt inhibitors, including *Wnt5a*, *Bmp4*, and *Cyr61,* in *Nf2-*KO tumorspheres, where YAP signaling was activated, than in control tumorspheres (Supplemental Figure 12A). Despite the increased production of secreted Wnt inhibitors, the activation of the Wnt signal after NF2 loss implied a dysregulation in the balance between Wnt and YAP signaling caused by NF2 deficiency.

To elucidate how *Nf2* loss results in enhanced Wnt signaling, we focused on Bcl-2—a downstream effector of YAP signaling [41]—given that we had previously found that Bcl-2 family members can modulate Wnt signaling and thereby regulate CSC activity in gastrointestinal malignancies [9, 14]. mRNA expression levels of Bcl-2 were higher in CSC-like S1M cells than in the parental S1 cells [28] (Fig. 4F). Additionally, Bcl-2 mRNA levels were higher in S1 tumorspheres grown under serum-free conditions than in monolayer S1 cells grown in the presence of 10% serum (Fig. 4G). Using western blotting, we observed upregulation of Bcl-2 expression in *Nf2*-KO S1M cells (Fig. 4H). Luciferase reporter assays showed that the elevated Wnt signaling activity, induced by *Nf2*-KO, was reduced in S1M cells following treatment with venetoclax, a specific inhibitor of Bcl-2 (Fig. 4I). To rule out the possibility of off-target effects of venetoclax, we performed knockdown (KD) experiments targeting Bcl-2 (Fig. 4J). We confirmed a reduction in Wnt signaling pathway activity and Wnt target gene expression upon Bcl-2 KD (Fig. 4, J-L). IF results indicated that both Nf2-KO and double-KO S1 tumorspheres exhibited amplified nuclear presence of YAP and elevated Bcl-2 expression, which co-localized with nuclear β-catenin, compared to *Rasa1*-KO and control tumorspheres (Fig. 4, M and N). In *Nf2*-KO and double-KO metastatic foci, we observed a consistent pattern with a more pronounced nuclear localization of YAP and increased Bcl-2 expression compared to *Rasa1*-KO and control counterparts (Supplemental Figure 13A-C). Venetoclax, a Bcl-2 inhibitor, reduced the increased tumorsphere size more efficiently in *Nf2*-KO S1 and *NF2*-KO SNU-668 cells than in control cells (Fig. 4, O and P), indicating that Bcl-2 mediates the increased cancer stemness induced by NF2 deficiency.

### Bcl-2 and YAP signaling regulate synthetic lethality in Nf2 deficiency

**Fig. 5:**
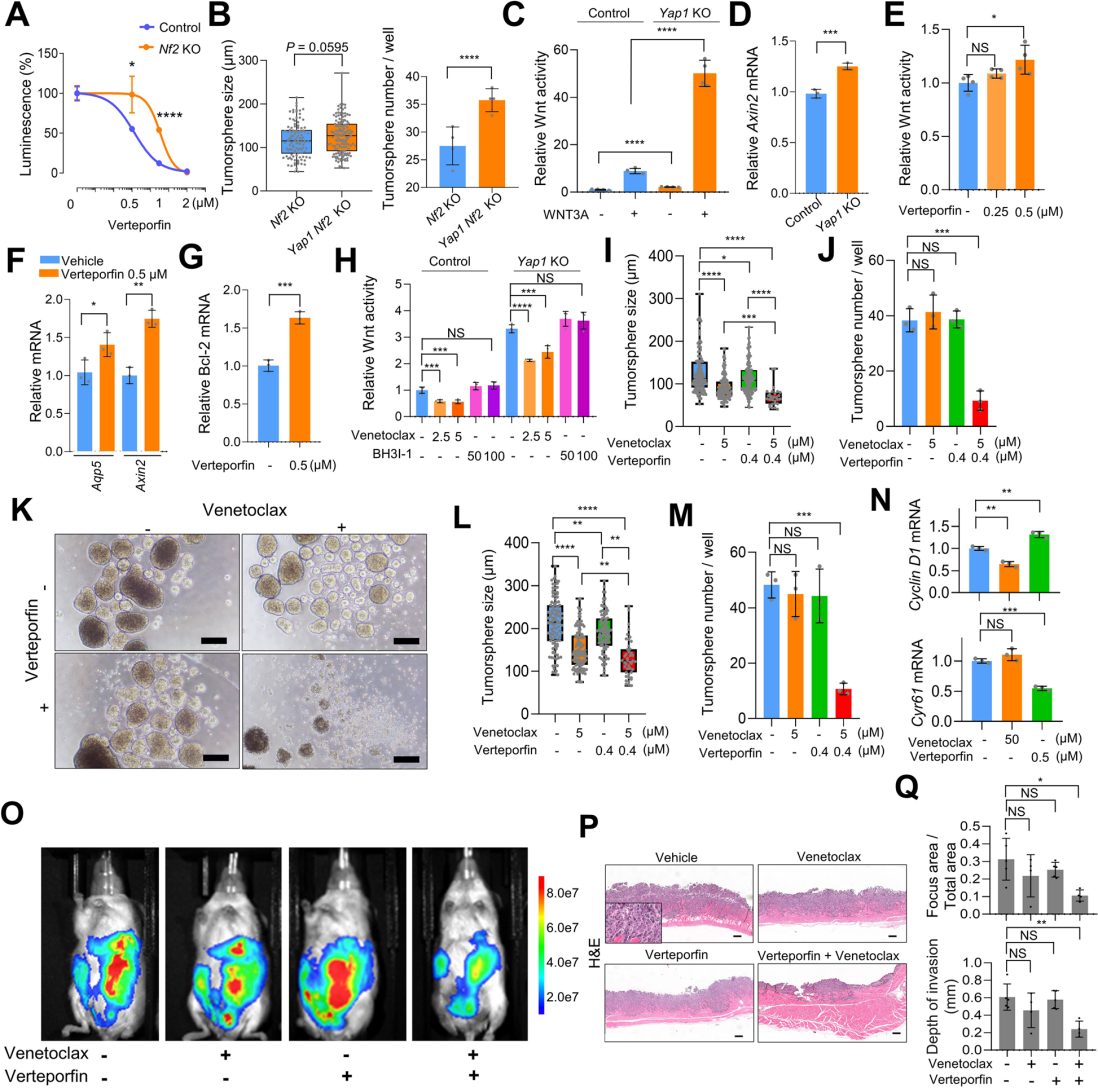
Synthetic lethality regulated by Bcl-2 and YAP signaling. **A** Relative cell viability of control and *Nf2*-KO S1 tumorspheres using luciferase ATP cell viability assay. Verteporfin was treated for 5 days at indicated doses. **B** Sphere-forming assay using *Nf2*-KO and *Nf2*/*Yap1-*double-KO S1 cells. Tumorspheres diameter (left) and counts (right). **C** TOP-Flash luciferase reporter assay in control and *Yap1*-KO S1M cells. **D** Relative mRNA expression of Wnt-dependent transcription (*Axin2*) in control and *Yap1*-KO S1M cells. **E** TOP-Flash luciferase reporter assay in S1M cells, verteporfin treated for 48 h at indicated doses. **F** Relative mRNA expression of Wnt-dependent transcription (*Aqp5* and *Axin2*) in S1M cells treated with verteporfin (0.5 µM) for 48 h. **G** Relative Bcl-2 mRNA expression in *Nf2*-KO S1 cells treated with verteporfin for 48 h. **H** TOP-Flash luciferase reporter assay in control and *Yap1*-KO S1 cells treated with venetoclax and/or BH3I-1 for 48 h. **I**, **J** and **K** Sphere-forming assay of S1 cells treated with venetoclax (5 µM) and/or verteporfin (0.4 µM) for 5 days. Tumorsphere size (**I**) and number (**J**). **K** Representative images of S1 tumorspheres. Bar = 200 µm. **L** and **M** Sphere-forming assay using MKN-74 cells. Venetoclax (5 µM) and/or verteporfin (0.4 µM) treated for 5 days. Tumorsphere size (**L**) and number (**M**). **N** Relative mRNA expression of (top) *Ccnd1* and (bottom) *Cyr61* in S1M cells treated with venontoclax and/or verteporfin at indicated dose for 48 h. **O**, **P** and **Q** *Nf2-*KO S1M cells were peritoneally injected in NOD-SCID mice with treatment of control (*n* = 5), venetoclax (*n* = 4, 12 mg/kg, p.o.), verteporfin (*n* = 5, 10 mg/kg, i.p.), and combination of venetoclax and verteporfin (*n* = 4). **O** epresentative bioluminescence imaging of NOD-SCID mice 8 days after intraperitoneal transplantation of *Nf2*-KO S1M cells with drug treatment. **P** H&E images of peritoneal metastatic tissues. Bar = 200 µm. **Q** Proportion of metastatic foci area (top) and depth of invasion (bottom) depending on treatment. Student's t-test was used for statistical analysis

As NF2 deficiency activated YAP signaling (Fig. 3), we expected that NF2 deficient cells might be sensitive to YAP blocking. Verteporfin is currently the most widely used small-molecule inhibitor of YAP; it directly binds to YAP and prevents interaction with transcriptional coactivators. Unexpectedly, as assessed using an ATP cell viability assay, *Nf2*-KO tumorspheres exhibited more resistance to verteporfin than did control tumorspheres (Fig. 5A). Furthermore, additional deletion of *Yap1* did not reduce the increase in tumorsphere size conferred by *Nf2*-KO in S1 cells but rather led to an increase in the number of tumorspheres (Fig. 5B). These results imply that NF2 deficient cells may employ alternative pathways to preserve their stemness under YAP blocking. The YAP/TAZ signaling pathway counteracts Wnt/β-catenin signaling by promoting the production of secreted Wnt inhibitors [33]. We noted reduced expression of several Wnt inhibitors, including *Cyr61*, *Igfbp4*, *Ctgf*, and *Wnt5a*, in cells exposed to a YAP inhibitor or in *Yap1-*KO cells (Supplemental Figure 12B). Furthermore, considering evidence that *Nf2* deficiency could bolster Wnt signaling via Bcl-2 induction (Fig. 4), we investigated whether Wnt signaling contributes to resistance to YAP blocking. Notably, *Yap1*-KO significantly amplified Wnt signaling (Fig. 5C) and the expression of Wnt target gene (Fig. 5D) in S1M cells. Verteporfin also increased Wnt signaling (Fig. 5E) and the expression of Wnt target genes (*Aqp5* and *Axin2*) (Fig. 5F) in S1M cells. Therefore, it is plausible that both upregulated Bcl-2 expression and diminished antagonism by Wnt inhibitors result in enhanced Wnt signaling upon YAP inhibition.

Therapeutically exploiting this antagonism by Wnt-inhibitor downregulation poses significant challenges. Thus, we focused on Bcl-2, which is upregulated during YAP signaling inhibition, as this may present viable targets for therapeutic interventions. To determine if the observed increase in Wnt signaling after YAP inhibition was because of the Bcl-2 induction caused by *Nf2* loss, we evaluated Bcl-2 expression. Bcl-2 induction in *Nf2*-KO cells was further enhanced by verteporfin treatment (Fig. 5G). Venetoclax, but not BH3I-1, a Bcl-xL inhibitor, suppressed Wnt signaling enhanced by *Yap1*-KO (Fig. 5H), indicating that Bcl-2 mediates Wnt upregulation following YAP blocking.

Based on these findings, we reasoned that combination therapy targeting Bcl-2 and YAP might effectively reduce cancer stemness. Combined treatment with verteporfin and venetoclax had a highly synergistic effect in inhibiting tumorsphere formation in mouse S1 (Fig. 5, I-K) and human MKN-74 cells (Fig. 5, L and M), emphasizing that synthetic lethality was regulated by Bcl-2 and YAP signaling. The treatment with verteporfin resulted in a decrease in the expression of the YAP target gene *Cyr61* and an elevation in the Wnt target gene *Ccnd1* (Fig. 5N). Moreover, treatment with the venetoclax led to a decrease in *Ccnd1* expression (Fig. 5N). We further investigated the in vivo effects of a combination of verteporfin and venetoclax in highly metastatic GC with *Nf2* deficiency. Seven days after peritoneal injection of 5 × 10^6^ *Nf2*-KO S1M cells labeled with luciferase into NOD-SCID mice, we examined the bioluminescence signal and subsequently performed necropsy for histopathology. Single-drug treatments targeting YAP or Bcl-2 did not significantly suppress peritoneal dissemination, as assessed using Luciferase bioluminescence and examination of the peritoneal dissemination area (Fig. 5, O-Q). A combination of Bcl-2 and YAP inhibitors demonstrated a synergistic effect, substantially reducing tumor area and invasiveness in *Nf2*-KO S1M cells (Fig. 5, O-Q), emphasizing the potential therapeutic value of this combination in NF2 deficient GC. IHC analysis of the proliferation marker Ki-67 and the apoptosis marker cleaved Caspase-3 revealed no difference in cancer cell proliferation or apoptosis in metastatic colonies between the combination-treatment and vehicle-treated groups (Supplemental Figure 14, A–C). Additionally, despite a slight, non-significant reduction in Survivin levels observed in the metastatic foci from verteporfin and combination-treated groups, there were no discernable, statistically significant changes in the expression of Wnt and YAP signaling target genes such as Cyclin D1 and Survivin following treatment (Supplemental Figure 14, D–F). Consistent with the above mentioned findings, the lack of observed differences could be attributed to the small population of CSCs present within secondary metastatic foci, where most differentiated cancer cells show diminished YAP and Wnt activity (Supplemental Figure 9, A and B). Therefore, any alterations in target gene expression in response to drug treatment might be masked in these secondary metastatic tumors. Collectively, these results indicate that the upregulated expression of Bcl-2 because of *Nf2* loss, which leads to increased Wnt signaling, can confer CSCs with resistance to YAP inhibition. Moreover, these results suggest that the combined inhibition of Bcl-2 and YAP signaling can induce synthetic lethality in the context of NF2 deficiency.

### *RASA1 deficiency amplifies the Wnt pathway *via* Bcl-xL, contributing to cancer stemness*

**Fig. 6:**
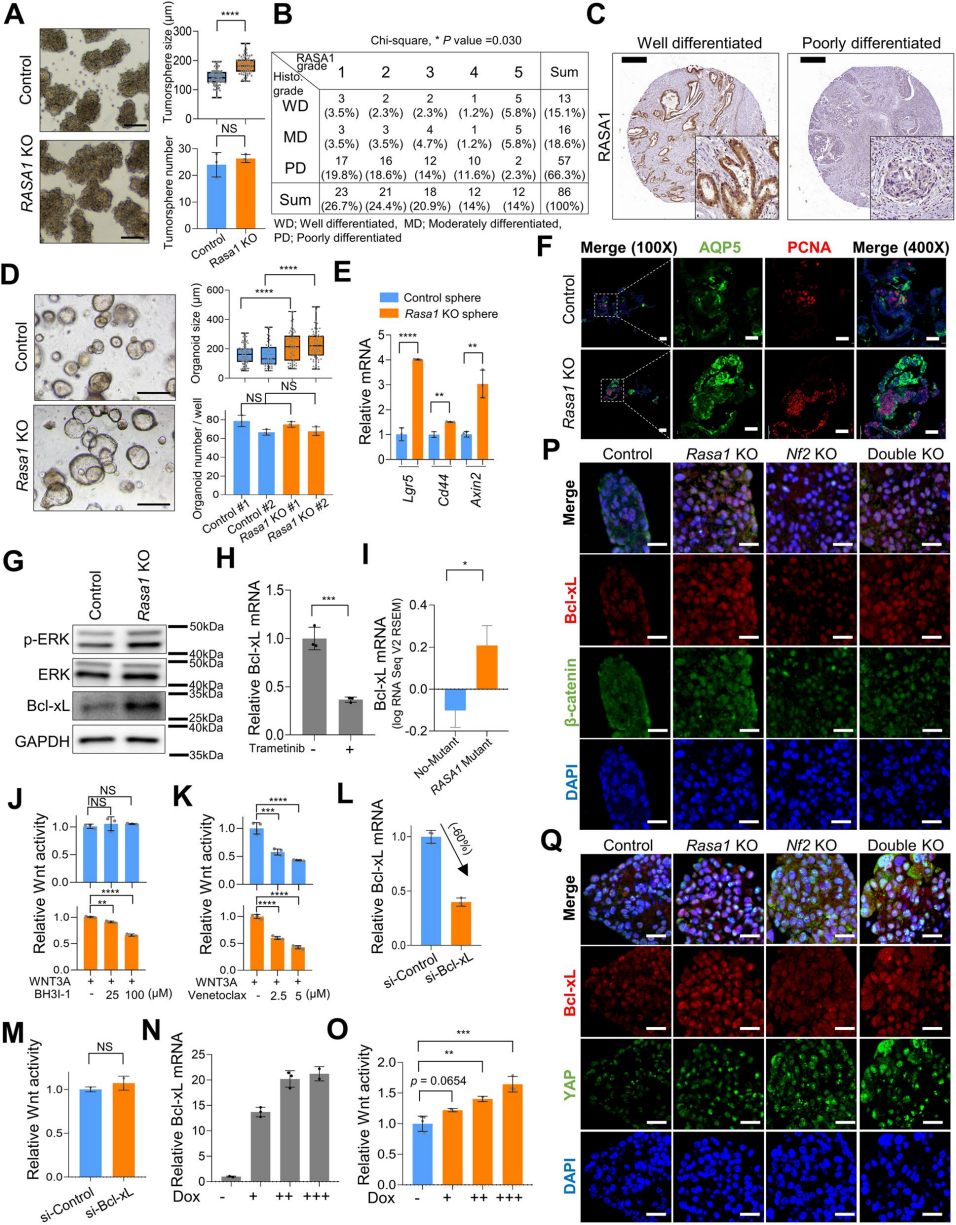
Effects of RASA1 deficiency on stemness via Bcl-xL. **A** (left) Representative images of tumorspheres of control (top) and *RASA1*-KO (bottom) SNU-484 cells. (right) Tumorsphere size (top) and counts (bottom). Bar = 200 µm. **B** Low RASA1 expression has correlation with high histological grade human gastric cancer (GC) in tissue microarray (TMA) tissue. *P* value, chi-square test. **C** Representative RASA1 immunohistochemical images of human GC TMA according to tumor cell differentiation. **D** Representative images of *Tp53*-KO mouse stomach organoids of control and *Rasa1*-KO. (top) Diameter of organoids and (bottom) number of organoids per well. Bar = 200 µm. **E** Relative mRNA expression levels of stem cell markers (*Lgr5*, *Cd44*, and *Axin2*) in control and *Rasa1*-KO mouse stomach organoids. **F** Representative immunofluorescence staining images for AQP5 (green), PCNA (red), and DAPI in control and *Rasa1*-KO Tp53-KO organoids represented in (**D**). Bar = 50 µm. **G** Western blot analysis of p-ERK, ERK, and Bcl-xL in control and *Rasa1*-KO S1M cells. **H** Bcl-xL mRNA expression in S1M cells treated with trametinib (60 nM) for 48 h. **I** Bcl-xL mRNA expression levels depending on *RASA1* mutation status in TCGA GC dataset. **J** and **K** TOP-Flash luciferase reporter assay measuring Wnt pathway activity in control and *Rasa1*-KO S1M cells treated with (**J**) BH3I-1 and (**K**) venetoclax at the indicated concentrations for 24 h. **L** RT-qPCR analysis to confirm knockdown (KD) efficiency of Bcl-xL in S1M cells treated with si-Bcl-xL. **M** TOP-Flash luciferase reporter assay in control and Bcl-xL KD S1M cells. **N** RT-qPCR analysis measuring tetracycline induced Bcl-xL expression in S1M cells after doxycycline (Dox) treatment at multiple doses. **O** TOP-Flash luciferase reporter assay in tetracycline-induced Bcl-xL-overexpression S1M cells. **P** and **Q** Representative immunofluorescence images displaying Bcl-xL (red) paired with active-β-catenin (green) (**P**) and Bcl-xL (red) paired with YAP (green) (**Q**) across control, *Rasa1*-KO, *Nf2*-KO, and *Rasa1*/*Nf2*-double-KO S1 tumorspheres. Bar = 25 µm. Student's t-test was used for statistical analysis, unless otherwise specified

We further investigated the implications of *RASA1* deficiency in human GC tissues and cells. As observed in mouse GC cells, *RASA1*-KO increased the size of tumorspheres in human GC SNU-484 cells (Fig. 6A). Using TMA analyses of human GC tissues, we found that histologically high-grade GC had significantly lower RASA1 expression levels than did low-grade tumor cells (Fig. 6, B and C), suggesting that *RASA1* loss is associated with loss of tumor differentiation. Given the well-established role of the canonical Wnt pathway in supporting normal and transformed gastric stem cells [35, 36, 42], we investigated whether the loss of *Rasa1* resulted in aberrant Wnt signaling in non-transformed gastric epithelial cells. We evaluated the effects of *Rasa1* loss in untransformed gastric epithelial cells by deleting *Rasa1* in *p53*-KO mouse stomach organoids. Three days after single-cell dissociation and seeding in Matrigel, *Rasa1*-KO cells formed larger organoids than did control cells (Fig. 6D). This suggests that *Rasa1*-KO could potentially affect the self-renewal capacity of organoids. Consistent with this, *Rasa1*-KO organoids showed significantly higher expression of Wnt-dependent stem cell markers (Fig. 6E). AQP5 is a recently described gastric stem cell marker that is restricted to LGR5^+^ stem cells in the stomach antrum [42]. AQP5^+^ cells were detected using immunofluorescence staining and were more frequently stained for PCNA than were AQP5^−^ cells (Fig. 6F). AQP staining was stronger in *Rasa1*-KO organoids (Fig. 6F). The data indicate that *Rasa1*-KO promotes the self-renewal and survival of both non-transformed and cancerous gastric epithelial cells.

In deciphering the mechanism by which *Rasa1* loss strengthens Wnt signaling, we considered that RASA1 is a negative regulator of the RAS/MAPK pathway [26]. In our previous research, we discovered that Bcl-xL, induced by MAPK activation, triggers Wnt signaling activation and mediates CSC activity in gastrointestinal malignancies [9, 14]. We hypothesized that MAPK activation resulting from *Rasa1* deficiency could upregulate Bcl-xL and consequently boost cancer stemness. We found that *Rasa1*-KO in S1M cells led to increased ERK phosphorylation and higher Bcl-xL protein expression as compared with levels in control cells (Fig. 6G). To determine whether upregulation of Bcl-xL following *Rasa1* loss was mediated by MAPK activation, we treated S1M cells with trametinib, a potent MEK1/2 inhibitor. Trametinib reduced basal Bcl-xL expression (Fig. 6H), indicating that the MAPK pathway was involved in the maintenance of steady-state Bcl-xL expression. From a clinical perspective, we observed that GC tissues with *RASA1* mutations from TCGA exhibited higher Bcl-xL expression than did *RASA1* wild-type GC tissues (Fig. 6I).

To determine whether the elevated Wnt signaling activity and WNT3A sensitivity observed in *Rasa1*-KO cells (Fig. 3P) was mediated by Bcl-xL, we treated cells with BH3I-1, a Bcl-xL inhibitor. BH3I-1 treatment did not affect basal Wnt signaling activity in control cells, but specifically blocked the enhancement of Wnt signaling caused by *Rasa1*-KO (Fig. 6J). In contrast, treatment with venetoclax, a Bcl-2 inhibitor, led to an overall decrease in Wnt signaling in both control and *Rasa1*-KO cells (Fig. 6K), suggesting that enhancement of Wnt signaling activity due to *Rasa1* loss was specifically mediated by Bcl-xL and not by Bcl-2. As with chemical inhibition of Bcl-xL, knockdown of Bcl-xL did not affect basal Wnt signaling activity (Fig. 6, L and M), whereas exogenous induction of Bcl-xL enhanced Wnt signaling activity in response to WNT3A (Fig. 6, N and O). IF analysis indicated that *Rasa1*-KO and double-KO S1 tumorspheres exhibited elevated levels of Bcl-xL, which co-localized with active-β-catenin, in comparison to both control and *Nf2*-KO tumorspheres (Fig. 6, P and Q). Similarly, *Rasa1*-KO and double-KO metastatic foci showed heightened expression of Bcl-xL compared to their *Nf2*-KO and control counterparts (Supplemental Figure 13, D and E). Collectively, we concluded that *Rasa1* loss augments Wnt signaling and enhances GC stemness through Bcl-xL.

### Rasa1 mutation drives vulnerability to Bcl-xL inhibition

**Fig. 7:**
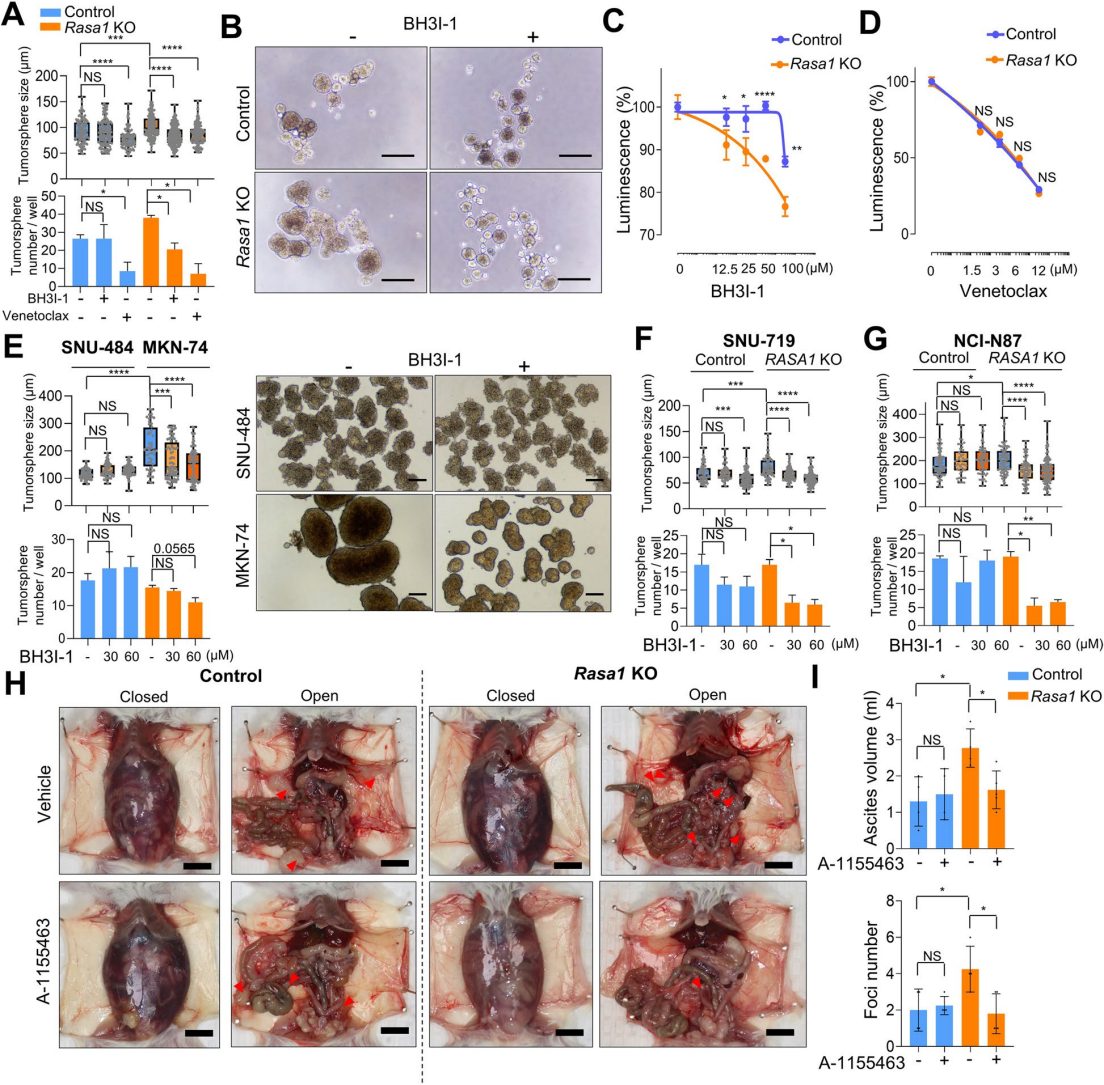
Therapeutic susceptibility of Rasa1-deficient gastric cancer (GC) to Bcl-xL inhibition. **A** and **B** Sphere-forming assay of control and *Rasa1*-KO S1 cells treated with BH3I-1 (25 µM) and/or venetoclax (5 µM). **A** Size and number of tumorspheres and (**B**) representative images of control and *Rasa1*-KO tumorspheres after BH3I-1 treatment. *P* value, Student’s T test. **C** and **D** Dose–response curve of relative cell viability after 5 days of treatment with BH3I-1 (**C**) and venetoclax (**D**) at the indicated concentrations in control and *Rasa1*-KO S1 tumorspheres using ATP cell viability luciferase assay. *P* value, Student’s T test. **E** Sphere-forming assay using SNU-484 and MKN-74 human GC cells treated with BH3I-1 at the indicated concentrations for 5 days. (left) Tumorsphere size and number and (right) representative images of SNU-484 (top) and MKN-74 (bottom) tumorspheres treated with vehicle and BH3I-1. *P* value, Student’s T test. **F** and **G** Sphere-forming assay using control and *Rasa1*-KO SNU-719 (**F**) and NCI-N87 (**G**) cells treated with BH3I-1 at the indicated concentrations for 5 days. The graph indicates the size (top) and number (bottom) of tumorspheres. *P* value, Student’s T test. **H** and **I** Effect of Bcl-xL inhibition on peritoneal dissemination was evaluated by intraperitoneally injecting control or *Rasa1*-KO S1M cells into NOD-SCID mice and treated with vehicle and A-1155463 (5 mg/kg, i.p., once daily) for 10 consecutive days. **H** Representative images of closed and opened peritoneum for each group. **I** Ascites volume (top) and number of macro-metastatic foci in each group (bottom) at the time of necropsy. *P* value, Student’s T test

Based on the finding that Bcl-xL inhibition specifically counteracts the increased Wnt activation in *Rasa1*-KO cells, we hypothesized that *RASA1*-deficient GC would be highly sensitive to Bcl-xL inhibitors. We first investigated the effects of BH3I-1 on tumorsphere formation in *Rasa1*-KO cells. Treatment with BH3I-1 successfully blocked the enhancement of S1M tumorsphere growth induced by *Rasa1-*KO at a concentration that did not affect the growth of control tumorspheres (Fig. 7, A and B). Using an ATP cell viability assay, we found that *Rasa1*-KO S1M cells were more sensitive to BH3I-1 than were controls (Fig. 7C). In contrast, venetoclax treatment reduced tumorsphere size in both control and *Rasa1-*KO cells (Fig. 7A), with no difference in response between the two groups (Fig. 7D).

We next assessed the sensitivity of *RASA1*-mutated human GC cells to BH3I-1 using tumorsphere formation assays. We found that MKN-74, a human GC cell line, harbors a *RASA1* truncating mutation [43]. MKN-74 cells were more sensitive to BH3I-1 than was the *RASA1* wild-type human GC cell line SNU-484 (Fig. 7E). Additionally, *RASA1-*KO increased tumorsphere size and induced sensitivity to BH3I-1 in *RASA1*-wild GC cell lines, including SNU-719 (Fig. 7F) and NCI-N87 (Fig. 7G).

The ability of Bcl-xL inhibitors to inhibit metastasis of *Rasa1*-KO S1M cells in vivo was investigated. In a peritoneal dissemination model using NOD-SCID mice, *Rasa1*-KO S1M cells generated more metastatic foci and bloody ascites in the peritoneal space than did control cells (Fig. 7, H and I). A-1155463 is a Bcl-xL inhibitor validated for in vivo treatment [44]. *Rasa1*-KO S1M cells were sensitive to A-1155463 for inhibition of peritoneal dissemination, whereas control S1M cells showed little effect of Bcl-xL inhibition on metastasis as assessed based on ascites volume and macrofoci in the peritoneal space (Fig. 7, H and I). Collectively, the data provide functional evidence that Bcl-xL inhibition acts more selectively to suppress GC stemness induced by *Rasa1* mutations, leading to inhibition of metastasis. Importantly, these findings suggest a therapeutic vulnerability to Bcl-xL inhibition in *RASA1*-mutated metastatic GC.

### Bcl-xL and YAP signaling regulate synthetic lethality in RASA1 and NF2 co-deficiency

**Fig. 8:**
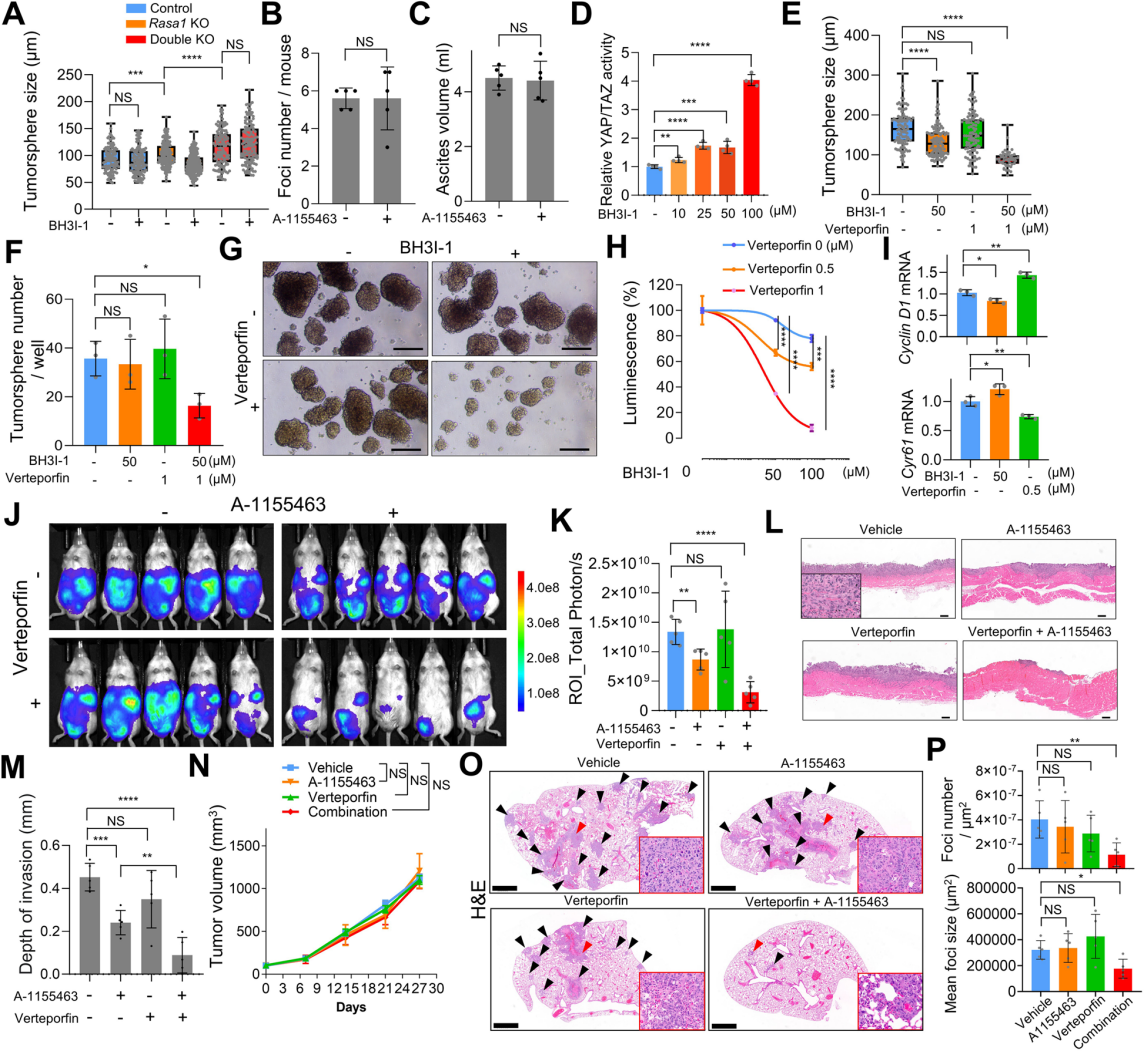
Synthetic lethality regulated by Bcl-xL and YAP signaling. **A** Sphere-forming assay using control, *Rasa1*-KO, and *Rasa1*/*Nf2-*double-KO S1 cells treated with BH3I-1 (25 µM) for 5 days. **B** and **C** Effect of Bcl-xL inhibition on peritoneal dissemination model of *Rasa1*/*Nf2-*double-KO S1M cells in NOD-SCID mice treated with vehicle (*n* = 5) or A-1155463 (*n* = 5, 7.5 mg/kg, i.p., once daily) for 10 consecutive days. **B** Ascites volume and (**C**) macro-metastatic foci numbers measured at necropsy. **D** HOP-Flash luciferase reporter assay in BH3I-1-treated S1 cells after 48 h. **E**, **F**, and **G** Sphere-forming assay of *Rasa1*/*Nf2-*double-KO S1 cells treated with BH3I-1 (50 µM) and/or verteporfin (1 µM) for 5 days. **E** Tumorsphere size, (**F**) number, and (**G**) Representative images of tumorspheres. **H** Luciferase ATP cell viability assay of *Rasa1*/*Nf2-*double-KO S1 tumorspheres, treated with BH3-I and/or verteporfin for 5 days at indicated doses. **I** RT-qPCR analysis of mRNA expression levels of (top) Wnt-dependent transcription (*Ccnd1*) and (bottom) YAP-dependent transcription (*Cyr61*) depending on BH3-I and/or verteporfin treatment in *Rasa1*/*Nf2-*double-KO S1 tumorspheres. **J**-**M** (**J**) Bioluminescence in vivo imaging of NOD-SCID mice 8 days after intraperitoneal transplantation of in *Rasa1*/*Nf2-*double-KO S1M cells, treated with vehicle (*n* = 5), A-1155463 (*n* = 5, 7.5 mg/kg, i.p., once daily), verteporfin (*n* = 5, 10 mg/kg, i.p., every other day), or combination of both drugs (*n* = 5). **K** Total bioluminescence signals in (**J**). **L** Representative H&E-stained images of peritoneal metastatic foci. Bar = 200 μm. **M** Depth of invasion was measured based on H&E slides. **N**, **O**, and **P** (**N**) Subcutaneous tumor volume of *Rasa1*/*Nf2-*double-KO S1M cells treated with vehicle (*n* = 5), A-1155463 (*n* = 5, 7.5 mg/kg, i.p., once daily), verteporfin (*n* = 5, 10 mg/kg, i.p., every other day) or combination of both drugs (*n* = 5). **O** Representative histopathological images of pulmonary metastasis. Black arrows indicate metastatic foci. Red boxes represent magnified views of red arrow locations. Bar = 1 mm (**P**) Statistical analysis of pulmonary metastasis regarding the number of metastatic foci (top) and mean foci size (bottom). Student's t-test was used for statistical analysis, unless otherwise specified

Interestingly, *Nf2*/*Rasa1*-double-KO S1M cells did not show a specific reduction in tumorsphere size in response to BH3I-1 treatment as seen in *Rasa1*-KO cells (Fig. 8A). At a concentration of A-1155463 shown to be effective in *Rasa1*-KO cells (Fig. 7H), the compound failed to reduce peritoneal dissemination of the double-KO cells (Fig. 8, B and C). The results indicate that hypersensitivity to Bcl-xL inhibition in *Rasa1*-mutated cells was attenuated by NF2 deficiency. Our previous observations indicated crosstalk between Wnt and YAP signals in CSCs, a consequence of the loss of NF2 and RASA1. Therefore, we hypothesized that the reduced sensitivity to Bcl-xL inhibition in *Nf2*-KO cells could be attributed to YAP activation. Indeed, treating cells with BH3I-1 led to a dose-dependent increase in YAP activity (Fig. 8D). Therefore, we postulated that double-KO cells showing high YAP signaling might exhibit resistance to Bcl-xL inhibitor, and inhibiting YAP could enhance the therapeutic effects of BH3I-1. To test this, we performed tumorsphere assays with BH3I-1 alone or in combination with verteporfin in *Nf2*/*Rasa1*-double-KO cells. We treated cells with a higher concentration of BH3I-1 to enhance the effect, as the 25 µM concentration did not affect tumorsphere formation in double-KO cells. Combined treatment with BH3I-1 and verteporfin synergistically reduced the size and number of tumorspheres (Fig. 8, E, F and G). Additionally, the ATP cell viability assay showed that verteporfin treatment synergistically improved the response to BH3I-1 in *Nf2*/*Rasa1*-double-KO cells (Fig. 8H). As anticipated, RT-qPCR results showed decreased expression of the Wnt target gene *Ccnd1* and increased expression of the YAP target gene *Cyr61* upon BH3I-1 treatment, while verteporfin treatment resulted in an increase in *Ccnd1* and a decrease in *Cyr61* expression (Fig. 8I). The findings suggest that YAP activation due to NF2 loss mediates resistance to Bcl-xL inhibitors in *Rasa1* deficient cells and highlights the potential of combining YAP and Bcl-xL inhibitors for treatment of highly metastatic GC.

To validate the finding that *Rasa1* loss drives susceptibility to Bcl-xL inhibition and the synergistic effect of combined treatment with Bcl-xL and YAP inhibitors through in vivo models, we transplanted 5 × 10^6^ *Rasa1*/*Nf2-*double-KO S1M cells labeled with luciferase into NOD-SCID mice. We treated mice with a higher dose of 7.5 mg/kg A-1155463 to enhance the effect, as 5 mg/kg did not show a metastasis-suppressive effect on double-KO cells. Seven days after injection, verteporfin single treatment did not give rise to a significant effect, whereas A-1155463 showed a mild effect in suppressing peritoneal dissemination, as assessed using Luciferase bioluminescence and examination of the peritoneal dissemination area (Fig. 8, J and K). However, combined inhibition of Bcl-xL and YAP synergistically reduced peritoneal dissemination in *Rasa1*/*Nf2-*double-KO cells (Fig. 8, J-M). The combination treatment significantly reduced the invasiveness of peritoneal seeding, as assessed using H&E staining (Fig. 8, L and M).

IHC analysis revealed no difference in cancer cell proliferation and apoptosis in metastatic colonies between combination-treatment and vehicle-treated groups (Supplemental Figure 15, A–C). Additionally, even though we observed a slight, non-significant decrease in Survivin levels in the metastatic foci from verteporfin and combination-treated groups, we did not detect any substantial changes in the expression of Wnt and YAP signaling target genes, Cyclin D1 and Survivin, post treatment (Supplemental Figure 15, D–F). As previously discussed, the absence of significant variations may be attributed to the small population of CSCs in secondary metastatic foci, where the majority of differentiated cancer cells exhibited low YAP and Wnt activity. To validate our hypothesis, we employed a subcutaneous transplantation model using double-KO S1M cells, which metastasize naturally to the lungs. Although the combination treatment did not significantly decrease the growth of subcutaneous tumors, it substantially reduced lung metastases (Fig. 8N–P). These findings support the notion that combined YAP and Bcl-xL inhibition might suppress the metastatic process primarily by targeting CSCs. Collectively, the in vivo findings suggest that combined inhibition of Bcl-xL and YAP could offer an effective therapeutic approach for treating highly metastatic GC with *Nf2* and *Rasa1* deficiency.

## Discussion

**Fig. 9:**
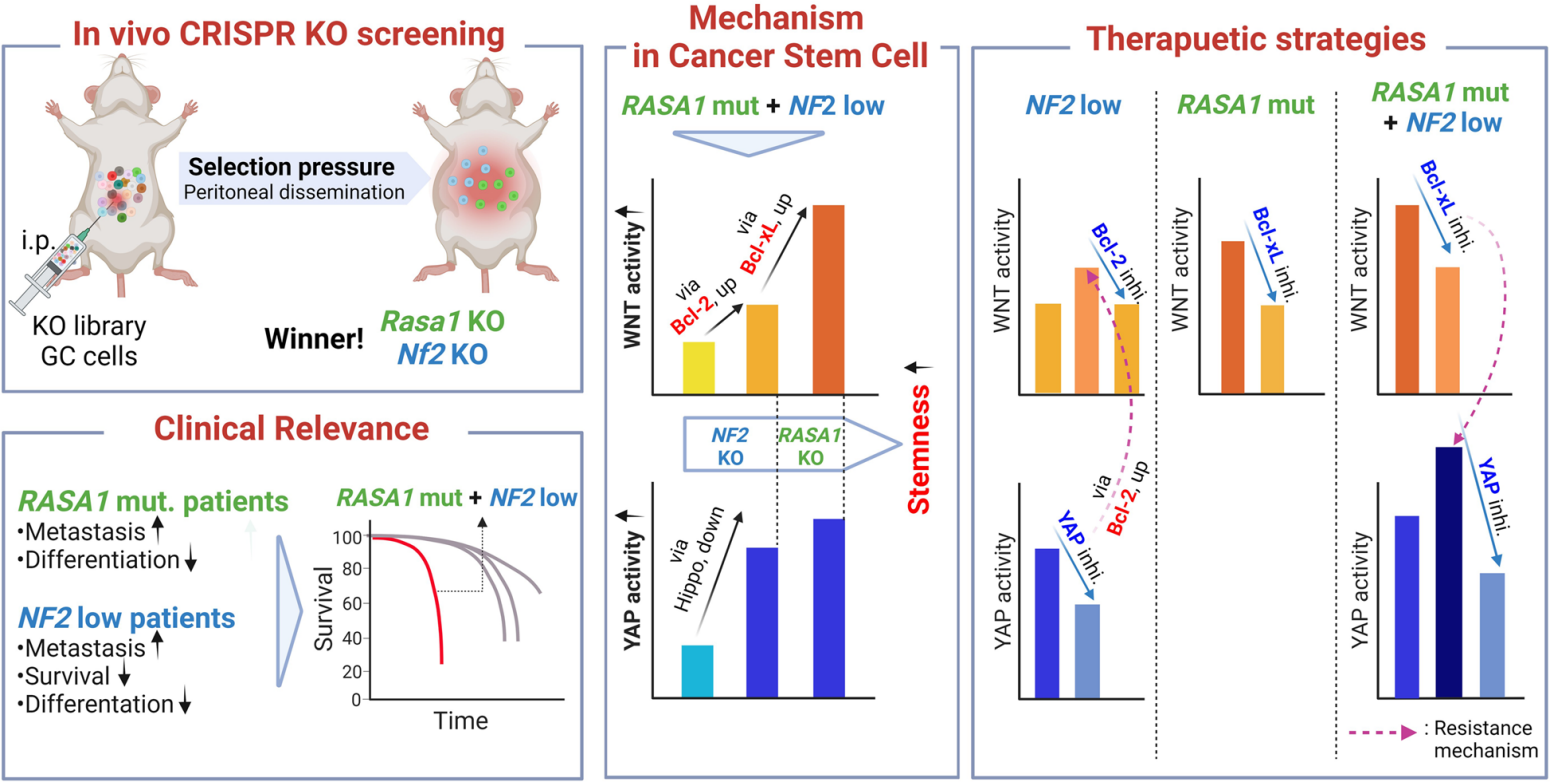
Schematic representation of the methodology, clinical relevance, underlying mechanisms, and therapeutic implications presented in this study. We uncovered intricate interactions between Wnt and YAP signaling, regulated by RASA1 and NF2 deficiency in CSC biology. Our findings spotlight a novel therapeutic strategy: the inhibition of Bcl-2 family members and YAP signaling, potentially inducing synthetic lethality, particularly in NF2 and RASA1 deficient cases. This strategy opens up a potential path to counter drug resistance in highly metastatic GC

Drug resistance, metastasis, and recurrence, primarily driven by CSCs, are the major causes of cancer mortality [31, 39]. Therefore, unraveling CSC-specific signaling mechanisms and characteristics is of paramount clinical importance for the advancement of targeted anticancer treatments. In this study, we employed unbiased genome-wide CRISPR/Cas9 KO screening with an in vivo mouse ChetPS GC model, using peritoneal seeding and identified *Rasa1* and *Nf2* as critical metastasis-suppressing genes. Our study revealed novel roles for deficiency of these genes in promoting gastric cancer stemness and metastasis through upregulation of Bcl-2 family members, thereby enriching our understanding of the complex interactions between Wnt and YAP signaling in CSC biology (Fig. 9). Although ample evidence emphasizes the significance of Wnt and YAP signaling in maintaining CSCs and advancing cancer progression [37], the CSC-specific regulatory mechanisms of these signaling pathways remain unclear. As therapeutic targeting of these CSCs is a subject of intense investigation, a precise understanding of the relationship between YAP and Wnt activity in the context of CSC traits is of utmost importance. Our research elucidates the complex crosstalk between YAP and Wnt signaling pathways in CSC biology, which could potentially be exploited under NF2 and RASA1 deficiency. Despite the upregulation of secreted Wnt inhibitors due to YAP activation in NF2 deficiency, we observed a paradoxical enhancement of Wnt signaling, underscoring the aberrant interaction within CSCs under NF2 deficiency. In therapeutic settings, we propose that resistance to YAP inhibition in NF2 deficiency may stem from increased Wnt signaling, amplified by Bcl-2 upregulation or by antagonism induced by YAP signaling. The intricacy of this crosstalk is also evident in the context of *RASA1* mutations, wherein inhibition of Bcl-xL reduced Wnt signaling and concomitantly increased YAP signaling. Although the exact mechanisms by which Bcl-xL inhibition suppresses Wnt signaling and elevates YAP signaling warrant further investigation, we suggest that this could represent a compensatory mechanism employed by CSCs to preserve stemness and promote drug resistance during therapeutic treatments. Our findings underscore the pivotal role of Bcl-2 family members as key signaling molecules in maintaining this compensatory relationship between Wnt and YAP signaling, thereby safeguarding cancer stemness and enabling therapeutic resistance. Consequently, this insight offers a novel therapeutic strategy: inducing synthetic lethality by inhibiting Bcl-2 family members and YAP signaling, offering a potential avenue for overcoming drug resistance in highly metastatic GC, particularly in cases with NF2 and RASA1 deficiencies. This strategy exemplifies the promise of personalized medicine for treating advanced GC, based on specific molecular features.

In this study, we proposed that *RASA1* mutation and *NF2* deficiency can define molecular subtypes of metastatic GC. We found that *RASA1* mutations are frequently present in patients with GC and correlate with distant metastasis. *RASA1* deletion in both human and mouse GC cells promoted metastasis, suggesting a functional role in GC metastasis. Patients with *RASA1* mutations were considered as potential subjects for therapeutic interventions. Conversely, although *NF2* mutations are frequently observed in spontaneous schwannomas and meningiomas, these mutations occur less frequently in other sporadic solid tumors, such as breast and colorectal cancers [22]. In GC, the effect of *NF2* mutation or inactivation has not been extensively studied, partly because *NF2* driver gene mutations resulting in merlin inactivation are relatively rare in this cancer type. Nevertheless, *NF2* deficiency may play a critical role in GC pathogenesis as NF2 suppresses tumorigenesis by activating upstream components of the Hippo pathway [45] and Hippo pathway signaling is often dysregulated in GC [5, 46], *NF2* deficiency may play a critical role in GC pathogenesis. In this study, we found that *NF2* deficiency has a prognostic value, as patients with GC who exhibit low *NF2* expression experience higher metastasis rates and shorter survival times. Incorporating NF2 status into risk assessment models may enable better prognostication and guide treatment decisions for patients with metastatic GC, particularly those with *RASA1* mutations. Additionally, *NF2* deletion in both human and mouse GC cells promoted metastasis, further suggesting a functional role in GC metastasis. This suggests that an *NF2-*low status could be a useful parameter for risk classification of metastatic GC.

Our findings indicate that an interaction between YAP and Bcl-2 family members can lead to synthetic lethality that would aid in overcoming drug resistance depending on the *RASA1* and *NF2* status. First, we discovered that RASA1-mutated GC is particularly vulnerable to Bcl-xL inhibition. In this study, we revealed the functional importance of *RASA1* mutations in GC metastasis, and identified a novel mechanism by which *RASA1* mutations amplify Wnt activity, boosting CSC fitness by increasing Bcl-xL levels. Although previous clinical evidence has linked Rasa1 loss to cancer metastasis, most functional studies have focused on primary tumor growth and progression through the enhancement of RAS-ERK signal amplification. Second, we observed that *NF2*-low GC exhibited significant suppression of metastasis in response to combined inhibition of YAP and Bcl-2. *NF2* deficiency paradoxically led to resistance to YAP inhibitors. We revealed that resistance to YAP inhibition is acquired through Bcl-2-mediated activation of Wnt signaling, indicating that synthetic lethality drug resistance is regulated by Bcl-2 and YAP signaling. Although NF2 loss is known to contribute to tumor metastasis [47], no direct strategy has been developed to overcome NF2 deficiency during cancer progression. Third, we demonstrated that *RASA1*-mutated and *NF2*-low GC exhibits a synergistic metastasis-suppressing effect in response to combined YAP and Bcl-xL inhibition. Resistance to Bcl-xL inhibition in *RASA1*-mutated GC with low *NF2* expression can be acquired through activation of YAP signaling caused by *NF2* deficiency. Based on these findings, further research is needed to understand how Bcl-xL inhibition specifically upregulates YAP inhibition as an alternative pathway for maintaining cancer stemness.

An aberrant Wnt/β-catenin signaling pathway facilitates CSC renewal and fitness [34, 48, 49]. Suppressing this pathway could offer therapeutic options for various cancer types, and targeting Wnt signaling in preclinical GC models has shown efficacy [36]. However, developing Wnt/β-catenin-targeted therapies faces challenges such as off-target effects, unintended side effects due to the involvement of the pathway in normal tissue functions, and limited druggability for targeting nuclear β-catenin [50]. Additionally, the regulation and dynamics of Wnt signaling are context-dependent and influenced by cell type, tumor microenvironment, and interactions with various signaling pathways involved in cancer progression [50, 51]. Consequently, blocking Wnt signaling by targeting individual components is likely to be limited owing to the complexity and variation across contexts, warranting further investigation into Wnt regulation in CSC biology. This study demonstrated that Bcl-2 family members play crucial roles in CSC fitness by regulating Wnt signaling. Bcl-2 family members play non-canonical roles in tumor progression beyond a classical role in inhibiting apoptosis, through interactions with proteins outside the Bcl-2 protein family [15]. Our findings revealed novel roles for Bcl-2 family members in controlling Wnt signaling in CSCs, with Bcl-2 and Bcl-xL playing roles in maintaining and amplifying Wnt signaling, supporting cancer stemness and metastasis. Bcl-2 inhibition significantly reduced basal levels of Wnt signaling in control cells, as well as NF2-KO-induced Wnt signaling, indicating that Bcl-2 fundamentally maintains Wnt signaling to support CSCs. Conversely, Bcl-xL inhibition did not affect basal Wnt signaling activity, but selectively reduced amplified Wnt signaling caused by *RASA1-*KO. The results demonstrate the reciprocal roles of Bcl-xL and Bcl-2, induced by loss of Rasa1 and Nf2, respectively, in regulating Wnt signaling in CSCs. However, the exact mechanisms underlying this relationship remain unclear. Further research is needed to elucidate how Bcl-2 family members regulate Wnt signaling and to identify partner molecules involved in this process.

Our study unveiled molecular mechanisms underlying GC metastasis driven by *RASA1* mutations and *Nf2* deficiency and proposed a novel therapeutic strategy involving combined inhibition of Bcl-2 family members and YAP for treatment of highly metastatic GC. The findings provide a strong basis for further research to determine the clinical relevance and applicability of this approach. Over the years, Bcl-2 family inhibitors have been developed and venetoclax has been successfully used in the treatment of hematological malignancies. However, its efficacy is limited in primary solid cancers [19]. The Hippo pathway target, YAP, is crucial for promoting metastasis through the TEAD-interaction domain [52]; however, pharmacological blockade of TEAD-YAP has therapeutic limitations in cancer cells [53]. Emerging evidence suggests that deregulation of YAP/TAZ signaling may be a major mechanism underlying intrinsic and acquired resistance to various targeted therapies [54]. Notably, our study pioneers novel advancements by expanding the potential use of Bcl-2 family and YAP inhibitors in addressing synthetic lethality that would aid in overcoming drug resistance in GC metastasis. The primary innovation of our work lies in elucidating the novel effects of RASA1 and NF2 deficiency in amplifying GC stemness. This amplification is facilitated through the modulation of YAP and Wnt signaling interactions by Bcl-2 family members, particularly at CSC-specific nodes. This breakthrough accentuates the importance of considering GC molecular phenotypes while formulating targeted therapies, emphasizing the potential of personalized medicine in tackling this formidable disease. By providing insights into the distinctive molecular interactions in *RASA1*-mutated and NF2-deficient GC, our work paves the way for devising more precise and effective treatment strategies for this condition.

### Supplementary Information

**Additional file 1:** Materials and methods.

 

**Additional file 2: Supplemental Table 1.** Correlation between RASA1 immunoreactivity and clinicopathological parame ters in human GC tissues. *P*-value, Chi squared test.

**Additional file 3: Supplemental Table 2.** Correlation between NF2 immunoreactivity and clinicopathological parameters in human GC tissues. *P*-value, Chi squared test.

**Additional file 4: Supplemental Table 3.** Information of drugs and chemicals used in this study.

**Additional file 5: Supplemental Table 4.** Primer sequences for qPCR and RT-qPCR.

**Additional file 6: Supplemental Figure 1.** Establishment of the three-dimensional tumorsphere culture system.

**Additional file 7: Supplemental Figure 2.** In vivo validation of target genes using the peritoneal dissemination model.

**Additional file 8: Supplemental Figure 3.** In vivo validation of target genes using the spleno-hepatic metastasis model.

**Additional file 9: Supplemental Figure 4.** Effect of NF2 and RASA1 deficiency on metastasis in human GC.

**Additional file 10: Supplemental Figure 5.** The effects of NF2 and RASA1 deficiency on monolayer growth.

**Additional file 11: Supplemental Figure 6.** Analysis of RNA sequencing data from *Rasa1-* and *Nf2*-KO S1 Tumorspheres.

**Additional file 12: Supplemental Figure 7.** Western blot analysis for YAP signal activation resulting from NF2 deficiency.

**Additional file 13: Supplemental Figure 8.** NF2 deficiency induces anoikis resistance via YAP activation.

**Additional file 14: Supplemental Figure 9.** Evaluation of Wnt and YAP signaling in *Nf2*- and *Rasa1*-KO peritoneal dissemination models.

**Additional file 15: Supplemental Figure 10.** Examination of lung metastasis in a subcutaneous transplantation model using *Rasa1*-KO S1M cells.

**Additional file 16: Supplemental Figure 11.** Immunohistochemical analysis of NF2 and β-catenin in human GC tissues.

**Additional file 17: Supplemental Figure 12.** Analysis of Wnt inhibitor expression in response to *Nf2*-KO and YAP/TAZ signaling modulation.

**Additional file 18: Supplemental Figure 13.** Immunofluorescence staining analysis for Bcl-2, Bcl-xL, β-catenin, and Yap1 in control, *Rasa1-*KO, *Nf2*-KO, and *Rasa1/Nf2*-double-KO peritoneal metastatic foci.

**Additional file 19: Supplemental Figure 14.** Immunohistochemical analysis following YAP and Bcl-2 inhibitor treatment in *Nf2/Rasa1*-double KO S1M peritoneal dissemination model.

**Additional file 20: Supplemental Figure 15.** Immunohistochemical analysis following YAP and Bcl-xL inhibitor treatment in *Nf2/Rasa1*-double KO S1M peritoneal dissemination model.

## Data Availability

The results shown here are in whole or part based upon data generated by the TCGA Research Network: https://www.cancer.gov/tcga. RNA sequencing data are deposited in GEO (https://www.ncbi.nlm.nih.gov/geo/) and are publicly available as of the date of publication under the accession GSE239457. The datasets used and/or analyzed during the current study are available from the corresponding author on reasonable request.

## Abbreviations

AQP5: Aquaporin 5
BCL-2: BCL2 apoptosis regulator
BCL-XL: BCL2-like 1
BMP4: Bone morphogenetic protein 4
CCND1: Cyclin D1
CD44: Cluster of differentiation 44
CDH1: Cadherin 1
c-Myc: MYC proto-oncogene
CRISPR: Clustered regularly interspaced short palindromic repeats
CSC: Cancer stem cell
CTGF: Connective tissue growth factor
CYR61: Cysteine rich angiogenic inducer 61
ERK: Extracellular signal-regulated kinases
GAPDH: Glyceraldehyde-3-phosphate dehydrogenase
GC: Gastric cancer
gDNA: Genomic DNA
GO: Gene ontology
H&E: Hematoxylin and eosin stain
i.p.: Intraperitoneal
i.v.: Intravenous
IF: Immunofluorescence
IGFBP4: Insulin like growth factor binding protein 4
IHC: Immunohistochemistry
KEGG: Kyoto encyclopedia of genes and genomes
KO: Knockout
GSEA: Gene set enrichment analysis
LGR5: Leucine rich repeat containing G protein-coupled receptor 5
NF2: Moesin-ezrin-radixin like (MERLIN) tumor suppressor
NGS: Next generation sequencing
p.o.: Per os
PCA: Principal component analysis
PCNA: Proliferating cell nuclear antigen
qPCR: Quantitative polymerase chain reaction
RASA1: RAS p21 protein activator 1
ROI: Region of interst
sgRNA: Single guide RNA
siRNA: Small interfering RNA
SMAD4: SMAD family member 4
TAZ: WW domain containing transcription regulator 1
TCGA: The Cancer Genome Atlas
TEAD: TEA Domain Transcription Factor
TMA: Tissue microarray
TRP53: Transformation related protein 53
YAP: Yes1 associated transcriptional regulator
