# Supplementary material for: Combined inhibition of Bcl-2 family members and YAP induces synthetic lethality in metastatic gastric cancer with RASA1 and NF2 deficiency
Source: Mol Cancer. 2023 Sep 20;22:156. doi: 10.1186/s12943-023-01857-0 (PMC10510129; doi:10.1186/s12943-023-01857-0)
Supplement: Supplementary file 1 — Additional file 1: Materials and methods. [file 12943_2023_1857_MOESM1_ESM.pdf]

MATERIALS AND METHODS

Next-generation sequencing of amplicon libraries

We used two-step PCR to generate amplicon libraries targeting genetically integrated guide (g)RNA sequences. We performed primary PCR using the primer set LentiCRISPR v2 gRNA scaffold F: 5′-GACAGCAGAGATCCAGTTTGG-3′ and LentiCRISPR v2 gRNA scaffold R: 5′-ATGCTCCAGACTGCCTTGGG-3′ to amplify the gRNA locus and KAPA HiFi HotStart ReadyMix (KK2602, Kapa Biosystems, Wilmington, MA, United States). Primary PCR conditions were 98°C (20 s), 65°C (15 s), and 72°C (15 s) for 23 cycles. Secondary PCR was performed to add adapters and barcodes at 98°C (20 s), 73°C (15 s), and 72°C (15 s) for 24 cycles, using KAPA HiFi HotStart ReadyMix and previously reported primer sets [1]. The amplified library products were purified using a PCR purification kit (28104, Qiagen, Venlo, The Netherlands) and sequenced on a HiSeq 2500 system (150 bp × 2 paired-end; Illumina, Inc, San Diego, CA, USA). We subjected the raw data to adapter trimming with Trimmomatic (v0.38) [2]. The sequences of primer-binding sites were located in the adapter trimmed reads, and we regarded the first 21 bp sequences after the primer-binding sites as the single (s)gRNA of the samples. Consequently, we obtained the primer-binding site–sgRNA structure with a length of 52 bp (33 bp primer-binding site + 19 bp sgRNA). We used MAGeCK (0.5.9.5) to count the sgRNA sequences with the --trim-5 33 --sgrna-len 19 --pdf-report option [3].

Manual Sanger sequencing of amplicon libraries

We amplified the inserted gRNA sequences using the following primers: LentiCRISPR v2 gRNA scaffold F: 5′- GACAGCAGAGATCCAGTTTGG-3′ and LentiCRISPR v2 gRNA scaffold R: 5′-ATGCTCCAGACTGCCTTGGG-3′ as described above. The amplified PCR fragments were gel extracted and TA cloning was performed using Mighty TA-cloning Kit (6028, Takara, Shiga, Japan). After blue/white selection, we only picked white colonies, which contained an amplified gRNA sequence, and extracted plasmids using FavorPrep™ GEL/PCR Purification Kit (FAGCK 001, Favorgen, Ping Tung, Taiwan). We then sequenced cloned fragments using the standard Sanger sequencing method with the M13 RV primer: 5′-CAGGAAACAGCTATGAC-3′.

RNA sequencing analysis

To investigate the transcriptomic differences of KO cells, we analyzed the S1 tumorspheres of control, *Nf2*-KO, *Rasa1*-KO, and double-KO using RNA sequencing. We calculated total RNA concentration using Quant-IT RiboGreen (R11490, Invitrogen, Waltham, MA, USA). To assess the integrity of the total RNA, samples are run on TapeStation RNA ScreenTape (5067-5576, Agilent Technologies, Santa Clara, CA, USA). Only high-quality RNA preparations, with RIN greater than 7.0, were used for RNA library construction.

A library was independently prepared with 0.5 μg of total RNA for each sample using Illumina TruSeq Stranded Total RNA Library Prep Gold Kit (20020599, Illumina, Inc.). The first step in the workflow was removing the rRNA in the total RNA. Subsequently, we fragmented the remaining mRNA using divalent cations under elevated temperature. The cleaved RNA fragments were copied into first-strand complementary (c)DNA using SuperScript II reverse transcriptase (18064014, Invitrogen) and random primers. This was followed by second-strand cDNA synthesis using DNA polymerase I, RNase H and dUTP. These cDNA fragments then underwent an end-repair process, the addition of a single A base, and then ligation of adapters. Next, we purified the products and amplified them using PCR to create the final cDNA library.

The libraries were quantified using KAPA Library Quantification kits for Illumina Sequencing platforms according to the qPCR Quantification Protocol Guide (KK4854, Kapa Biosystems) and were qualified using TapeStation D1000 ScreenTape (5067-5582, Agilent Technologies). Indexed libraries were submitted to an Illumina NovaSeq (Illumina, Inc.), and the paired-end (2×100 bp) sequencing was performed by the Macrogen Inc. (Seoul, South Korea).

Data Processing and Analysis

Paired-end sequencing reads were generated on the Illumina sequencing NovaSeq platform. Before starting analysis, Trimmomatic v0.38 was used to remove adapter sequences and trim bases with poor base quality. The cleaned reads were aligned to the *Mus musculus (mm10)* using HISAT v2.1.0 [4], based on the HISAT and Bowtie2 implementations. The reference genome sequence and gene annotation data were downloaded from NCBI Genome assembly and NCBI RefSeq database respectively. Aligned data (SAM file format) were sorted and indexed using SAMtools v 1.9. After alignment, the transcripts were assembled and quantified using StringTie v2.1.3b [5]. Gene-level and

Transcript-level quantification were calculated as raw read count, FPKM (Fragments Per Kilobase of transcript per Million mapped reads) and TPM (Transcripts Per Million).

Differential Gene Expression Analysis

Statistical analyses of differential gene expression (DGE) were performed by DESeq2 v 1.24.0 [6] using raw counts as input. In the data preprocessing step, genes with one more than zeroed read count values in the samples were excluded. In the data preprocessing step, genes with all zeroed read count values in the samples were excluded. Principal component analysis (PCA) and multidimensional scaling (MDS) plots were generated to confirm the similarity of expression between samples. Filtered data set was applied with relative log expression (RLE) normalization to correct the variation of library sizes among samples. Statistical significance of differential gene expression was determined using DESeq2 nbinom WaldTest [6]. Fold-change and *P*-value were extracted from the result of WaldTest. All *P*-values are adjusted by Benjamini–Hochberg algorithm to control false discovery rate (FDR). Significant gene list was filtered by $|\text{fold change}| \geq 3$ & raw p-value < 0.05. Hierarchical clustering on rlog transformed values for significant genes was performed with these parameters (distance metric =Euclidean distance, linkage method=complete). Gene-enrichment and functional annotation analysis for significant gene list were performed based on gProfiler (https://biit.cs.ut.ee/gprofiler/orth), and Kyoto Encyclopedia of Genes and Genomes (KEGG) pathway database (https://www.genome.jp/kegg/) respectively [7]. All data analysis and visualization of differentially expressed genes was conducted using R 3.6.0 (www.r-project.org).

Plasmids

Cre-recombinase system

The PX459_PGK_Cre plasmid was constructed as previously reported [8]. Briefly, the Cre recombinase-coding region of Lenti-sgNT/Cre35 (66895, Addgene, Watertown, MA, USA) was subcloned into the XbaI and EcoRI sites of the PX459 vector (pSpCas9(BB)-2A-Puro (PX459) V2.0; 62988, Addgene). The constructed Cre-recombinase plasmid was transiently transfected with Lipofectamine® 2000 (11668027, Invitrogen) following the manufacturer’s instructions.

PiggyBac transposon system

The Cas9 cDNA was cloned from pX330 (42230, Addgene) and inserted into a PiggyBac (PB) vector (pPBCAG-IRES-blasticidin resistance-pA; pPBCAG-cHA-IB). Using the same cloning strategy, amplicons were digested with NotI/XhoI, and cloned into the PiggyBac vector digested with NotI/XhoI. We cotransfected this pPB-CAG-Cas9-IB vector with pPyCAG-PBase into gastric cancer cells and stomach organoids using Lipofectamine LTX (15338030, Invitrogen) to constitutively express Cas9 and blasticidin resistance genes. After 1 week of blasticidin (5 μg/ml, ant-bl-1, InvivoGen, San Diego, CA, USA) selection, pooled clones with active Cas9 activity were used for subsequent experiments.

To create in vivo luciferase-expressing cells for in vivo imaging system (IVIS) analysis, the pGL4.10 [luc2] vector (E6651, Promega Corp., Madison, WI, USA) was digested with NotI and XhoI and subcloned into pPBCAG-rtTAM2-IN (124166, Addgene). Following selection with G418 (200 μg/ml; Geneticin, ant-gn-1, InvivoGen) for 1 week, antibiotic-resistant clones were pooled and used for subsequent experiments.

Tetracycline-inducible transgenic overexpression system

For the tetracycline-induced gene expression system, the PB-TET plasmid (20909, Addgene, a gift from Andras Nagy) was used. The PiggyBac plasmids containing the tetracycline-inducible *Bcl-2* (NM_009741) or *Bcl-xL* (NM_009743.4) expression vectors, designated as PB-Tet-*Bcl-2* and PB-Tet-*Bcl-xL*, were constructed by inserting the cDNA of *Bcl-2* and *Bcl-xL*, which were amplified through PCR from total mouse cDNA, between the NotI and XhoI sites of PB-TET. Mouse GC cells were transfected with PB-Tet and PB-PBase using the Lipofectamine LTX Reagent, as described above. To induce the expression of the transgene, various concentrations of doxycycline (D9891, Sigma-Aldrich, St Louis, MO, USA) were added to the culture medium. Upon activation of rtTAM through doxycycline treatment, blasticidin and G418 (Geneticin; 200 μg/ml, ant-gn-1, InvivoGen) selection were performed, and after 1 week of blasticidin and neomycin selection, the antibiotics resistant clones were pooled and used for subsequent experiments.

Lentivirus system

The HEK-293T cell was cultured in 6-well plates, or the materials used in lentiviral packaging were proportionally adjusted to culture surface area.

The HEK-293T cells were cultured in 6-well plates for lentiviral packaging. However, when it was necessary to increase the production of lentivirus, the materials used in lentiviral packaging were proportionally adjusted based on the cell culture surface area (cm^2). For packaging the lentivirus, we used third-generation lentiviral packaging plasmids. To prepare the 6-well plates conditions, 750 ng of pLP1, 750 ng of pLP2, 500 ng of pLP/VSVG, and 1000 ng of lentiviral plasmid were suspended in 500 µl of Opti-MEM™ (31985070, Gibco, Billings, MT, USA). Next, either 12 µl of polyethylenimine (PEI; 1 mg/ml, 26008-5, Polysciences, Warrington, PA, USA) or Lipofectamine™ 2000 (11668027, Invitrogen) was suspended in another 500 µl of Opti-MEM™. The two suspensions were mixed and incubated for 20 min at room temperature (RT; 22 ± 2°C). The PEI/DNA complex was gently dropped into the culture plate and incubated for 18 h, and the medium was changed to Dulbecco's modified Eagle's medium (DMEM; LM001-05, Welgene, Gyeongsan, South Korea) containing 10% fetal bovine serum (FBS; S001-01, Welgene) and 1% penicillin-streptomycin (PS; LS202-02, Welgene). The culture supernatants were collected at 48, 72, and 96 h after transfection. The lentiviral supernatant was filtered through a 0.45-µm PES syringe filter (16537, Sartorius, Göttingen, Germany) and stored at −80°C.

For lentivirus transduction, cells were cultured until 70–80% confluency in Roswell Park Memorial Institute (RPMI) 1640 media (LM011-01, Welgene) with 10% FBS and 1% PS. The lentiviral supernatant was thawed in a 37°C water bath and gently applied to cells with hexadimethrine bromide (Polybrene; 8 µg/ml, H9268, Sigma-Aldrich). The medium was changed after 18 h post transduction and was maintained for 1 day. Antibiotic selection was performed depending on the drug-resistance genes, until all non-transduced cells had been eliminated; this culture was then maintained for 7 days. Antibiotic-selected cells were pooled, and KO efficacy was verified using western blot analysis. All procedures were performed to maintain the multiplicity of infection (MOI) under 0.3 to prevent multiple lentiviral transductions.

Drugs and chemicals

The drugs and chemicals used in this study are listed in **Supplemental Table 3**. The indicated concentrations were applied for each experiment.

Cells and organoids

The genetically engineered mouse GC cells, S1 and S1M, were established in earlier studies [9,10]. Human GC cell lines, including SNU-484 (00484), SNU-668 (00668), SNU-719 (00719), NCI-N87 (60113), and MKN-74 (80104), were obtained from the Korean Cell Line Bank (KCLB, Seoul, South Korea). These cell lines were maintained in RPMI-1640 medium supplemented with 10% FBS, 2 mM L-Glutamine (25030149, Gibco), and 1% PS at 37°C in a 5% CO_2 humidified incubator. The HEK-293 (21573, KCLB) cell line was obtained from the KCLB and maintained in DMEM supplemented with 10% FBS and 1% PS.

Mouse stomach organoids were primarily cultured directly from mouse stomach tissues, as described previously [11,12]. To generate *Trp53*-KO mouse stomach organoids (P organoid), the gastric epithelium of Cre-dependent Cas9-GFP-*Trp53*$^{fl/fl}$ mice was used to culture organoids. Once the organoids were established, a Cre recombinase expression cassette was introduced into the organoids using transfection. Organoids were then grown with nutlin-3 (N6287-1MG, Sigma-Aldrich) for 1 week to select *Trp53*-KO clones.

Mice

Immunocompetent syngeneic mice, non-obese diabetic-severe combined immune deficient mice (NOD-SCID, NOD.CB17-Prkdc^{scid}), and NOD-SCID IL2rg^{null} (NSG) mice were used. Immunocompetent syngeneic mice with S1 and S1M cell lines were established as previously reported [9]. Four- to six-week-old NOD-SCID and NSG mice were purchased from Hanabio (Hwaseong-si, South Korea) and JAbio (Suwon-si, South Korea), respectively.

To generate *Trp53*-KO mouse stomach organoids (P organoid), a Cre recombinase-encoding plasmid (PX459_PGK_Cre) was transfected into gastric organoids from B6J.129(Cg)-*Gt(ROSA)26Sor*$^{tm1.1(CAG-cas9*,-EGFP)Fezh}$/J mice (026179, Jackson Laboratory, Bar Harbor, ME, USA), which express Cre-dependent Cas9, and B6.129P2-*Trp53*^{tm1Brn}/J mice (008462, Jackson Laboratory), which have loxP sites flanking exon 2–10 of *Trp53*, on a C57BL/6 background. Nutlin-3 selection was performed for 1 week.

Mice were housed at 22–24°C with a standard light-dark cycle (12:12 h) with freely accessible feed and water. Age-, sex-, and weight-matched mice were randomly grouped.

Animal experiments

Peritoneal dissemination model

For the peritoneal transplantation, Four- to six-week-old adult NOD-SCID or NSG mice were first anesthetized with isoflurane. Next, 5 × 10^6 GC cells suspended in 100 µl of 50% growth factor-reduced basement membrane matrix (Matrigel; 356231, Corning, Corning, NY, USA) were injected into the peritoneal cavity of the mice. During the entire experimental period, mice were monitored every other day for any signs of malignancy. Necropsies were performed either 4–5 weeks post-peritoneal injection or when the mice showed clinical signs of malignancy.

Spleno-hepatic metastasis model

For the splenic transplantation, Four- to six-week-old adult immunocompetent syngeneic mice were first anesthetized with isoflurane. Next, 2 × 10^6 GC cells suspended in 30 µl of 50% Matrigel were injected into the exposed spleen. Necropsies were performed either 3–4 weeks post-splenic injection or when the mice showed clinical signs of malignancy.

Subcutaneous-pulmonary metastasis model

For the subcutaneous transplantation, 5-week-old NOD-SCID mice were first anesthetized with isoflurane. Next, 3 × 10^6 S1M cells with in vivo luciferase expression were injected into the subcutaneous dorsal flank region. The tumor volume was monitored once a week to track its growth over time. Necropsies were performed either 9-weeks post-subcutaneous injection or when the mice showed clinical signs of malignancy.

In vivo competition model

To evaluate the survival advantages of specific KO cells over wild-type cells, an in vivo competition assay was performed. To generate *NF2*-KO SNU-484 cells, sgRNA lentivirus was used with guide sequences of non-targeting plasmid backbone (non-target plasmid backbone F: 5′-GGGTCTTCGAGAAGACAC-3′), *NF2* #1 (human *NF2* 10-3 F: 5′-GTACACAATCAAGGACACAG-3′), and *NF2* #2 (human *NF2* 10-8 F: 5′-GCTGGCTTCTTACGCCGTCC-3′). Third-generation lentiviral packaging plasmids were used to generate the lentivirus. Each lentivirus was transduced independently with 8 µg/ml of polybrene and subsequently selected using puromycin (2 µg/ml) for at

least 2 weeks. Then, 3.3 × 10^6 cells of each of control, *NF2*-KO #1, and *NF2*-KO #2 cells were mixed, and 1 × 10^7 cells were injected into the peritoneum of NOD-SCID mice (n = 4). The same biological replicates of cell pellets were preserved for genomic DNA extraction. Mice were monitored every other day, and mice showing clinical symptoms of cancer were monitored daily. After five weeks post-injection, mice were euthanized, and postmortem necropsy was performed. Malignant ascites with disseminated cancer cells were confirmed using a fluid smear with Diff Quick stain. Genomic DNA was extracted from malignant ascites and formerly preserved cell pellets using the DNeasy Blood & Tissue Kit (69504, Qiagen, Hilden, Germany) and was subjected to quantification of the integrated lentiviral sequence using amplicon next-generation sequencing.

RASA1-KO SNU-484 cells were generated using guide sequences from the non-targeting plasmid backbone (non-targeting plasmid backbone F: 5′-GGGTCTTCGAGAAGACGT-3′), *RASA1* #1 (Human *RASA1* 5-3 F: 5′-GGGAGGCCGGTATTATAAC-3′), and *RASA1* #2 (Human RASA1 #2 F: 5′-GCTTATAATTTACTAATGAC-3′) as described above. 1 × 10^7 cells, consisting of control, *RASA1*-KO #1, and *RASA1*-KO #2 cells mixed at a ratio of 1:1:1, were injected into the peritoneum of NSG mice (n = 5). Genomic DNA was extracted from metastatic tumor foci tissues and preserved cell pellets, and the integrated lentiviral sequence was quantified using quantitative PCR (qPCR) to confirm the competition assay. To validate the results of the competition assay, individual control (n = 3) and *RASA1*-KO #1 (n = 3) SNU-484 cells were each injected into the peritoneum of NSG mice at a total of 4 × 10^6 cells per injection. Five weeks post-injection, mice were euthanized, and a necropsy was performed to count the gross metastatic foci. Tissues were processed for routine histopathological analysis.

In vivo imaging system

To visualize peritoneal dissemination models of GC, we used an in vivo luciferase assay with the Lago X in vivo imaging system (Spectral Instruments Imaging, Tucson, AZ, USA). The gene expression cassette encoding luciferase was introduced into S1M-Cas9+ cells using the PiggyBac transposon system. Then, 5 × 10^6 cells were injected into the peritoneum of NOD-SCID mice, and tumors were allowed to develop. To monitor tumor growth, dissemination, and drug efficacy, D-Luciferin (150 mg/kg, i.v., LUCK-100, Gold Biotechnology, St. Louis, MO, USA) was injected via the tail vein. After 30 min, luciferase activity was measured using the Lago X system, and mice were

monitored weekly. To monitor drug efficacy, A-1155463 (9 mg/kg, intraperitoneal [i.p.], S7800, Selleck Chemicals, Houston, TX, USA), venetoclax (ABT-199; 12 mg/kg, per os [p.o.], HY-15531, MedChemExpress [MCE], Monmouth Junction, NJ, USA), and verteporfin (CL 318952; 10 mg/kg, i.p., HY-B0146, MCE) were administered according to the indicated schedules. Each experimental group consisted of four or five mice.

In vitro experiments

Organoid culture

Mouse gastric epithelial cells from Cre-dependent Cas9-expressing *Trp53*$^{fl/fl}$ mice were isolated and cultured in the organoid medium, as previously described [9], *Trp53*-KO mouse gastric organoids were generated by transfecting a Cre recombinase--encoding plasmid (PX459_PGK_Cre) into gastric organoids established from C57BL/6 background mice bearing Rosa26-LSL-spCas9-EGFP (B6J.129(B6N)-Gt(ROSA)26Sortm1[Cas9-EGFP]) and *Trp53* $^{fl/fl}$ (B6.129P2-Trp53tm1Brn/J). After transfection of Cre recombinase, organoids were treated with nutlin-3 (N6287-1MG, Sigma-Aldrich) for 1 week. The transfection of Cre recombinase was confirmed by GFP expression and KO of *Trp53* was confirmed by nutilin-3-resistance. *Trp53*-KO organoids (P organoid) were then transduced with a pKLV (50946, Addgene) based *Rasa1* targeting sgRNA lentivirus for additional KO and were selected using puromycin (2 μg/ml). The size, number, morphology, and differentiation of the organoids were assessed using a microscope (ECLIPSE Ts2, Nikon, Tokyo, Japan; DS-Fi3, Nikon) and analyzed.

Establishment of KO cell lines

GC cell lines with constant Cas9 expression were established as previously described. sgRNA lentiviral vectors were generated according to a published protocol [13]. Briefly, the mouse (67988 and 1000000053, Addgene) and human (67989 and 1000000049, Addgene) sgRNA sequences were achieved from the gRNA library. Different guide sequences targeting mouse *Rasa1* (#1, CTGAGACGCTCGGGCCGGG; #2, GACGCGGCTCCGCCACCGA) and human *RASA1* (#1, GGGAGGCCGGTATTATAAC; #2, GCTTATAATTTACTAATGAC), mouse *Nf2* (#1, CTTGGTATGCGGAGCACCG; #2, GAGCACGGACGCCTCGGGA) and human *NF2* (#1, GAGATGGAGTTCAATTGCG; #2, CTTGGTACGCAGAGCACCG or CTGGCTTCTTACGCCGTCC),

mouse *Bcl-xL* (#1, GCGCGGGAGGTGATTCCCA; #2, ACCAGCGGTTGAAGCGCTC), mouse *Bcl-2* (#1, ACCCCACCGAACTCAAAGA; #2, GTGGCAAAGCGTCCCCTCG), and mouse *Yap1* (#1, ACGTTCAGTTGCGAAAGCA; #2, TACCCTTACCTGTCGCGAG) were selected. As non-target control, GTGTAGTTCGACCATTCGTG and the intact plasmid backbone with the guide sequence of GGGTCTTCGAGAAGACGT were used. The paired complementary gRNA oligos were annealed, and then ligated (T4 DNA Ligase; M001, Enzynomics, Daejeon, South Korea) into BbsI-HF (R3539, New England Biolabs [NEB], Ipswich, MA, USA)-digested pKLV puromycin-resistance vectors (50946; 67974; 67975; 67977; Addgene), or BsmBI-v2 (R0739S, NEB)-digested lenti-sgRNA neomycin-resistance vector (lenti-sgRNA neo; 104992, Addgene) or Cas9-sgRNA-puromycin-resistance vector (Lenti-multi-CRISPR; 85402, Addgene), depending on their purpose. The ligated plasmids were transfected into DH5a (RH617, Real Biotech Corporation, Taipei, Taiwan) using the heat-shock method and plasmids were extracted using the QIAprep Spin Miniprep Kit (27106, Qiagen). All plasmids used in lentiviral production were confirmed using Sanger sequencing (U6 primer F: 5′-CAGTGCAGGGGAAAGAATAGTAGAC-3′).

Establishment of double-KO cell lines

To generate double-KO cell lines, we used mouse gastric cancer cell lines with stable Cas9 expression. For one-step establishment of double-KO cells, the lentiviral plasmid (pKLV2.2-h7SKgRNA5(SapI)-hU6gRNA5(BbsI)-PGKpuroBFP-W; 72666, Addgene, a gift from Kosuke Yusa) containing a dual sgRNA expression cassette along with a puromycin-resistance gene was employed. The sgRNA sequences targeting *Nf2* (CTTGGTATGCGGAGCACCG) and *Rasa1* (CTGAGACGCTCGGGCCGGG) were subcloned using BbsI-HF (R3539S, NEB) and SapI (R0569S, NEB) enzymes, respectively, via a Golden Gate Assembly.

For two-step establishment of double KO cells, the lentiviral sgRNA plasmids with antibiotic-resistance genes (puromycin [67974, Addgene] and neomycin [104992, Addgene]) were used. Plasmids were digested in BbsI-HF (R3539S, NEB) or BsmBI-v2 (R0739S, NEB), depending on their sequences. The guide sequences of control (GTGTAGTTCGACCATTCGTG), *Nf2* (CTTGGTATGCGGAGCACCG), *Rasa1* (CTGAGACGCTCGGGCCGGG), and *Yap1* (ACGTTCAGTTGCGAAAGCA) were obtained from the mouse genome wide gRNA library (67988, Addgene) and integrated into digested plasmids, respectively. The plasmids were transformed into

NEB Stable Competent E. coli (C3040H, NEB) by heating at 42°C for 1 min. The transformed bacteria were incubated on Luria–Bertani agar (244520, Becton, Dickinson [BD], Franklin Lakes, NJ, USA) supplemented with carbenicillin (100 μg/ml, 10177012, Gibco) at 37°C for 18 h. Antibiotic-selected colonies were picked and incubated in Luria–Bertani broth (244620, BD) supplemented with ampicillin (100 μg/ml, 11593027, Gibco) at 37°C, 200 rpm, for 18 h. Plasmids were extracted using the QIAprep Spin Miniprep Kit (27106, Qiagen). The lentivirus was generated using third-generation lentiviral packaging plasmids (K497500, Invitrogen) and PEI (MW 25,000; 23966-1, Polysciences) with HEK-293T cells. At first, cells were transduced with the puromycin-resistance sgRNA lentivirus and selected for 7 days with puromycin (2 μg/ml). Next, puromycin-resistant cells were transduced with the neomycin-resistance sgRNA lentivirus and selected for 7 days with G418 (200 μg/ml, geneticin, ant-gn-1, InvivoGen). The knockout efficacy of each gene was confirmed via western blot analysis.

Establishment of dual-luciferase reporter cell lines

For the dual-luciferase assay, a lentiviral EF-1α promoter-induced Renilla luciferase (pLX313-Renilla luciferase; 118016, Addgene, a gift from William Hahn & David Root), lentiviral Wnt reporter consisting of a tandem repeat of seven TCF/LEF binding sites (pXL010-Wnt dual (GFP-Fire) reporter; 40588, Addgene, a gift from Sean Palecek) and TEAD-YAP/TAZ reporter consisting of multimerized TEAD binding sites (HOP-flash; 83467, Addgene, a gift from Barry Gumbiner) were used. Lenti-HOP-Neo, the lentiviral TEAD-YAP/TAZ reporter, was constructed by subcloning TEAD binding sites–minimal promoter–firefly luciferase gene cassette into neomycin-resistance lentiviral backbone (lenti-sgRNA neo; 104992, Addgene, a gift from Brett Stringer) using KpnI (CR014S, Enzynomics), EcoRI (CR002S, Enzynomics) and T4 ligase.

Establishment of in vivo luciferase imaging cell lines

To establish an in vivo luciferase imaging system, the luciferase gene cassette with the neomycin-resistance gene was introduced into S1M-Cas9+ cells. KO of S1M-Cas9^{+}-Luciferase^{+} cells was induced using the sgRNA lentivirus based on puromycin-resistance plasmids (pKLV1; 50946, Addgene or pKLV2; 72666, Addgene, a gift from Kosuke Yusa). After 1 week of antibiotic selection, pooled cells were used for subsequent experiments.

Cell counts

To investigate the growth difference between control and knockout cells under monolayer culture conditions, cell counting and relevant viability tests were conducted. An equal number of cells were plated depending on the experimental setup, and all cells were cultured in RPMI 1640 media supplemented with 10% FBS and 1% PS. After 48 hours post-seeding, the cells were collected, and either cell counting or relevant viability tests were performed, depending on the specific assay used. For manual cell counting, 1×10^5 cells were seeded in 6-well plates, and a trypan blue exclusion assay was performed. For the cell counting kit-8 assay (CCK-8, CK04, Dojindo, Kumamoto, Japan), 2×10^3 cells were seeded in 96-well plates, and the absorbance was measured using an Infinite 200 PRO (Tecan, Männedorf, Switzerland) microplate reader. For cells expressing renilla luciferase, 2×10^4 cells were seeded in 12-well plates, and luciferase activity was measured using an Infinite 200 PRO (Tecan) microplate reader. The growth difference was compared by normalizing the results to control cells. Each experiment was performed with at least three replications and repeated at least three times.

Anoikis assay

Anoikis was induced using a previously published protocol [14]. Briefly, S1M cells from control and *Nf2*-KO samples were dissociated into single cells using TrypLE (12605-028, Invitrogen), and 5×10^4 single cells were seeded in a low-attachment plate (SPL3D Cell Floater; 390706, SPL) and were incubated for 24 h with constant shaking. After incubation, cells were harvested and 1×10^6 cells were stained with Annexin V Fluor 488 and propidium iodide (PI) solution for 15 min at RT in the dark, using the Annexin V Fluor 488 Apoptosis Detection kit (KTA0002, Abbkine, Wuhan, China) according to the manufacturer's instructions. Annexin V/PI positive cells were analyzed using fluorescence-activated cell sorting (FACS) using NovoCyte (Agilent). The proportion of Annexin V-positive cells was normalized to the total number of cells to obtain the relative percentage of apoptotic cells.

Soft agar colony formation assay

A soft agar colony formation assay was used to assess the contact-independent growth of cells. In this assay, 1 × 0.5% and 0.3% RPMI-agar media were prepared by mixing 2 × RPMI 1640 media (LM204-50, Welgene) with 20% FBS (S001-01, Welgene), 2% PS (LS202-02, Welgene), and equal

amounts of 1% and 0.6% noble agar solution (A5431, Sigma-Aldrich). For the supporting bottom layer, 1.5 ml of 0.5% RPMI-agar media was applied to 6-well cell culture plates (30006, SPL) and allowed to solidify for 20 min at RT. For the top cellular layer, 2–5 × 10^4 cells were suspended in 1.5 ml of 0.3% RPMI-agar media and applied to the bottom layer. The noble agar was solidified for 20 min at RT, and the plates were incubated under standard culture conditions for 7–21 days, depending on the cell line. When the colonies were visible to the naked eye, 1% Nitro Blue Tetrazolium chloride (J60230, Alfa Aesar, Tewksbury, MA, USA) solution was added and incubated for 18 h. The samples were then fixed using 10% neutral buffered formalin, and the results were analyzed.

ATP viability assay

The viability of cells and tumor spheres was measured using the CellTiter-Glo 3D Cell Viability Assay (G9683, Promega) according to the manufacturer's instructions. Briefly, 200 µl of 1:1 mixture of media and three-dimensional (3D) CellTiter-glo luminescent cell viability assay solution was added to each well. Luminescence was measured on a GloMax® 20/20 Luminometer (E5311; Promega), and the obtained luminescence values were subsequently normalized to the control samples.

Wnt and YAP/TAZ reporter assays

To quantify the activity of the Wnt/β-catenin pathway, we transduced Cas9-expressing mouse GC cells with two lentiviral vectors. The first lentiviral vector contained a *Renilla* luciferase reporter plasmid (pLX313-Renilla luciferase reporter; 118016, Addgene, a gift from William Hahn & David Root). The second lentiviral vector contained a Wnt reporter plasmid with a tandem repeat of seven TCF/LEF binding sites (pXL010-Wnt dual (GFP-Fire) reporter;40588, Addgene, a gift from Sean Palecek). These lentiviral vectors were sequentially transduced into the cells. Cells were treated with puromycin (2 µg/ml) for the Wnt reporter and hygromycin (100 µg/ml, Hygromycin B Gold; ant-hg-1, InvivoGen) for the *Renilla* luciferase reporter, 48 h post-transduction and were selected for 7 days each. KO was induced using lenti-sgRNA neo (104992, Addgene) or lentiCRISPRv2 neo (98292, Addgene)-based lentivirus, and antibiotic selection was maintained for 2 weeks using G418 (200 µg/ml). Wnt reporter activity was measured using a dual-luciferase reporter assay kit (E1980, Promega) according to the manufacturer's instructions, and luminescence was measured on a GloMax 20/20 (E5311, Promega) or Infinite 200 PRO (Tecan, Männedorf, Switzerland) luminometer.

The firefly luciferase activity was normalized to *Renilla* luciferase activity. Wnt-conditioned media or recombinant mouse Wnt3a (772301, BioLegend, San Diego, CA, USA) was applied according to the experimental conditions.

To quantify YAP/TAZ activity, we used a lentiviral TEAD reporter plasmid-based lentivirus. This consists of a tandem repeat of 8 × wild-type (8×wt) TEAD binding sites (HOP-flash; 83467, Addgene, a gift from Barry Gumbiner). The 8×wt TEAD binding domain–minimal promoter–firefly luciferase gene cassette was subcloned into the lenti-sgRNA neo plasmid (104992, Addgene), which comprised a lentiviral backbone with the neomycin-resistance gene. The TEAD reporter lentivirus was transduced into the Cas9-expressing mouse GC cells with stable *Renilla* luciferase expression. G418 was used for antibiotic selections. KO was induced using the pKLV2 plasmid (67974, Addgene) based lentivirus, with puromycin selections. The TEAD-YAP/TAZ activity was measured and normalized as described above.

To assess the in vivo Wnt reporter or YAP/TAZ reporter activities in peritoneal metastatic nodules and ascites of mice injected with S1M Wnt reporter or YAP/TAZ reporter cells, the metastatic foci were dissociated using the GentleMACS™ Tissue Dissociator (130-093-235, Miltenyi Biotec, Bergisch Gladbach, Germany) and the Tumor Dissociation Kit (130-096-730, Miltenyi Biotec). The dissociated cells were further treated with TrypLE and passed through a 40-μm cell strainer to ensure the removal of any remaining cell aggregates or debris. For ascites, peritoneal fluid was collected from the mice and centrifuged at 300 g for 3 minutes. The resulting pellets were resuspended in ACK lysing buffer (A10492-01, Gibco) for red blood cell (RBC) lysis. The cell pellets were then treated with TrypLE to obtain single cells. Subsequently, the resulting single cells from both metastatic foci and ascites were analyzed to measure the Wnt reporter or YAP/TAZ reporter activities, using a dual-luciferase reporter assay kit as described above.

siRNA knockdown assay

To perform the siRNA knockdown assay, S1M cells were transfected with siRNA targeting *Bcl-2* (5′-CUGCAAAUGCUGGACUGA-3′, Bioneer, Daejeon, South Korea) and *Bcl-xL* (5′-GGAGAGCGUUCAGUGAUCU-3′, Bioneer) using the NEPA21 Cuvette Electroporation system (Nepa Gene, Chiba, Japan) according to the manufacturer's protocol. Control scrambled siRNA was also transfected for comparison. For each well of a 6-well culture plate (30024, SPL), 1 μg of siRNA was

used for 1.25 × 10^5 cells. Electroporation parameters were set as follows: poring pulse (125 V, 5.0 ms in length with 50 ms intervals) and transfer pulse (30 V, 50 ms in length with 50 ms intervals), using 4 mm electroporation cuvettes (EC-004, Nepa Gene). After transfection, the cells were cultured for 48 h. Wnt3a was added as indicated in the experimental conditions. Knockdown efficacy was assessed through quantitative reverse transcription-quantitative PCR (RT-qPCR) analysis. All experiments were performed in triplicate.

Western blot analysis

Cells were lysed with T-PER™ Tissue Protein Extraction Reagent (78510, Thermo Fisher Scientific, Waltham, MA, USA) supplemented with Xpert Protease Inhibitor Cocktail Solution (100×) (P3100-001, GenDEPOT, Baker, TX, USA) and Xpert Phosphatase Inhibitor Cocktail Solution (100×) (P3200-001, GenDEPOT) as instructed by the manufacturer. Protein samples were mixed with Laemmli sample buffer (L1100-001, GenDEPOT) and boiled for 5 min at 100°C. The protein concentration was quantified using a Pierce™ BCA protein assay kit (23225, Thermo Fisher Scientific) and Epoch™ microplate spectrophotometer (BioTek, Winooski, VT, USA). Equal amounts of proteins were fractionated via electrophoresis on a 10% sodium dodecyl sulfate–polyacrylamide gel and transferred onto a polyvinylidene difluoride membrane (1620177, Bio-Rad, Hercules, CA, USA). Following blocking in 5% Difco skim milk (232100, BD) the membrane was incubated with antibodies that specifically recognized RASA1 (1:1000, ab40677, Abcam, Cambridge, UK), BCL-XL (1:500, sc-8392, Santa Cruz Biotechnology [SCBT], Dallas, TX, USA), BCL-2 (1:1000, A19693, ABclonal, Wuhan, China), Phospho-P44/42 MAPK (Erk1/2) (Thr202/Tyr204) (1:1000, 4370s, Cell Signaling Technology [CST], Danvers, MA, USA), Erk1/2 (1:1000, 4695S, CST), NF2 (1:2000, ab88957, Abcam), YAP/TAZ (1:1000, 8418S, CST), Phospho-YAP (Ser127) (1:1000, 13008T, CST), c-Myc (1:1000, 18583, CST), Cyclin D1 (1:1000, 2978, CST), Survivin (1:1000, 2808, CST), GAPDH (1:1000, sc-365062, SCBT), GAPDH (1:1000, bsm-33033M, Bioss, Woburn, MA, USA), and β-actin (1:1000, bsm-33036M, Bioss) at the recommended concentration. Horseradish peroxidase (HRP)-conjugated goat anti-mouse IgG antibody (1:2000, ADI-SAB-100J, Enzo Life Sciences, Farmingdale, NY, USA) or HRP-conjugated goat anti-rabbit IgG antibody (1:2000, ADI-SAB-300J, Enzo Life Sciences) were used as the secondary antibodies depending on primary antibodies at the recommended concentration.

Chemiluminescent detection was performed using an enhanced chemiluminescence detection kit (32109, Thermo Fisher Scientific) and ImageQuant™ LAS 4000 (GE HealthCare, Chicago, IL, USA).

Quantitative polymerase chain reaction

To assess the relative proportion of *RASA1*-KO and non-target SNU-484 cells in xenografts, we followed previously established methodology [15]. Briefly, qPCR analyses were performed to measure each gRNA fraction in the xenografts. After amplification of a common gRNA region shared by all integrated gDNA constructs, qPCR measurements were made to quantify specific gRNA sequences. The qPCR primers used in the present study are listed in **Supplemental Table 4**.

Quantitative reverse transcription polymerase chain reaction

To measure gene expression levels using Quantitative reverse transcription polymerase chain reaction (RT-qPCR), total cellular RNA was extracted with a HiYield Total RNA Mini Kit (YRB100, Real Biotech Corporation, Taipei, Taiwan) following the manufacturer’s instructions. cDNA was synthesized using the M-MLV cDNA Synthesis Kit (EZ006S, Enzynomics) according to the manufacturer's instructions. RT-qPCR was performed on a Quantstudio PCR System (Applied Biosystems, San Francisco, CA, USA) using ExcelTaq™ 2X Fast Q-PCR Master Mix (TQ1210, SMOBIO Technology Inc., Hsinchu, Taiwan). The qRT-PCR primers used in the present study are listed in **Supplemental Table 4**.

Histopathology

Hematoxylin and eosin staining

Mouse tissues were fixed in 10% neutral buffered formalin for 1 day and processed for routine formalin-fixed paraffin-embedded sample preparation using the standard methodology. Next, 3-μm-thick paraffin sections were prepared and stained with hematoxylin and eosin (H&E). Slides were mounted with resinous mounting media (4112, Richard-Allan Scientific, Canton, MI, USA). The area of metastatic tumor foci was measured using SlideViewer (3DHISTECH, Budapest, Hungary), and the depth of invasiveness was calculated by dividing the tumor area by its length along the major axis.

Immunohistochemistry staining

For Immunohistochemistry (IHC) staining, heat induced antigen retrieval with citrate buffer (pH 6.0, C9999, Sigma-Aldrich) and endogenous peroxidase quenching with 0.3% hydrogen peroxide (H325-500-AL, Fisher Chemical, Waltham, MA, USA) was performed. Antibodies against NF2 (1:2000, ab88957, Abcam), RASA1 (1:200, ab40677, Abcam), β-catenin (1:200, 610153, BD), Cyclin D1 (1:400, 2978, CST), Survivin (1:1600, 2808, CST), Ki-67 (1:400, ab16667, Abcam), and cleaved caspase 3 (c-Caspase 3; 1:300, 9664S, CST) were applied independently for 18 h at 4°C in a humidified chamber. For the negative control, primary antibodies were omitted. The host species-matched HRP-conjugated secondary antibody (ImmPRESS® HRP polymer detection kit, MP-7402 & MP-7452, Vector Laboratories, Newark, CA, USA) was applied to each primary antibody for 2 h at RT. The immunoreactivity of antigens was visualized using 3,3′-diaminobenzidine (Immpact DAB Substrate; SK-4105, Vector Laboratories) solution and then counterstained with Mayer's hematoxylin (H-3404-100, Vector Laboratories). Slides were mounted with resinous media. The intensity and positive cell rate were simultaneously graded. The level of immunoreactivity of RASA1, NF2, and β-catenin was evaluated by two different pathologists who were blinded to clinical information.

For tissue microarray (TMA) analysis, the human gastric cancer TMA slides (CQ2, 2 mm core, n = 59 and VA2, 1 mm core, n = 31; SuperBioChips, Seoul, South Korea) and related clinicopathological data were archived. The histologic grade was assigned as well differentiated, moderately differentiated, and poorly differentiated with signet ring carcinoma. The TNM stage was based on the AJCC Cancer Staging Manual (7th Edition) [16].

Immunofluorescence (IF) staining

For IF staining, paraffin sections were deparaffinized and hydrated. Antigen retrieval was performed using Target retrieval solution (S169984, DAKO, Santa Clara, CA, USA) by heating at 100 °C for 20 min. The slides were blocked with antibody diluent (S302283-2, DAKO) with TrueBlack® Lipofuscin Autofluorescence Quencher (23007, Biotium, CA, USA) for 2 h at RT. Antibodies of active β-catenin (1:50, 05-665, Merck, Darmstadt, Germany), YAP (1:60, sc-376830, SCBT), BCL-2 (1: 100, A19693, ABclonal), BCL-XL (1: 400, 2764, CST), Cas9 (1:250, 19526, SCBT), AQP5 (1:200, HPA065008, Atlas Antibodies, Switzerland), and PCNA (1:400, SC-56, SCBT) was incubated for overnight at 4°C. Secondary antibodies (1:200) of Alexa Fluor 488 donkey anti-rabbit IgG (ab150073, Abcam) and Alexa Fluor 568 donkey anti-mouse IgG antibody (A10037, Thermo Fisher Scientific) was applied for 2 h at RT. DAPI (4',6-diamidino-2-phenylindole) was used for nuclear counterstaining. Slides were

mounted with VECTASHIELD mounting media (H-1200, Vector Laboratories). The IF-stained slides were imaged using a confocal laser scanning microscope (LSM 800, ZEISS, Oberkochen, Germany).

Histopathological analysis

For the quantitative IHC/IF analysis of the peritoneal disseminated mouse gastric cancer model, whole scanned slides were analyzed using QuPath, an open-source software designed for digital pathology image analysis [17].

In the analysis of IHC samples from primary tumors, a semiquantitative analysis was conducted to obtain a percentile expression of Ki-67 or c-Caspase 3-positive cells within the primary tumors. The total number of Ki-67-positive or c-Caspase 3-positive cancer cells was counted. Subsequently, these numbers were divided by the total area of the analyzed region. The analysis was performed in at least three different areas of the sample.

In the analysis of IHC samples from peritoneal metastatic foci, the region of interest was focused on the peritoneal metastatic foci. Within this region, the total number of Cyclin D1-positive or Survivin-positive cancer cells was counted. Semiquantitative analysis using Histochemical Scoring System (H-score) was used with QuPath. Immunostaining intensity was scored on a scale of 1 to 3, and the score was multiplied by the number of cells in each category. To obtain a relative expression level of Cyclin D1 and Survivin in the metastatic foci, these numbers were then divided by the total number of cancer cells present in the metastatic foci.

In the analysis of IF of samples, spCas9 was used as a cancer cell indicator. To compare the β-catenin and YAP1 activities in the sample area, the total number of β-catenin-positive or YAP1-positive cancer cells was counted. Subsequently, these numbers were divided by the total number of cancer cells in the area to obtain a relative expression level of β-catenin and YAP1. The analysis was performed in at least three different areas of the sample.

References

1. Joung J, et al. Author Correction: Genome-scale CRISPR-Cas9 knockout and transcriptional activation screening. Nat Protoc. 2019;14:2259.

2. Bolger AM, et al.Trimmomatic: a flexible trimmer for Illumina sequence data. Bioinformatics. 2014;30:2114–20.

3. Li W, et al. MAGeCK enables robust identification of essential genes from genome-scale CRISPR/Cas9 knockout screens. Genome Biol. 2014;15:554.

4. Kim D, et al. HISAT: a fast spliced aligner with low memory requirements. Nat Methods. 2015;12:357–60.

5. Pertea M, et al. StringTie enables improved reconstruction of a transcriptome from RNA-seq reads. Nat Biotechnol. 2015;33:290–5.

6. Love MI, et al. Moderated estimation of fold change and dispersion for RNA-seq data with DESeq2. Genome Biol. 2014;15:550.

7. Raudvere U, et al. g:Profiler: a web server for functional enrichment analysis and conversions of gene lists (2019 update). Nucleic Acids Res. 2019;47:W191–8.

8. An H-W, et al. The loss of epithelial Smad4 drives immune evasion via CXCL1 while displaying vulnerability to combinatorial immunotherapy in gastric cancer. Cell Rep. 2022;41:111878.

9. Park JW, et al. Establishment and characterization of metastatic gastric cancer cell lines from murine gastric adenocarcinoma lacking Smad4, p53, and E-cadherin. Mol Carcinog. 2015;54:1521–7.

10. Park JW, et al. Stem Cells Antigen-1 Enriches for a Cancer Stem Cell-Like Subpopulation in Mouse Gastric Cancer. Stem Cells. 2016;34:1177–87.

11. Tan SH, et al. AQP5 enriches for stem cells and cancer origins in the distal stomach. Nature. 2020;578:437–43.

12. An H-W, et al. In vivo CRISPR-Cas9 knockout screening using quantitative PCR identifies thymosin beta-4 X-linked that promotes diffuse-type gastric cancer metastasis. Mol Carcinog

[Internet]. 2021; Available from: http://dx.doi.org/10.1002/mc.23326

13. Tzelepis K, et al. A CRISPR Dropout Screen Identifies Genetic Vulnerabilities and Therapeutic Targets in Acute Myeloid Leukemia. Cell Rep. 2016;17:1193–205.

14. Park JW, genesis induced by E-cadherin, p53, and Smad4 loss in mice. Mol Carcinog. 2018;57:947–54.

15. Kwon J-W, et al. A synergistic partnership between IL-33/ST2 and Wnt pathway through Bcl-xL drives gastric cancer stemness and metastasis. Oncogene [Internet]. 2022; Available from: http://dx.doi.org/10.1038/s41388-022-02575-5

16. Edge SB, Compton CC. The American Joint Committee on Cancer: the 7th edition of the AJCC cancer staging manual and the future of TNM. Ann Surg Oncol. 2010;17:1471–4.

17. Bankhead P, et al. QuPath: Open source software for digital pathology image analysis. Sci Rep. 2017;7:16878.
