# Supplementary material for: Combined inhibition of Bcl-2 family members and YAP induces synthetic lethality in metastatic gastric cancer with RASA1 and NF2 deficiency
Source: Mol Cancer. 2023 Sep 20;22:156. doi: 10.1186/s12943-023-01857-0 (PMC10510129; doi:10.1186/s12943-023-01857-0)
Supplement: Supplementary file 2 — Additional file 2: Supplemental Table 1. Correlation between RASA1 immunoreactivity and clinicopathological parame ters in human GC tissues. P-value, Chi squared test. [file 12943_2023_1857_MOESM2_ESM.pdf]

Supplemental Table 1. Correlation between RASA1 immunoreactivity and clinicopathological parameters in human GC tissues. *P*-value, Chi squared test.

Parameter	RASA1 IHC score					Total	*P*-value
	1	2	3	4	5		
**Histological grade**							
WD*	3 (3.5%)	2 (2.3%)	2 (2.3%)	1 (1.2%)	5 (5.8%)	13 (15.1%)	*P* = .030
MD*	3 (3.5%)	3 (3.5%)	4 (4.7%)	1 (1.2%)	5 (5.8%)	16 (18.6%)	
PD*	17 (19.8%)	16 (18.6%)	12 (14%)	10 (11.6%)	2 (2.3%)	57 (66.3%)	
Total	23 (26.7%)	21 (24.4%)	18 (20.9%)	12 (14%)	12 (14%)	86 (100%)	
**TNM T stage**							
T1	3 (3.8%)	3 (3.8%)	7 (9%)	1 (1.3%)	2 (2.6%)	16 (20.5%)	*P* = .178
T2	1 (1.3%)	1 (1.3%)	2 (2.6%)	2 (2.6%)	3 (3.8%)	9 (11.5%)	
T3	11 (14.1%)	9 (11.5%)	8 (10.3%)	6 (7.7%)	7 (9%)	41 (52.6%)	
T4	5 (6.4%)	5 (6.4%)	0 (0%)	2 (2.6%)	0 (0%)	12 (15.4%)	
Total	20 (25.6%)	18 (23.1%)	17 (21.8%)	11 (14.1%)	12 (15.4%)	78 (100%)	
**TNM N stage**							
N0	7 (9%)	3 (3.8%)	8 (10.3%)	1 (1.3%)	7 (9%)	26 (33.3%)	*P* = .001
N1	5 (6.4%)	2 (2.6%)	6 (7.7%)	2 (2.6%)	1 (1.3%)	16 (20.5%)	
N2	1 (1.3%)	1 (1.3%)	0 (0%)	3 (3.8%)	4 (5.1%)	9 (11.5%)	
N3	7 (9%)	12 (15.4%)	3 (3.8%)	5 (6.4%)	0 (0%)	27 (34.6%)	
Total	20 (25.6%)	18 (23.1%)	17 (21.8%)	11 (14.1%)	12 (15.4%)	78 (100%)	
**TNM M stage**							
M0	19 (22.1%)	18 (20.9%)	16 (18.6%)	9 (10.5%)	12 (14%)	74 (86%)	*P* = .492
M1	4 (4.7%)	3 (3.5%)	2 (2.3%)	3 (3.5%)	0 (0%)	12 (14%)	
Total	23 (26.7%)	21 (24.4%)	18 (20.9%)	12 (14%)	12 (14%)	86 (100%)	
**Stage**							
Stage 1	4 (4.7%)	3 (3.5%)	8 (9.3%)	0 (0%)	4 (4.7%)	19 (22.1%)	*P* = .037
Stage 2	5 (5.8%)	1 (1.2%)	4 (4.7%)	2 (2.3%)	4 (4.7%)	16 (18.6%)	
Stage 3	8 (9.3%)	10 (11.6%)	4 (4.7%)	5 (5.8%)	4 (4.7%)	31 (36%)	
Stage 4	6 (7%)	7 (8.1%)	2 (2.3%)	5 (5.8%)	0 (0%)	20 (23.3%)	
Total	23 (26.7%)	21 (24.4%)	18 (20.9%)	12 (14%)	12 (14%)	86 (100%)	

*WD, well differentiated cancer; MD, moderately differentiated cancer; PD, poorly differentiated cancer
