# Supplementary material for: Combined inhibition of Bcl-2 family members and YAP induces synthetic lethality in metastatic gastric cancer with RASA1 and NF2 deficiency
Source: Mol Cancer. 2023 Sep 20;22:156. doi: 10.1186/s12943-023-01857-0 (PMC10510129; doi:10.1186/s12943-023-01857-0)
Supplement: Supplementary file 3 — Additional file 3: Supplemental Table 2. Correlation between NF2 immunoreactivity and clinicopathological parameters in human GC tissues. P-value, Chi squared test. [file 12943_2023_1857_MOESM3_ESM.pdf]

**Supplemental Table 2.** Correlation between NF2 immunoreactivity and clinicopathological parameters in human GC tissues. P-value, Chi squared test.

| **Parameter** | **NF2 IHC Score** | | | | | **Total** | ***P*-value** |
|---|---|---|---|---|---|---|---|
| | 1 | 2 | 3 | 4 | 5 | | |
| **Histological grade** | | | | | | | |
| WD* | 1 (1.2%) | 2 (2.4%) | 1 (1.2%) | 5 (5.9%) | 4 (4.7%) | 13 (15.4%) | *P* = .006 |
| MD* | 0 (0%) | 2 (2.4%) | 2 (2.4%) | 7 (8.2%) | 5 (5.9%) | 16 (18.9%) | |
| PD* | 6 (7.1%) | 15 (17.6%) | 19 (22.4%) | 14 (16.5%) | 2 (2.4%) | 56 (66%) | |
| Total | 7 (8.3%) | 19 (22.4%) | 22 (26%) | 26 (30.6%) | 11 (13%) | 85 (100%) | |
| **TNM T stage** | | | | | | | |
| T1 | 0 (0%) | 5 (6.5%) | 3 (3.9%) | 5 (6.5%) | 3 (3.9%) | 16 (20.8%) | *P* = .265 |
| T2 | 1 (1.3%) | 0 (0%) | 1 (1.3%) | 4 (5.2%) | 3 (3.9%) | 9 (11.7%) | |
| T3 | 1 (1.3%) | 11 (14.3%) | 11 (14.3%) | 12 (15.6%) | 5 (6.5%) | 40 (51.9%) | |
| T4 | 0 (0%) | 2 (2.6%) | 6 (7.8%) | 4 (5.2%) | 0 (0%) | 12 (15.6%) | |
| Total | 2 (2.6%) | 18 (23.4%) | 21 (27.3%) | 25 (32.5%) | 11 (14.3%) | 77 (100%) | |
| **TNM N stage** | | | | | | | |
| N0 | 1 (1.3%) | 6 (7.8%) | 2 (2.6%) | 10 (13%) | 7 (9.1%) | 26 (33.8%) | *P* = .016 |
| N1 | 1 (1.3%) | 4 (5.2%) | 3 (3.9%) | 5 (6.5%) | 2 (2.6%) | 15 (19.5%) | |
| N2 | 0 (0%) | 3 (3.9%) | 3 (3.9%) | 1 (1.3%) | 2 (2.6%) | 9 (11.7%) | |
| N3 | 0 (0%) | 5 (6.5%) | 13 (16.9%) | 9 (11.7%) | 0 (0%) | 27 (35.1%) | |
| Total | 2 (2.6%) | 18 (23.4%) | 21 (27.3%) | 25 (32.5%) | 11 (14.3%) | 77 (100%) | |
| **TNM M stage** | | | | | | | |
| M0 | 2 (2.4%) | 18 (21.2%) | 18 (21.2%) | 24 (28.2%) | 11 (12.9%) | 73 (85.9%) | *P* = .001 |
| M1 | 5 (5.9%) | 1 (1.2%) | 4 (4.7%) | 2 (2.4%) | 0 (0%) | 12 (14.1%) | |
| Total | 7 (8.2%) | 19 (22.4%) | 22 (25.9%) | 26 (30.6%) | 11 (12.9%) | 85 (100%) | |
| **Stage** | | | | | | | |
| Stage 1 | 1 (1.2%) | 5 (5.9%) | 2 (2.4%) | 6 (7.1%) | 5 (5.9%) | 19 (22.4%) | *P* = .087 |
| Stage 2 | 0 (0%) | 4 (4.7%) | 4 (4.7%) | 5 (5.9%) | 3 (3.5%) | 16 (18.8%) | |
| Stage 3 | 1 (1.2%) | 8 (9.4%) | 8 (9.4%) | 10 (11.8%) | 3 (3.5%) | 30 (35.3%) | |
| Stage 4 | 5 (5.9%) | 2 (2.4%) | 8 (9.4%) | 5 (5.9%) | 0 (0%) | 20 (23.5%) | |
| Total | 7 (8.2%) | 19 (22.4%) | 22 (25.9%) | 26 (30.6%) | 11 (12.9%) | 85 (100%) | |

*WD, well differentiated cancer; MD, moderately differentiated cancer; PD, poorly differentiated cancer
