# Supplementary material for: Combined inhibition of Bcl-2 family members and YAP induces synthetic lethality in metastatic gastric cancer with RASA1 and NF2 deficiency
Source: Mol Cancer. 2023 Sep 20;22:156. doi: 10.1186/s12943-023-01857-0 (PMC10510129; doi:10.1186/s12943-023-01857-0)
Supplement: Supplementary file 4 — Additional file 4: Supplemental Table 3. Information of drugs and chemicals used in this study. [file 12943_2023_1857_MOESM4_ESM.pdf]

Supplemental Table 3. Information of drugs and chemicals used in this study.

Product	Catalog no.	Manufacturer
Venetoclax (ABT-199)	HY-15531	MedChemExpress
BH3I-1	sc-221352	Santa Cruz Biotechnology
A-1155463	HY-19725	MedChemExpress
Trametinib (GSK1120212)	S2673	Selleck Chemicals
Verteporfin (CL 318952)	HY-B0146	MedChemExpress
Recombinant mouse Wnt3a	772301	BioLegend
Doxycycline	D9891	Sigma-Aldrich
D-Luciferin	LUCK-100	Gold Biotechnology
Growth factor reduced basement membrane matrix	356231	Corning
Nutlin-3	N6287-1MG	Sigma-Aldrich
Hexadimethrine bromide (Polybrene, PB)	H9268	Sigma-Aldrich
Blasticidin	ant-bl-1	InvivoGen
G418 (Geneticin)	ant-gn-1	InvivoGen
Puromycin	ant-pr-1	InvivoGen
Hygromycin	ant-hg-1	InvivoGen
Transport 5 transfection reagent	26008-5	Polysciences
Polyethylenimine (linear, MW 25000)	23966-1	Polysciences
Lipofectamine 2000	11668027	Invitrogen
Lipofectamine LTX	15338030	Invitrogen
