# Supplementary material for: Combined inhibition of Bcl-2 family members and YAP induces synthetic lethality in metastatic gastric cancer with RASA1 and NF2 deficiency
Source: Mol Cancer. 2023 Sep 20;22:156. doi: 10.1186/s12943-023-01857-0 (PMC10510129; doi:10.1186/s12943-023-01857-0)
Supplement: Supplementary file 5 — Additional file 5: Supplemental Table 4. Primer sequences for qPCR and RT-qPCR. [file 12943_2023_1857_MOESM5_ESM.pdf]

Supplemental Table 4. Primer sequences for qPCR and RT-qPCR

Gene	Forward (5′ to 3′)	Reverse (5′ to 3′)
**Primer sets used in qPCR**		
Common gRNA	AGGGCCTATTTCCCATGATTCCTTC	TCAAGTTGATAACGGACTAGC
Non-target gRNA	TGTGGAAAGGACGAAACACC	GTCTTCTCGAAGACCCGGTG
RASA1 gRNA #1	TGTGGAAAGGACGAAACACC	AAACGTTATAATACCGGCCTCCC
RASA1 gRNA #2	TGTGGAAAGGACGAAACACC	AAACGTCATTAGTAAATTATAAGC
**Primer sets used in RT-qPCR**		
Gapdh	GGTCGGTGTGAACGGATTTG	GTGAGTGGAGTCATACTGGAAC
Actb	GAACATGGCATTGTTACCAACTG	GTGTTGAAGGTCTCAAACATGATC
Lgr5	AGAGCCTGATACCATCTGCAAAC	TGAAGGTCGTCCACACTGTTGC
Aqp5	TCCATGAACCCAGCCCGATCTT	GAAGTAGAGGATTGCAGCCAGG
Bcl-xL	CGGAGAGCGTTCAGTGATC	CTGCATCTCCTTGTCTACGC
Bcl-2	CCTGTGGATGACTGAGTACCTG	AGCCAGGAGAAATCAAACAGAGG
Cd44	CACTGTGACTCATGGATCCGA	GGCAAGAATCAGAGCCAGTG
c-Myc	ATCCTGTACCTCGTCCGATTCC	GGTTTGCCTCTTCTCCACAGAC
Ccnd1	GCAGAAGGAGATTGTGCCATCC	AGGAAGCGGTCCAGGTAGTTCA
Ctgf	TGCGAAGCTGACCTGGAGGAAA	CCGCAGAACTTAGCCCTGTATG
Cyr61	GTGAAGTGCGTCCTTGTGGACA	CTTGACACTGGAGCATCCTGCA
Igfbp4	CGGAGCAAGATGAAGATCGTGG	GATGAAGAGGTCTTCGTGGGTAC
Wnt5a	GGAACGAATCCACGCTAAGGGT	AGCACGTCTTGAGGCTACAGGA
Bmp4	GCCGAGCCAACACTGTGAGGA	GATGCTGCTGAGGTTGAAGAGG
