# Supplementary material for: Combined inhibition of Bcl-2 family members and YAP induces synthetic lethality in metastatic gastric cancer with RASA1 and NF2 deficiency
Source: Mol Cancer. 2023 Sep 20;22:156. doi: 10.1186/s12943-023-01857-0 (PMC10510129; doi:10.1186/s12943-023-01857-0)
Supplement: Supplementary file 6 — Additional file 6: Supplemental Figure 1. Establishment of the three-dimensional tumorsphere culture system. [file 12943_2023_1857_MOESM6_ESM.pdf]

Supplemental Figure 1

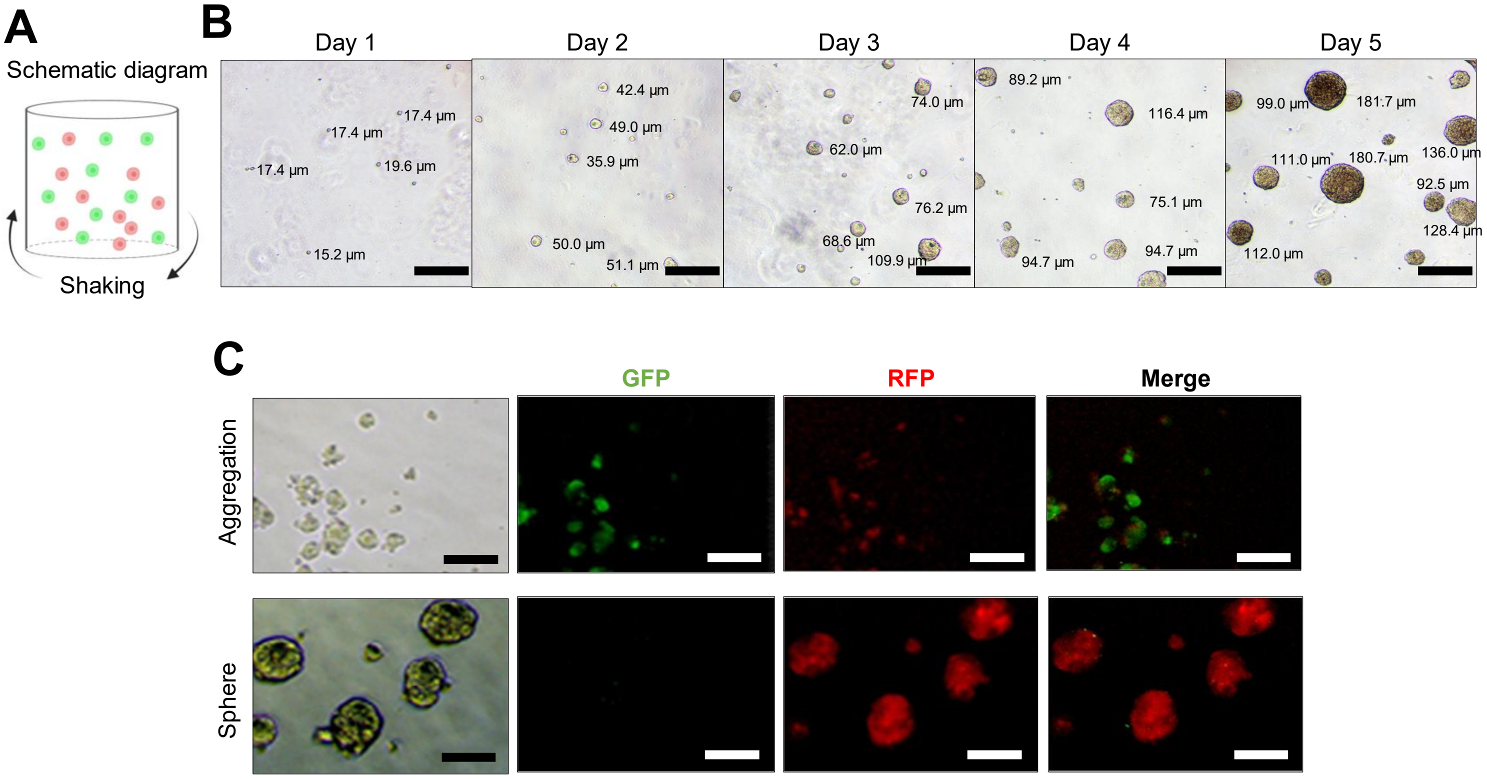

Supplemental Figure 1. Establishment of the three-dimensional tumorsphere culture system

(**A**) Schematic view of the 3D tumorsphere culture system used to confirm single-cell origin of tumorspheres without cellular aggregations. Equal numbers of GFP^{+} and RFP^{+} S1 cells were mixed and incubated for 5 days, and the growth patterns were monitored. To prevent cellular aggregations, cells were cultured under constant shaking.

(**B**) Chronological morphological characteristics of the tumorsphere growth were monitored at each time point for 5 days using a light microscope. At the initial seeding point, no cellular doublets were observed. As time progressed, the single cells formed individual tumorspheres and increased in size. Bar = 200 μm

(**C**) Representative images of the tumorsphere formation assay using GFP^{+} and RFP^{+} S1 cells. At day 5, most of the globoid tumorspheres larger than 50 μm diameter were exclusively GFP-positive or RFP-positive. A small fraction of cellular aggregates characterized by a diameter less than 50 μm exhibited a mixed cellular population positive for both GFP and RFP. Bar = 50 μm
