# Supplementary material for: Combined inhibition of Bcl-2 family members and YAP induces synthetic lethality in metastatic gastric cancer with RASA1 and NF2 deficiency
Source: Mol Cancer. 2023 Sep 20;22:156. doi: 10.1186/s12943-023-01857-0 (PMC10510129; doi:10.1186/s12943-023-01857-0)
Supplement: Supplementary file 7 — Additional file 7: Supplemental Figure 2. In vivo validation of target genes using the peritoneal dissemination model. [file 12943_2023_1857_MOESM7_ESM.pdf]

## Supplemental Figure 2

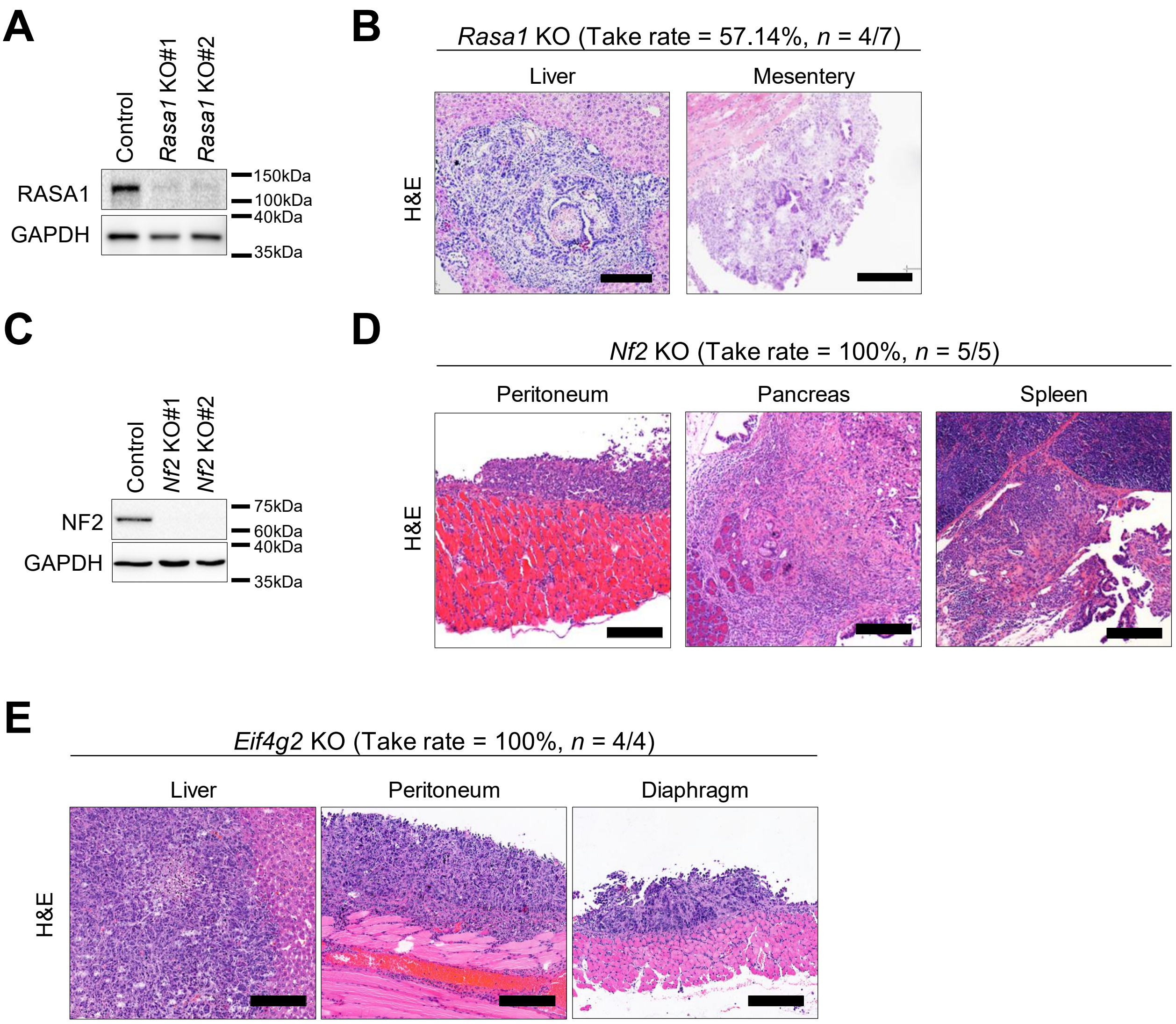


**Supplemental Figure 2. In vivo validation of target genes using the peritoneal dissemination model**

(**A**) Representative western blot analysis of RASA1 in control and *Rasa1*-KO S1M cells. The KO efficacy of *Rasa1* in S1M cells was confirmed using western blot analysis.

(**B**) Representative H&E images of metastatic foci in the liver and mesentery from syngeneic mice intraperitoneally injected with *Rasa1*-KO S1M cells (*n* = 7). Necropsy was performed either upon mice showing clinical signs of malignancy or at 105 days post-injection. Bar = 200 μm.

(**C**) Representative western blot analysis of NF2 in control and *Nf2*-KO S1M cells. The KO efficacy of *Nf2* in S1M cells was confirmed using western blot analysis.

(**D**) Representative H&E images of metastatic foci in the peritoneum, pancreas, and spleen from syngeneic mice intraperitoneally injected with *Nf2-KO* S1M cells (*n* = 5). Necropsy was performed upon mice showing clinical signs of malignancy. Bar = 200 μm.

(**E**) Representative H&E images of metastatic foci in the liver, peritoneum, and diaphragm from syngeneic mice intraperitoneally injected with *Eif4g2*-KO S1M cells (*n* = 5). Necropsy was performed upon mice showing clinical signs of malignancy. Bar = 200 μm.
