# Supplementary material for: Combined inhibition of Bcl-2 family members and YAP induces synthetic lethality in metastatic gastric cancer with RASA1 and NF2 deficiency
Source: Mol Cancer. 2023 Sep 20;22:156. doi: 10.1186/s12943-023-01857-0 (PMC10510129; doi:10.1186/s12943-023-01857-0)
Supplement: Supplementary file 8 — Additional file 8: Supplemental Figure 3. In vivo validation of target genes using the spleno-hepatic metastasis model. [file 12943_2023_1857_MOESM8_ESM.pdf]

Supplemental Figure 3

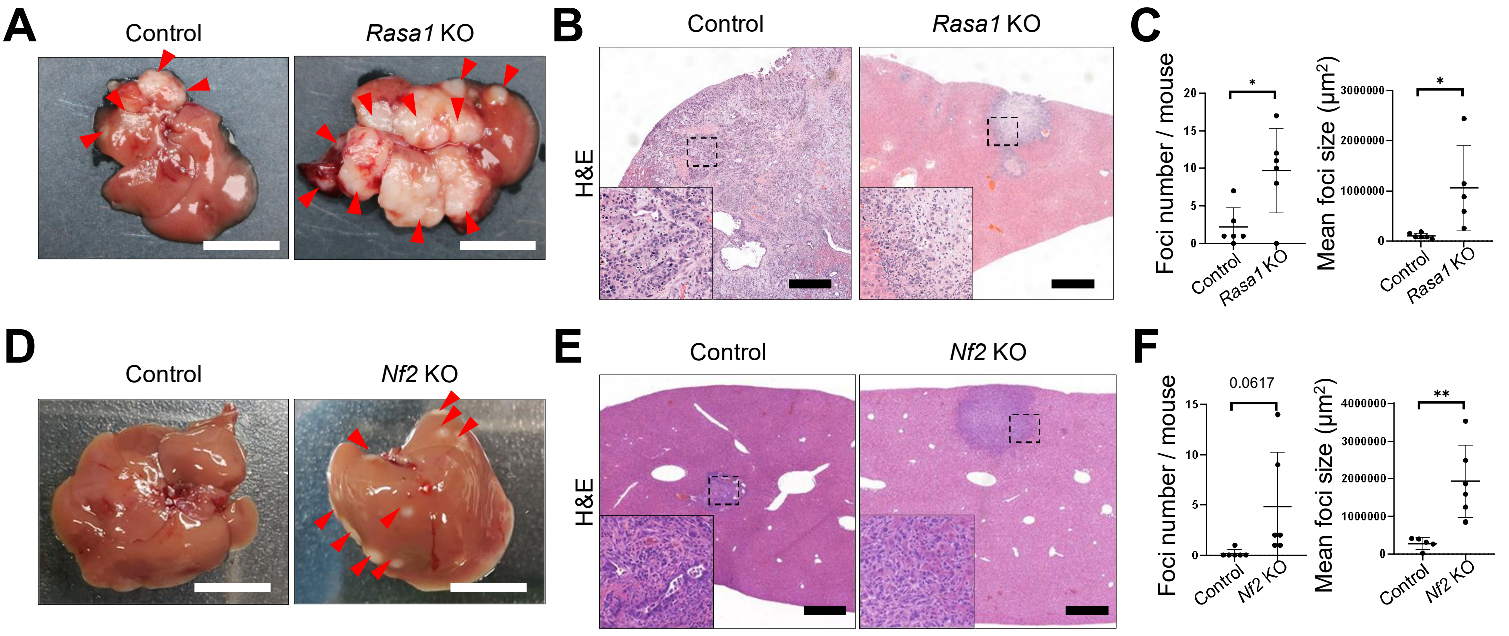

Supplemental Figure 3. In vivo validation of target genes using the spleno-hepatic metastasis model

(**A**) Representative gross images of hepatic metastases in syngeneic mice injected with control (n = 6) and *Rasa1*-KO (n = 6) S1M cells via spleen. Necropsy was performed 4 weeks post-injection. Bar = 1 cm.

(**B**) Representative H&E images of metastatic foci in the liver from syngeneic mice injected with control (n = 6) and *Rasa1-KO* (n = 6) S1M cells via spleen. Bar = 500 μm.

(**C**) Statistical analysis of the total number of macro-metastatic foci per mouse (**left**) and mean micro-metastatic foci area (**right**) in hepatic metastases of syngeneic mice injected with control (n = 6) or *Rasa1*-KO (n = 5) S1M cells via spleen.

(**D**) Representative gross images of hepatic metastases in syngeneic mice injected with control (n = 5) and *Nf2*-KO (n = 6) S1M cells via spleen. Necropsy was performed 3 weeks post-injection. Bar = 1 cm.

(**E**) Representative H&E images of metastatic foci in the liver from syngeneic mice injected with control (n = 5) and *Nf2*-KO (n = 6) S1M cells via spleen. Dashed line; metastatic foci, Bar = 500 μm.

(**F**) Statistical analysis of the total number of macro-metastatic foci per mouse (**left**) and mean micro-metastatic foci area (**right**, μm^2) in hepatic metastases of syngeneic mice injected with control (n = 5) or *Nf2*-KO (n = 6) S1M cells via spleen. P value, Student's t-test.
