# Supplementary material for: Combined inhibition of Bcl-2 family members and YAP induces synthetic lethality in metastatic gastric cancer with RASA1 and NF2 deficiency
Source: Mol Cancer. 2023 Sep 20;22:156. doi: 10.1186/s12943-023-01857-0 (PMC10510129; doi:10.1186/s12943-023-01857-0)
Supplement: Supplementary file 9 — Additional file 9: Supplemental Figure 4. Effect of NF2 and RASA1 deficiency on metastasis in human GC. [file 12943_2023_1857_MOESM9_ESM.pdf]

Supplemental Figure 4

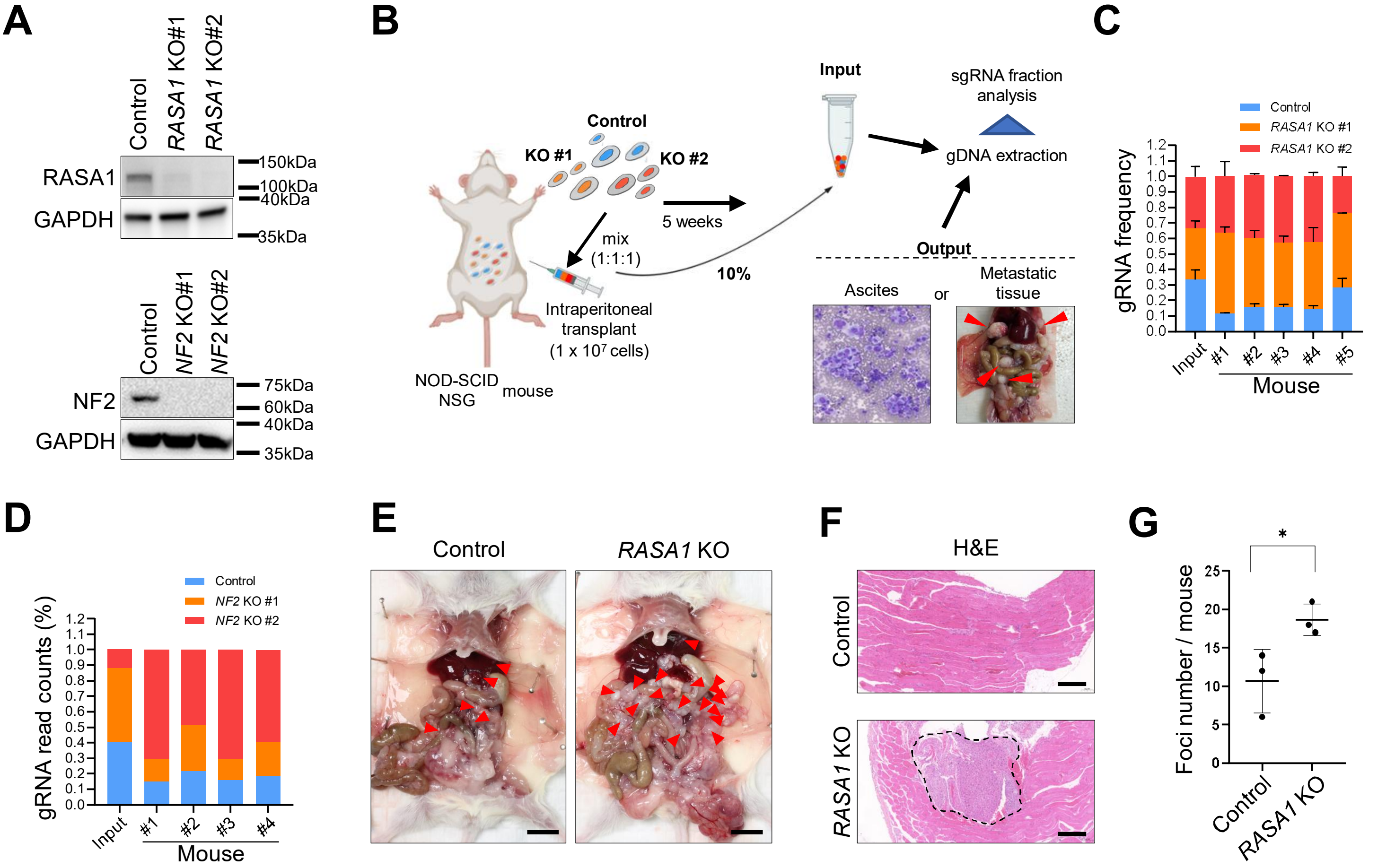

Supplemental Figure 4. Effect of NF2 and RASA1 deficiency on metastasis in human GC

(**A**) Representative western blot analysis of RASA1 and NF2 in control and *RASA1*-KO (**top**)/*NF2*-KO (**bottom**) SNU-484 cells. KO efficacy of *RASA1* (**top**) and *NF2* (**bottom**) was confirmed by western blot analysis.

(**B**) A schematic view of the in vivo competitive assay to validate the survival advantages of *RASA1*- and *NF2*-KO cells in peritoneal dissemination of gastric cancer using SNU-484 cells in NSG or NOD-SCID mice. Equal numbers of control, KO #1, and KO #2 SNU-484 cells were peritoneally injected, and 10% of the injected samples were preserved as Input samples for further analysis. At the time of necropsy, metastatic tissues or malignant ascites were collected, and genomic DNA was extracted. The relative gRNA sequence frequency targeting control, KO #1, and KO #2 was analyzed using qPCR or amplicon sequencing.

(**C**) qPCR analysis of gRNAs frequencies targeting control and *RASA1* using genomic DNA from metastatic foci of NSG mice (n = 5). Necropsy was performed five weeks post-injection.

(**D**) Relative NGS read counts of gRNAs targeting control and *NF2* using genomic DNA from malignant ascites of NOD-SCID mice with amplicon sequencing (n = 4). Necropsy was performed five weeks post-injection.

(**E**) Gross images of peritoneal metastasis in NSG mice peritoneally injected with control (n = 3) and *RASA1-KO* (n = 3) SNU-484 cells. Arrowheads indicate metastatic nodules. Bar = 1 cm

(**F**) Representative H&E images of peritoneal metastatic foci in the diaphragm of NSG mice peritoneally injected with control (n = 3) and *RASA1-KO* (n = 3) SNU-484 cells. Bar = 200 μm. Dashed lines indicate tumor area.

(**G**) Statistical analysis of macro-metastatic foci in peritoneal metastases from NSG mice injected with control (n = 3) or *RASA1*-KO (n = 3) SNU-484 cells. P value, Student's t-test.
