# Supplementary material for: Combined inhibition of Bcl-2 family members and YAP induces synthetic lethality in metastatic gastric cancer with RASA1 and NF2 deficiency
Source: Mol Cancer. 2023 Sep 20;22:156. doi: 10.1186/s12943-023-01857-0 (PMC10510129; doi:10.1186/s12943-023-01857-0)
Supplement: Supplementary file 10 — Additional file 10: Supplemental Figure 5. The effects of NF2 and RASA1 deficiency on monolayer growth. [file 12943_2023_1857_MOESM10_ESM.pdf]

Supplemental Figure 5

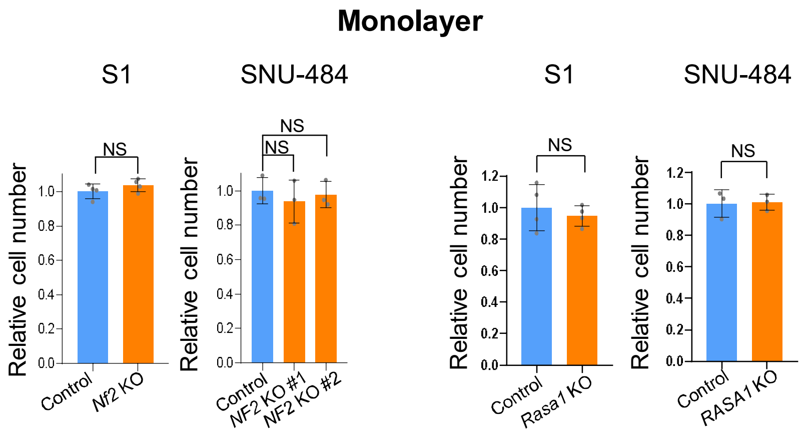

Supplemental Figure 5. The effects of NF2 and RASA1 deficiency on monolayer growth

Relative growth difference in monolayer culture of S1 mouse gastric cancer (GC) cells and SNU-484 human GC cells depending on *NF2*- and *RASA1*-KO status. An equal number of cells were seeded on the cell culture plates and incubated with RPMI-1640 media supplemented with 10% FBS and 1% PS for 48 h. Cell counts were normalized to control samples and representative relative cell counts are shown. For analysis of S1 cells with *Nf2*-KO, renilla luciferase was used to measure the relative cell counts. For analysis of SNU-484 cells with *NF2*-KO, CCK-8 assay was used to measure the relative cell counts. For analysis of S1 cells with *Rasa1*-KO and SNU-484 cells with *RASA1*-KO, manual trypan blue exclusion was used to measure the relative cell counts. Differences in relative cell counts were analyzed using the Student's t-test.
