# Supplementary material for: Combined inhibition of Bcl-2 family members and YAP induces synthetic lethality in metastatic gastric cancer with RASA1 and NF2 deficiency
Source: Mol Cancer. 2023 Sep 20;22:156. doi: 10.1186/s12943-023-01857-0 (PMC10510129; doi:10.1186/s12943-023-01857-0)
Supplement: Supplementary file 11 — Additional file 11: Supplemental Figure 6. Analysis of RNA sequencing data from Rasa1- and Nf2-KO S1 Tumorspheres. [file 12943_2023_1857_MOESM11_ESM.pdf]

Supplemental Figure 6

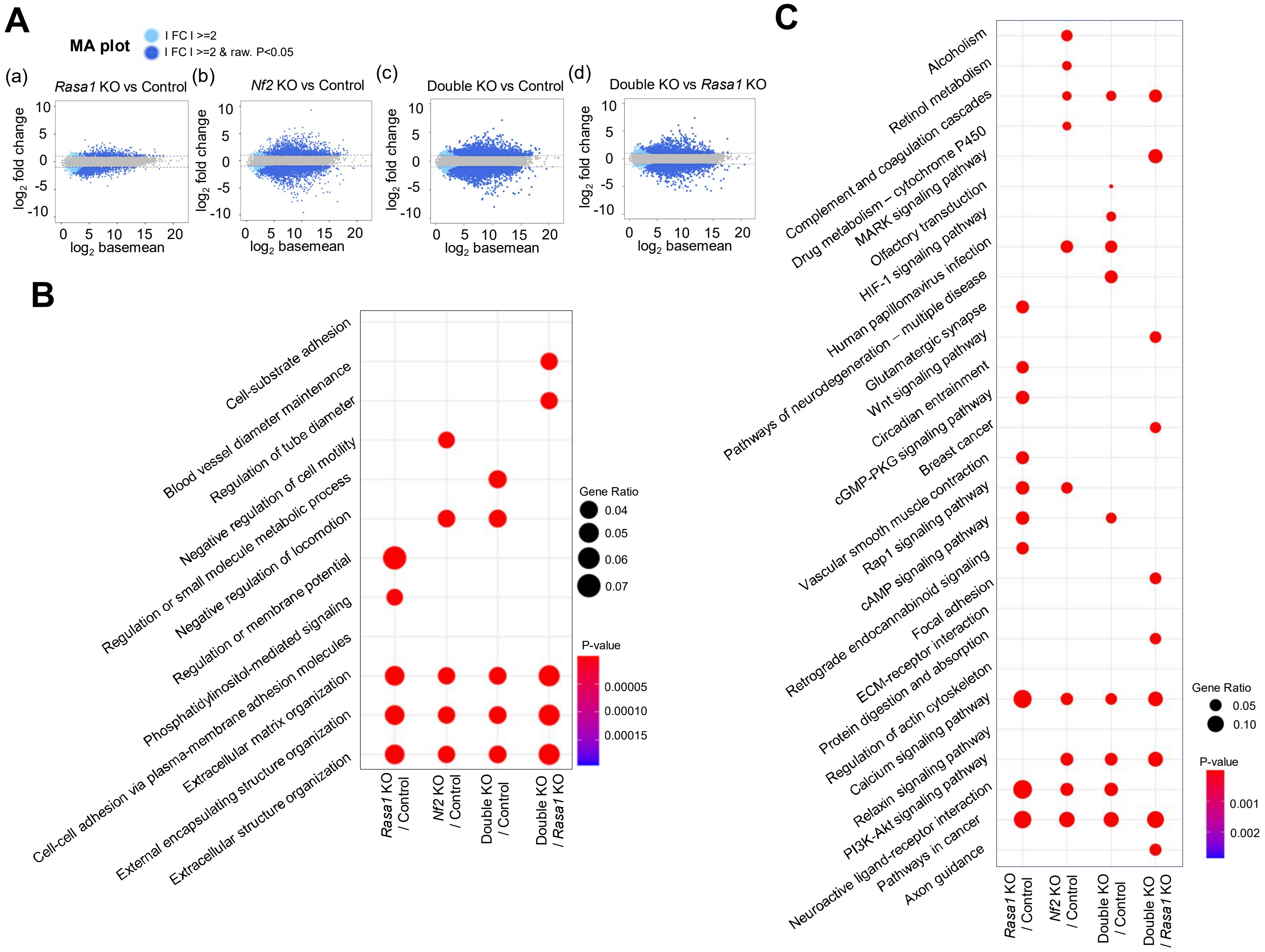

Supplemental Figure 6. Analysis of RNA sequencing data from *Rasa1*- and *Nf2*-KO S1 Tumorspheres.

(**A**) MA plots showing differential gene expression for the following: (**a**) *Rasa1*-KO versus control, (**b**) *Nf2*-KO versus control, (**c**) *Rasa1*/*Nf2*-double-KO versus control, and (**d**) *Rasa1*/*Nf2*-double-KO versus *Rasa1*-KO. Significantly upregulated or downregulated (with fold change >2 and *P* value < 0.05) genes are indicated in blue. Each dot represents a single gene.

(**B**) Gene Ontology (GO) enrichment analysis of *Rasa1*-KO versus control, *Nf2*-KO versus control, *Rasa1*/*Nf2*-double-KO versus control, and *Rasa1*/*Nf2*-double-KO versus *Rasa1*-KO S1 tumorspheres. Genes displaying >3-fold change in expression across comparisons demonstrated prominent shifts within biological processes. Dot color and size represent the *P* value and gene ratio (gene counts in specific term/total genes), respectively.

(**C**) Kyoto Encyclopedia of Genes and Genomes (KEGG) pathway enrichment analysis of *Rasa1*-KO versus control, *Nf2*-KO versus control, *Rasa1*/*Nf2*-double-KO versus control, and *Rasa1*/*Nf2*-double-KO versus *Rasa1*-KO S1 tumorspheres. Differentially expressed genes with >3-fold changes revealed the most pronounced changes in Pathways in cancer (KEGG 05200). Dot color and size represent the *P* value and gene ratio (gene counts in specific term/total genes), respectively.
