# Supplementary material for: Combined inhibition of Bcl-2 family members and YAP induces synthetic lethality in metastatic gastric cancer with RASA1 and NF2 deficiency
Source: Mol Cancer. 2023 Sep 20;22:156. doi: 10.1186/s12943-023-01857-0 (PMC10510129; doi:10.1186/s12943-023-01857-0)
Supplement: Supplementary file 12 — Additional file 12: Supplemental Figure 7. Western blot analysis for YAP signal activation resulting from NF2 deficiency. [file 12943_2023_1857_MOESM12_ESM.pdf]

Supplemental Figure 7

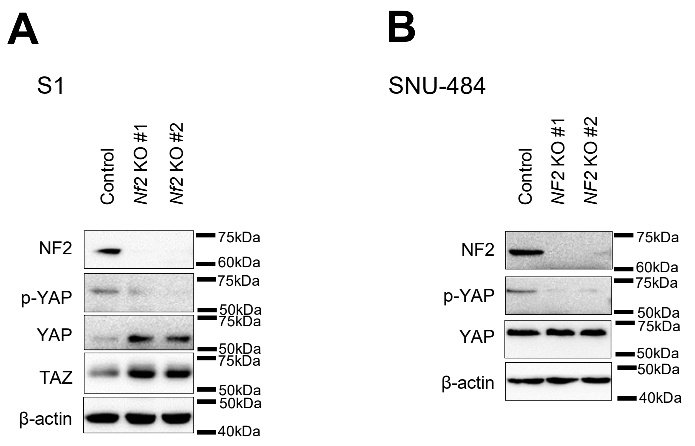

Supplemental Figure 7. Western blot analysis for YAP signal activation resulting from NF2 deficiency

(**A**) Representative western blot analysis of phosphorylated (p)-YAP, YAP, and TAZ in control and *Nf2*-KO S1 cells. S1 cells were seeded at a density of 1 × 10^4 in 6-well plates with RPMI-1640 media supplemented with 10% FBS and 1% PS. After 48 hours post-seeding, cells were harvested and subjected to western blot analysis. (**B**) Representative western blot analysis of p-YAP and YAP in control and *NF2*-KO SNU-484 cells. SNU-484 cells were seeded at a density of 2 × 10^4 in 6-well plates with RPMI-1640 media supplemented with 10% FBS and 1% PS. After 72 hours post-seeding, cells were harvested and subjected to western blot analysis.
