# Supplementary material for: Combined inhibition of Bcl-2 family members and YAP induces synthetic lethality in metastatic gastric cancer with RASA1 and NF2 deficiency
Source: Mol Cancer. 2023 Sep 20;22:156. doi: 10.1186/s12943-023-01857-0 (PMC10510129; doi:10.1186/s12943-023-01857-0)
Supplement: Supplementary file 13 — Additional file 13: Supplemental Figure 8. NF2 deficiency induces anoikis resistance via YAP activation. [file 12943_2023_1857_MOESM13_ESM.pdf]

Supplemental Figure 8

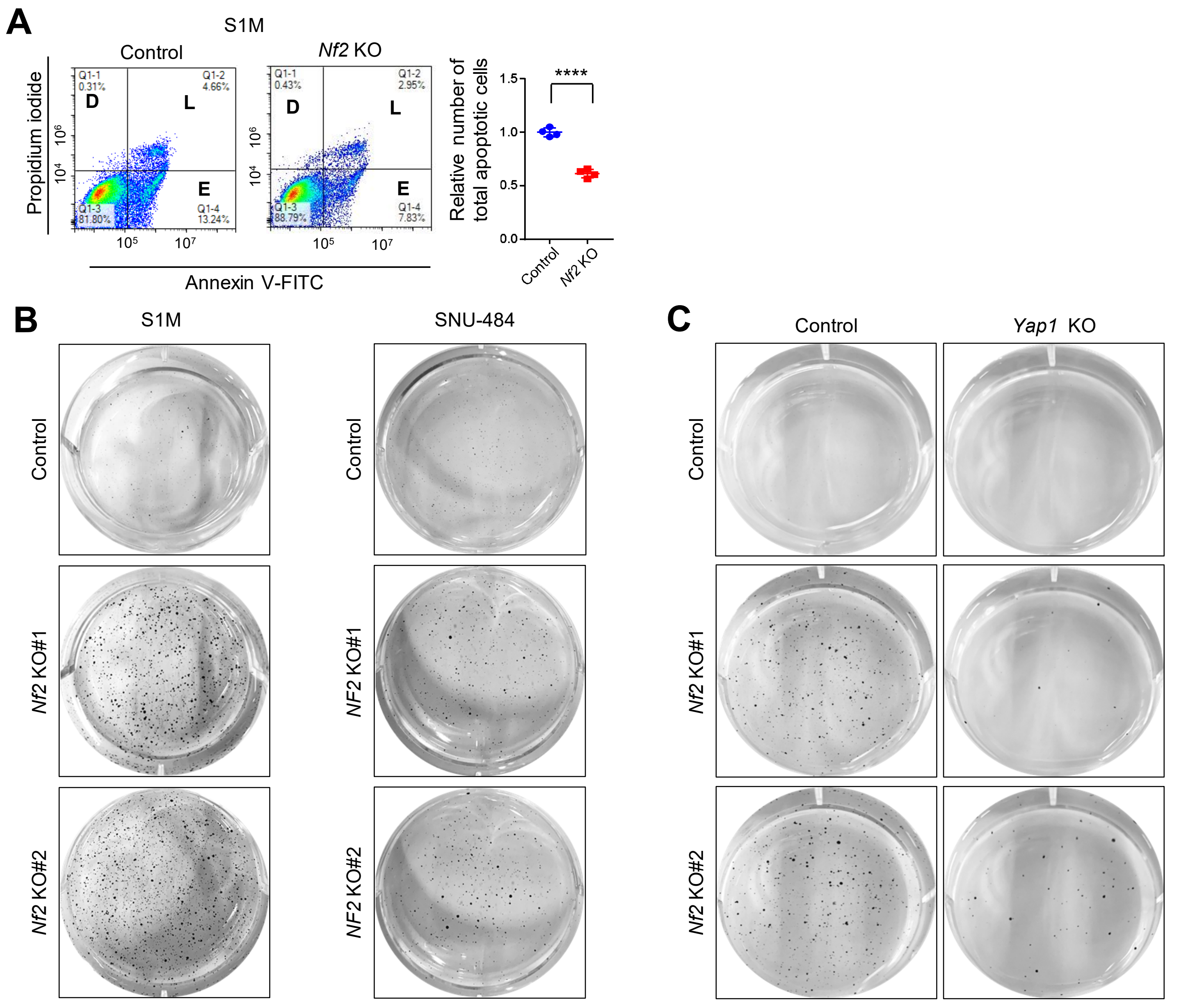

Supplemental Figure 8. NF2 deficiency induces anoikis resistance via YAP activation

(**A**) (**left**) Flow cytometry analysis of anoikis assay under low-attachment conditions in control and *Nf2-KO* S1M cells using Annexin V–propidium iodide stains. D, dead cells; E, early apoptotic cells; L, late apoptotic cells. 5×10^4 single cells were seeded in a low-attachment plate and were incubated for 24 h with constant shaking. (**right**) Statistical analysis of relative total apoptotic cells (Annexin V^+) in anoikis assay with control and *Nf2*-KO S1M cells. *P* value, Student's *t*-test.

(**B**) (**left**) Representative images of soft agar colony formation assay with control and *Nf2*-KO S1M cells. 2×10^4 cells were suspended in 1.5 ml of 0.3% RPMI-agar media in 6 well plates. Cells were incubated under standard culture conditions for 12 days. (**right**) Representative images of soft agar colony formation assay with control and *NF2*-KO SNU-484 cells. 5×10^4 cells were suspended in 1.5 ml of 0.3% RPMI-agar media in 6 well plates. Cells were incubated under standard culture conditions for 20 days. Nitro Blue Tetrazolium chloride was used to stain colonies.

(**C**) Representative images of soft agar colony formation assay with control and *Nf2*-KO S1M cells with concurrent *Yap1*-KO to evaluate the effect of YAP ablation on gastric cancer clonogenicity. 2×10^4 cells of control, *Nf2*-, *Yap1*-, *Nf2*/*Yap1*-double-KO S1M cells were suspended in 1.5 ml of 0.3% RPMI-agar media in 6 well plates. Cells were incubated under standard culture conditions for 12 days. Nitro Blue Tetrazolium chloride was used to stain colonies.
