# Supplementary material for: Combined inhibition of Bcl-2 family members and YAP induces synthetic lethality in metastatic gastric cancer with RASA1 and NF2 deficiency
Source: Mol Cancer. 2023 Sep 20;22:156. doi: 10.1186/s12943-023-01857-0 (PMC10510129; doi:10.1186/s12943-023-01857-0)
Supplement: Supplementary file 14 — Additional file 14: Supplemental Figure 9. Evaluation of Wnt and YAP signaling in Nf2- and Rasa1-KO peritoneal dissemination models. [file 12943_2023_1857_MOESM14_ESM.pdf]

Supplemental Figure 9

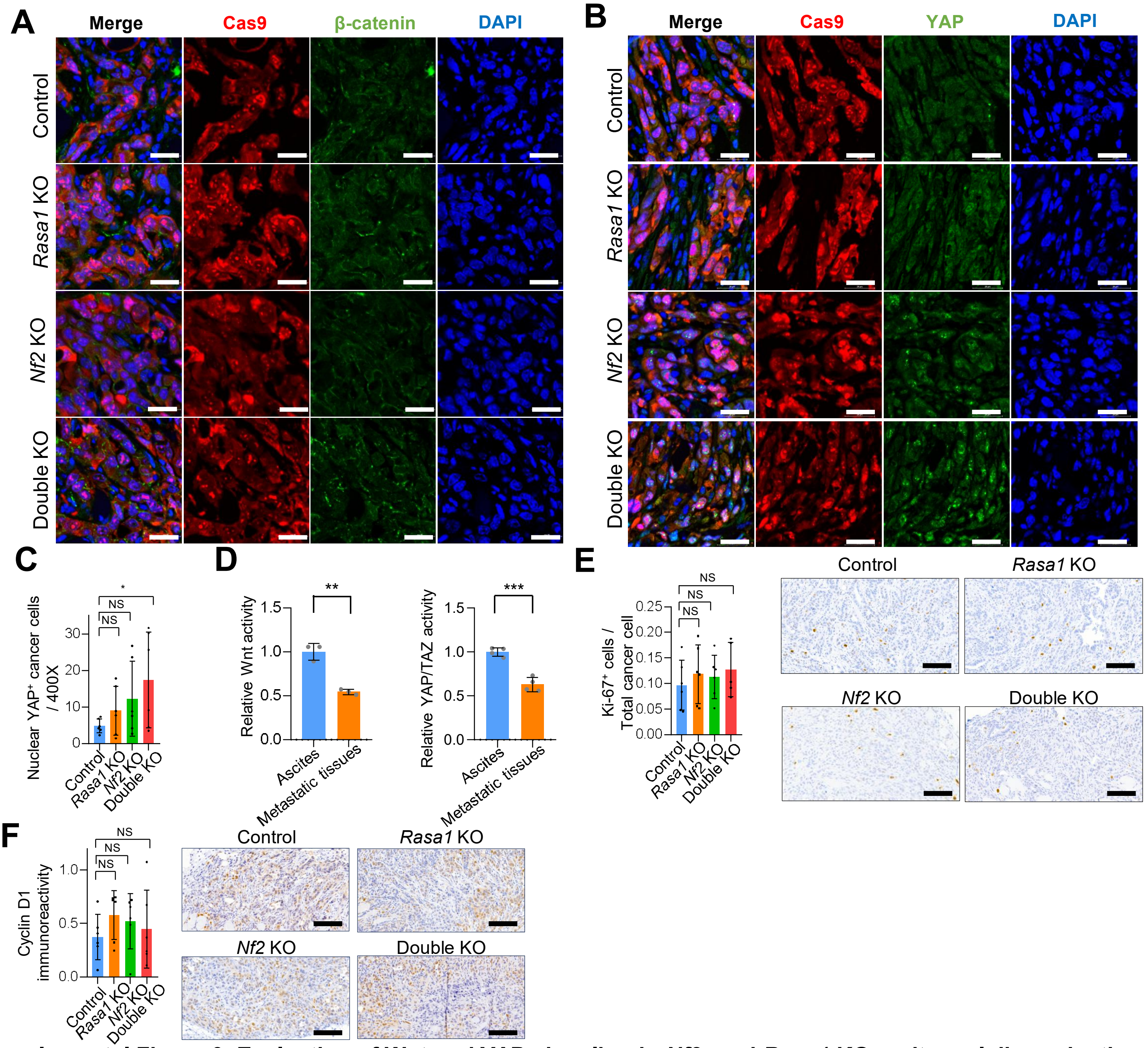

Supplemental Figure 9. Evaluation of Wnt and YAP signaling in *Nf2*- and *Rasa1*-KO peritoneal dissemination models.

(**A**) Representative immunofluorescence staining images for Cas9 (red), active-β-catenin (green) and DAPI in peritoneal metastatic foci of control (*n* = 6), *Rasa1*-KO (*n* = 6), *Nf2*-KO (*n* = 5), and *Rasa1*/*Nf2*-double-KO (*n* = 5) S1M cell peritoneally injected in NOD-SCID mice. Cas9 was used as a cancer cell indicator. Bar = 25 μm

(**B**) Representative immunofluorescence staining images for Cas9 (red), YAP (green), and DAPI in peritoneal metastatic foci of control (*n* = 6), *Rasa1*-KO (*n* = 6), *Nf2*-KO (*n* = 5), and *Rasa1*/*Nf2*-double-KO (*n* = 5) S1M cell peritoneally injected in NOD-SCID mice. Cas9 was used as a cancer cell indicator. Bar = 25 μm

(**C**) Statistical analysis of nuclear accumulation of YAP in peritoneal metastatic foci of control and KO S1M cell peritoneally injected in NOD-SCID mice. $Cas9^{+}$ cancer cells with nuclear staining of YAP were counted at multiple 400 magnification views.

(**D**) (**left**) TOP-Flash luciferase reporter assay in single cells obtained from ascites and metastatic tissues of peritoneally injected S1M cells in NOD-SCID mice. (**right**) HOP-Flash luciferase reporter assay in single cells obtained from ascites and metastatic tissues of peritoneally injected S1M cells in NOD-SCID mice.

(**E** and **F**) IHC analysis of Ki-67^{+} (**E**) and Cyclin $D1^{+}$ (**F**) cancer cells in peritoneal metastatic foci of control, *Rasa1-*, *Nf2*-, and *Rasa1*/*Nf2*-double-KO S1M cell in NOD-SCID mice. Ki-67^{+} cells were counted and divided by the total number of cancer cells using QuPath. Cyclin $D1^{+}$ cells were analyzed using QuPath with H-Score, number of positive cells multiplied by signal strength score, and divided by total number of cancer cells in the analyzed area. Bar = 100 μm. Student's t-test was used for statistical analysis.
