# Supplementary material for: Combined inhibition of Bcl-2 family members and YAP induces synthetic lethality in metastatic gastric cancer with RASA1 and NF2 deficiency
Source: Mol Cancer. 2023 Sep 20;22:156. doi: 10.1186/s12943-023-01857-0 (PMC10510129; doi:10.1186/s12943-023-01857-0)
Supplement: Supplementary file 15 — Additional file 15: Supplemental Figure 10. Examination of lung metastasis in a subcutaneous transplantation model using Rasa1-KO S1M cells. [file 12943_2023_1857_MOESM15_ESM.pdf]

Supplemental Figure 10

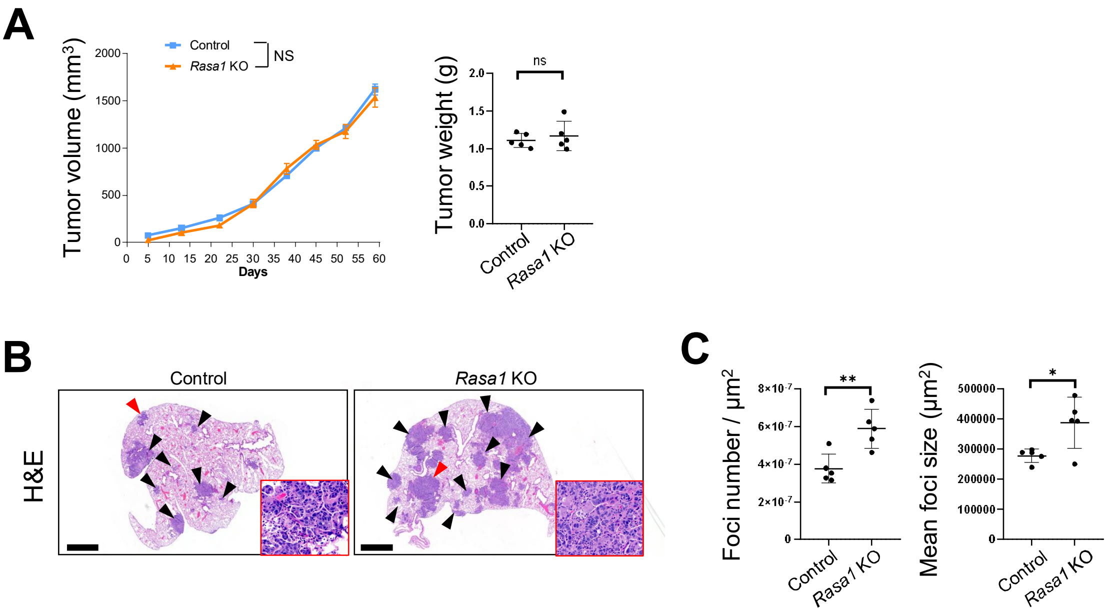

Supplemental Figure 10. Examination of lung metastasis in a subcutaneous transplantation model using *Rasa1*-KO S1M cells.

(**A**) Subcutaneous allograft of control (n = 5) and *Rasa1*-KO (n = 5) S1M cells in NOD-SCID mice was performed to compare pulmonary metastasis. 3×10^6 cells of control and *Rasa1*-KO S1M cells were suspended in 100 µl of DPBS and injected at the dorsal flank area. Necropsy was performed at 9 weeks post-injection. (**left**) The tumor growth curve (mm^3) in NOD-SCID mice with subcutaneous transplantation of control (n = 5) and *Rasa1*-KO (n = 5) S1M cells. The tumor volume was measured every 7 days. (**right**) The weight of the tumor was measured at the time of necropsy. Student's t-test was used for statistical analysis.

(**B** and **C**) (**B**) Representative histopathologic images of pulmonary metastasis in NOD-SCID mice with subcutaneous transplantation of control (n = 5) and *Rasa1*-KO (n = 5) S1M cells. Bar = 1 mm. The red box represents a magnified view of the red arrow location. (**C**) Statistical analysis of pulmonary metastasis in NOD-SCID mice subcutaneously transplanted with control and *Rasa1*-KO S1M cells. The micro-metastatic foci number per total lung area and the mean foci size ($µm^2$) were measured using QuPath.

Statistical analysis was performed using Student's t-test.
