# Supplementary material for: Combined inhibition of Bcl-2 family members and YAP induces synthetic lethality in metastatic gastric cancer with RASA1 and NF2 deficiency
Source: Mol Cancer. 2023 Sep 20;22:156. doi: 10.1186/s12943-023-01857-0 (PMC10510129; doi:10.1186/s12943-023-01857-0)
Supplement: Supplementary file 16 — Additional file 16: Supplemental Figure 11. Immunohistochemical analysis of NF2 and β-catenin in human GC tissues. [file 12943_2023_1857_MOESM16_ESM.pdf]

Supplemental Figure 11

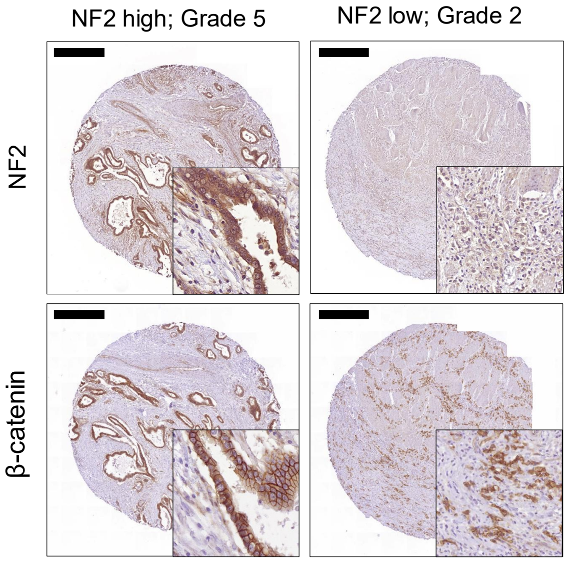

Supplemental Figure 11. Immunohistochemical analysis of NF2 and β-catenin in human GC tissues.

Representative immunohistochemical images of NF2 and β-catenin in human gastric cancer tissue microarray (TMA) samples. The IHC grading was based on the combined assessment of staining strength and positive cell percentages, with 5 grades (1 to 5) assigned. For β-catenin, the IHC stain pattern of nuclear staining and membrane staining was graded separately. Bar = 500 μm
