# Supplementary material for: Combined inhibition of Bcl-2 family members and YAP induces synthetic lethality in metastatic gastric cancer with RASA1 and NF2 deficiency
Source: Mol Cancer. 2023 Sep 20;22:156. doi: 10.1186/s12943-023-01857-0 (PMC10510129; doi:10.1186/s12943-023-01857-0)
Supplement: Supplementary file 17 — Additional file 17: Supplemental Figure 12. Analysis of Wnt inhibitor expression in response to Nf2-KO and YAP/TAZ signaling modulation. [file 12943_2023_1857_MOESM17_ESM.pdf]

Supplemental Figure 12

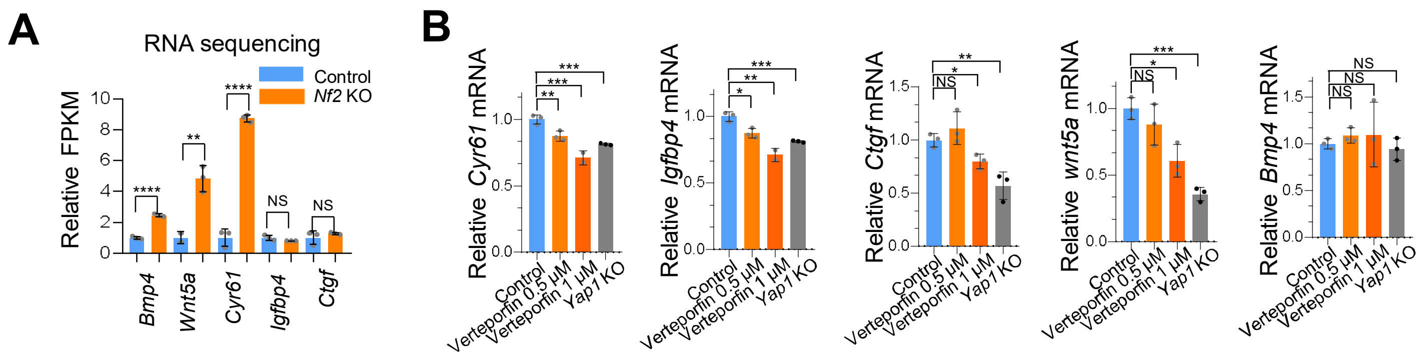

Supplemental Figure 12. Analysis of Wnt inhibitor expression in response to *Nf2*-KO and YAP/TAZ signaling modulation.

(**A**) RNA sequencing results of S1 tumorspheres of control and *Nf2*-KO. The relative mRNA expression of Wnt inhibitors (*Bmp4*, *Wnt5a*, *Cyr61, Igfbp4* and *Ctgf)* was compared using Student's T test.

(**B**) Reverse transcriptase-quantitative PCR analyses in S1 cells. For S1 control cells, vehicle and verteporfin (0.5 and 1.0 μM) were treated for 48 h. *Yap1* KO S1 cells were cultured in RPMI-1640 media supplemented with 10% FBS and 1% PS. The relative mRNA expression of Wnt inhibitors (*Cyr61, Igfbp4, Ctgf*, *Wnt5a*, and *Bmp4)* were analyzed using Student's T test.
