# Supplementary material for: Combined inhibition of Bcl-2 family members and YAP induces synthetic lethality in metastatic gastric cancer with RASA1 and NF2 deficiency
Source: Mol Cancer. 2023 Sep 20;22:156. doi: 10.1186/s12943-023-01857-0 (PMC10510129; doi:10.1186/s12943-023-01857-0)
Supplement: Supplementary file 18 — Additional file 18: Supplemental Figure 13. Immunofluorescence staining analysis for Bcl-2, Bcl-xL, β-catenin, and Yap1 in control, Rasa1-KO, Nf2-KO, and Rasa1/Nf2-double-KO peritoneal metastatic foci. [file 12943_2023_1857_MOESM18_ESM.pdf]

Supplemental Figure 13

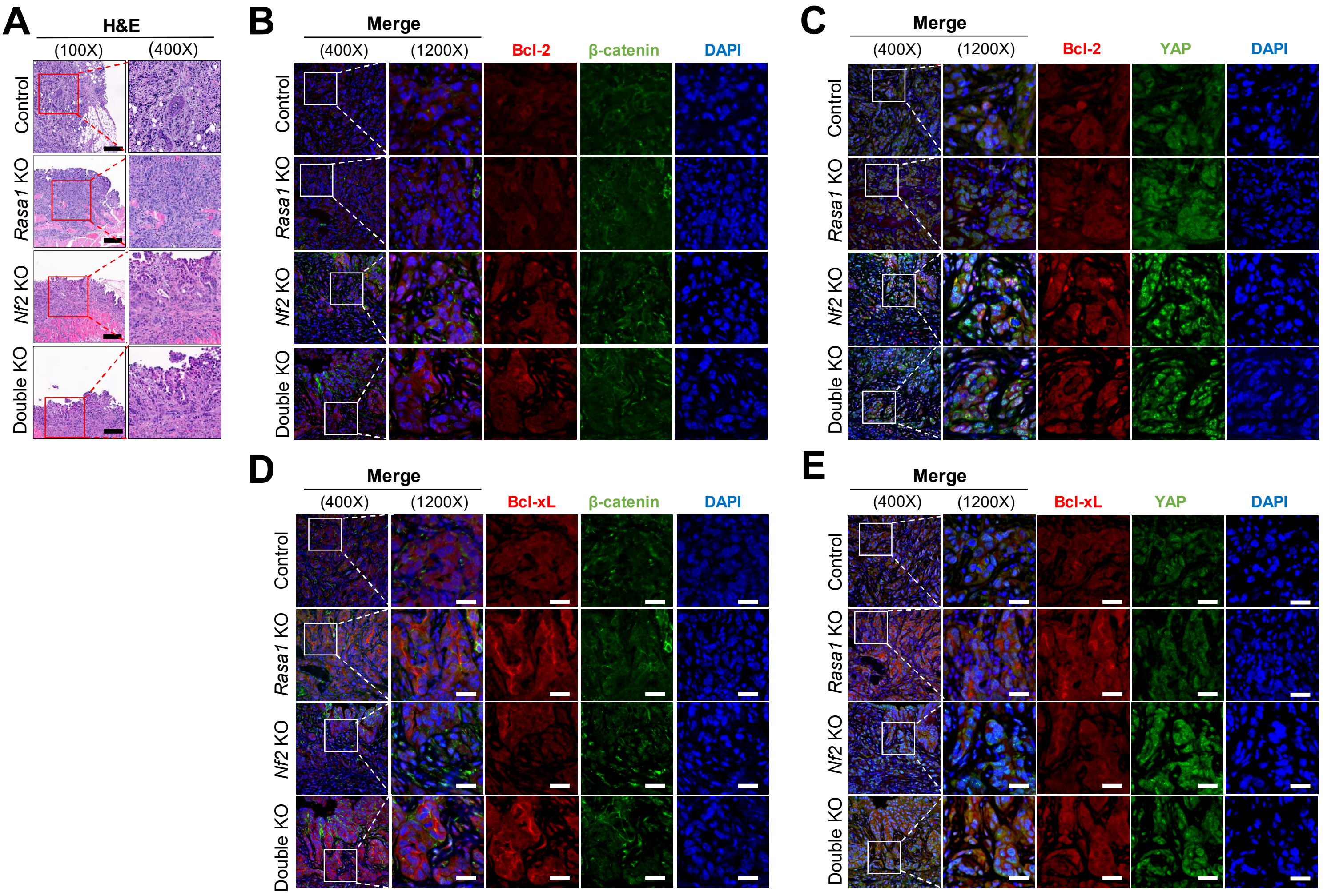

Supplemental Figure 13. Immunofluorescence staining analysis for Bcl-2, Bcl-xL, β-catenin, and Yap1 in control, Rasa1-KO, Nf2-KO, and Rasa1/Nf2-double-KO peritoneal metastatic foci.

The tissues analyzed were sourced from the peritoneal dissemination models presented in Figure 3B-F. Using consecutive paraffin sections, we investigated the expression of each protein in cancer cells from the same location.

(A) H&E staining of metastatic foci, with the right panel presenting an enlarged view of the boxed region on the left. Bar = 200 μm.

(B-E) Representative immunofluorescence images displaying **(B)** Bcl-2 (red) paired with active-β-catenin (green), **(C)** Bcl-2 (red) paired with YAP (green), **(D)** Bcl-xL (red) paired with active-β-catenin (green), **(E)** Bcl-xL (red) paired with YAP (green) for control, *Rasa1*-KO, *Nf2*-KO, and *Rasa1*/*Nf2*-double-KO metastatic foci. Bar = 25 μm.
