# Supplementary material for: Combined inhibition of Bcl-2 family members and YAP induces synthetic lethality in metastatic gastric cancer with RASA1 and NF2 deficiency
Source: Mol Cancer. 2023 Sep 20;22:156. doi: 10.1186/s12943-023-01857-0 (PMC10510129; doi:10.1186/s12943-023-01857-0)
Supplement: Supplementary file 19 — Additional file 19: Supplemental Figure 14. Immunohistochemical analysis following YAP and Bcl-2 inhibitor treatment in Nf2/Rasa1-double KO S1M peritoneal dissemination model. [file 12943_2023_1857_MOESM19_ESM.pdf]

Supplemental Figure 14

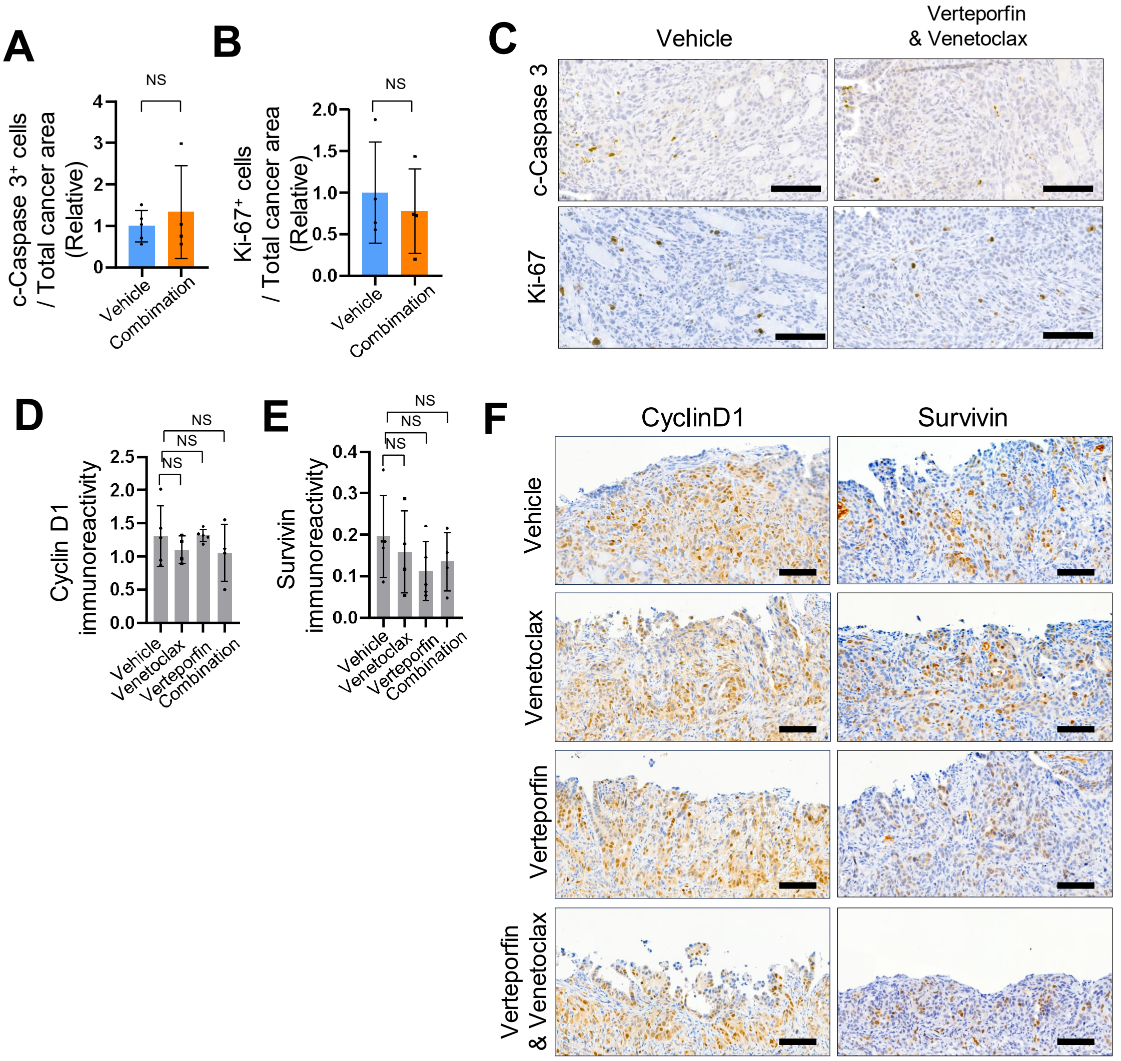

Supplemental Figure 14. Immunohistochemical analysis following YAP and Bcl-2 inhibitor treatment in *Nf2*/*Rasa1*-double KO S1M peritoneal dissemination model.

(**A**, **B**, and **C**) Statistical analysis of IHC and representative images in peritoneal metastatic foci of intraperitoneally injected with *Nf2*-KO S1M cells in NOD-SCID mice, treated with vehicle (n = 4) and venetoclax/verteporfin combination (n = 4). Student's T test was used in comparison with vehicle-treated group and combination treatment of verteporfin (10 mg/kg, i.p) and venetoclax (12 mg/kg, p.o. route) group. (**A** and **B**) Statistical analysis of IHC using (**A**) cleaved-Caspase 3 and (**B**) Ki-67. c-Caspase 3 and Ki-67 positive cells were counted using QuPath and divided by total cancer area (μm^2). The result was normalized to the vehicle treatment group. (**C**) Representative images of IHC using (**A**) c-Caspase 3 and (**B**) Ki-67. Bar = 100 μm

(**D**, **E**, and **F**) Statistical analysis of IHC and representative images in peritoneal metastatic foci of intraperitoneally injected with *Nf2*-KO S1M cells in NOD-SCID mice, treated with vehicle (n = 5), venetoclax (n = 4, 12 mg/kg, p.o. route, once a day), verteporfin (n = 5, 10 mg/kg, i.p, every other day) and venetoclax/verteporfin combination (n = 4). Student's T test was used in comparison with vehicle-treated group and other groups. (**D** and **E**) Statistical analysis of IHC using QuPath with H-Score of (**D**) Cyclin D1 and (**E**) Survivin. For Cyclin D1 and Survivin, positive cells were counted, and the signal strength score was multiplied and divided by the total number of cancer cells. (**F**) Representative images of IHC using (**D**) Cyclin D1 and (**E**) Survivin. Bar = 100 μm
