# Supplementary material for: Combined inhibition of Bcl-2 family members and YAP induces synthetic lethality in metastatic gastric cancer with RASA1 and NF2 deficiency
Source: Mol Cancer. 2023 Sep 20;22:156. doi: 10.1186/s12943-023-01857-0 (PMC10510129; doi:10.1186/s12943-023-01857-0)
Supplement: Supplementary file 20 — Additional file 20: Supplemental Figure 15. Immunohistochemical analysis following YAP and Bcl-xL inhibitor treatment in Nf2/Rasa1-double KO S1M peritoneal dissemination model. [file 12943_2023_1857_MOESM20_ESM.pdf]

Supplemental Figure 15

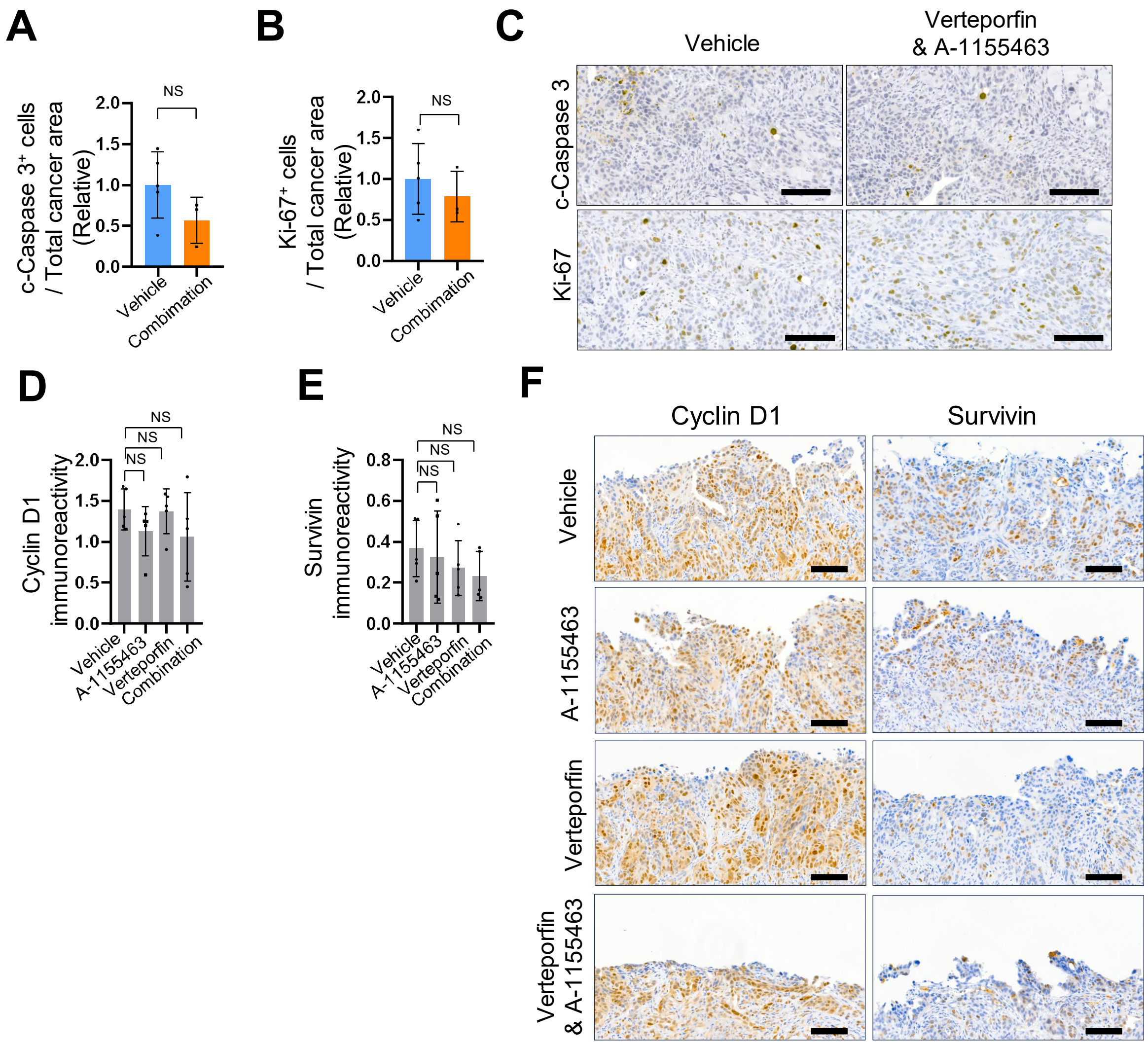

Supplemental Figure 15. Immunohistochemical analysis following YAP and Bcl-xL inhibitor treatment in *Nf2*/*Rasa1*-double KO S1M peritoneal dissemination model.

(**A**, **B**, and **C**) Statistical analysis of IHC and representative images of peritoneal metastatic foci in NOD-SCID mice intraperitoneally injected with *Nf2*/*Rasa1*-double KO S1M cells, treated with vehicle (*n* = 5) and combination of verteporfin and A-1155463 (*n* = 3). 5 × 10^6 *Nf2*/*Rasa1*-double KO S1M cells were intraperitoneally injected in NOD-SCID mice and allowed to develop the peritoneal metastasis for 7 days. Vehicle, verteporfin (10 mg/kg, i.p.every other day) and A-1155463 (7.5 mg/kg, i.p. once a day) combination treatment was conducted. (**A** and **B**) Statistical analysis of IHC using (**A**) cleaved-Caspase 3 and (**B**) Ki-67. c-Caspase 3 and Ki-67 positive cells were counted using QuPath and divided by total cancer area (μm^2). The result was normalized to the vehicle treatment group. (**C**) Representative images of IHC using (**A**) c-Caspase 3 and (**B**) Ki-67. Bar = 100 μm

(**D**, **E**, and **F**) Statistical analysis of IHC and representative images of peritoneal metastatic foci in NOD-SCID mice intraperitoneally injected with *Nf2*/*Rasa1*-double KO S1M cells, treated with vehicle (*n* = 5), A-1155463 (*n* = 5), verteporfin (*n* = 5), and combination of verteporfin and A-1155463 (*n* = 5). 5 × 10^6 *Nf2*/*Rasa1*-double KO S1M cells were intraperitoneally injected in NOD-SCID mice and allowed to develop the peritoneal metastasis for 7 days. Vehicle, verteporfin (10 mg/kg, i.p.), A-1155463 (7.5 mg/kg, i.p.), and verteporfin/A-115463 combination treatment was conducted. (**D** and **E**) Statistical analysis of IHC using QuPath with H-Score of (**D**) Cyclin D1 and (**E**) Survivin. For Cyclin D1 and Survivin, positive cells were counted, and the signal strength score was multiplied and divided by the total number of cancer cells. (**F**) Representative images of IHC using (**D**) Cyclin D1 and (**E**) Survivin. Bar = 100 μm
